# Bandicoot fossils and DNA elucidate lineage antiquity amongst xeric-adapted Australasian marsupials

**DOI:** 10.1038/srep37537

**Published:** 2016-11-24

**Authors:** Benjamin P. Kear, Ken P. Aplin, Michael Westerman

**Affiliations:** 1 Museum of Evolution, Uppsala University, Norbyvägen 16, SE-752 36 Uppsala, Sweden; 2 Department of Earth Sciences, Uppsala University, Villavägen 16, SE-752 36 Uppsala, Sweden; 3 Division of Mammals, National Museum of Natural History, Smithsonian Institution, P.O. Box. 37012, Washington, DC, 20013-7012, USA; 4 Department of Ecology, Environment and Evolution, La Trobe University, Melbourne, Victoria 3086, Australia

## Abstract

Bandicoots (Peramelemorphia) are a unique order of Australasian marsupials whose sparse fossil record has been used as prima facie evidence for climate change coincident faunal turnover. In particular, the hypothesized replacement of ancient rainforest-dwelling extinct lineages by antecedents of xeric-tolerant extant taxa during the late Miocene (~10 Ma) has been advocated as a broader pattern evident amongst other marsupial clades. Problematically, however, this is in persistent conflict with DNA phylogenies. We therefore determine the pattern and timing of bandicoot evolution using the first combined morphological + DNA sequence dataset of Peramelemorphia. In addition, we document a remarkably archaic new fossil peramelemorphian taxon that inhabited a latest Quaternary mosaic savannah-riparian forest ecosystem on the Aru Islands of Eastern Indonesia. Our phylogenetic analyses reveal that unsuspected dental homoplasy and the detrimental effects of missing data collectively obscure stem bandicoot relationships. Nevertheless, recalibrated molecular clocks and multiple ancestral area optimizations unanimously infer an early diversification of modern xeric-adapted forms. These probably originated during the late Palaeogene (30–40 Ma) alongside progenitors of other desert marsupials, and thus occupied seasonally dry heterogenous habitats long before the onset of late Neogene aridity.

Bandicoots (Peramelemorphia) are a speciose order of Australasian marsupials that appeared early in the evolutionary history of Australidelphia^1^. Most are small to medium sized (up to 5 kg) terrestrial omnivores occupying a spectrum of rainforest to desert habitats^2^,^3^. Molecular studies^1^,^4^ have defined three taxonomic subdivisions within the crown superfamily Perameloidea (Fig. 1): Chaeropodidae (pig-footed bandicoots), comprising the Central Australian dry-grassland, and possibly herbivorous *Chaeropus ecaudatus*, which became extinct as recently as the 1950’s^5^; Thylacomyidae (bilbies), a monogeneric classification for the genetically divergent^6^ *Macrotis*, which occurs in the arid and semi-arid zones of Australia^7^; and Peramelidae (typical bandicoots), an ecologically diverse radiation incorporating the primarily Australian Peramelinae and New Guinean (including surrounding islands and tropical far northern Australia) Peroryctinae and Echymiperinae^8^. DNA-based cladogenic scenarios for these groups envisage a latest Oligocene–early Miocene split (~20–30 Ma) between the xeric-adapted chaeropodids and thylacomyids versus predominantly mesic peramelids^1^,^4^. This contrasts with published morphological data, which posits both a late Miocene–Pliocene diversification of Australian Peramelinae concurrent with increasing aridity^9^,^10^, and late Miocene vicariant origins for Peroryctinae and Echymiperinae (historically united as Peroryctidae)^11^ accompanying New Guinean tectonic uplift. Such arguments are inferred from the fossil record, which has yielded no generically referable crown taxa older than the early Pliocene^3^,^11^. Fossil evidence of extant higher-level clades is also extremely sparse, consisting of isolated dental remnants from the middle Miocene putative basal peramelid *Crash*^12^ and thylacomyid *Liyamayi*^12^, as well as cranial-mandibular elements of the early–middle Miocene stem perameloids *Madju*^13^ and *Kutjamarcoot*^14^. Conversely, all other pre-Pliocene peramelemorphians are usually placed outside of Perameloidea, including the early Miocene *Bulungu*^15^,^16^, *Galadi*^17^,^18^, and a markedly plesiomorphic family-level grouping Yaralidae, which is composed of two species, *Yarala burchfieldi* from the early–middle Miocene^19^,^20^, and the late Oligocene *Y. kida*^21^. Yaralids are traditionally considered the basal sister radiation (=Yaraloidea)^20^ to all other bandicoots, and are thus important for calibrating molecular clocks within Peramelemorphia^1^,^4^ and Marsupialia as a whole^22^,^23^,^24^. However, yaralids are united by a single unique symplesiomorphy – retention of a ‘complete’ centrocrista formed by the postparacrista and premetacrista on the upper molars^21^. This has since been identified in a range of Oligocene−Miocene bandicoots^16^,^17^,^18^, and is ancestral amongst marsupials^25^, but otherwise lost in crown perameloids (defined by separated postparacristae-premetacristae) prompting assertions of a Miocene ‘bottleneck’ within Peramelemorphia, as well as other Australasian marsupials^26^, whereby the radiation of crown taxa occurred only after the decline of rainforest-restricted stem forms, perhaps in response to climate change, ecological competition and/or vicariance events^3^,^9^,^10^,^15^,^19^,^20^,^21^,^22^,^23^,^24^,^26^,^27^.

Here we test the long-standing hypotheses of Miocene faunal turnover and the recent origin of modern Australasian marsupials, by using the first total evidence morphological + multi-gene sequence dataset of Peramelemorphia incorporating examplars of all extinct and living genera named in the published literature. We also report on a remarkably primitive new fossil bandicoot from cave deposits on Pulau Kobroor in the Aru Islands, which has been radiocarbon and U-series dated to the latest Pleistocene–Holocene (28–9 Ka)^28^,^29^,^30^,^31^. This significant discovery represents the geologically youngest stem-grade peramelemorphian, and implies not only extreme longevity but also undetected complexity affecting state acquisitions within this pivotal australidelphian order. Furthermore, our improved molecular clock constraints and coupled ancestral area analyses enable more precise timing for the nascent dispersal of crown bandicoots into open mosaic habitats, and thus refines burgeoning DNA-based evidence for Australia’s modern arid-zone marsupials as members of enduring, adaptable clades^32^.

## Results

Systematic Palaeontology

Marsupialia (Illiger, 1811) Cuvier, 1817

Australidelphia Szalay, 1982

Peramelemorphia (Kirsch, 1968) Aplin & Archer, 1987

*Lemdubuoryctes aruensis* gen. et sp. nov.

### Diagnosis

*Lemdubuoryctes* is distinguished from all currently extant bandicoot genera (plus *Chaeropus*), the early Pliocene *cf. Peroryctes tedfordi*^33^, early–middle Miocene *Kutjamarcoot*^14^, *Madju*^13^, *Liyamayi*^12^, *Crash*^12^, *Galadi amplus*^18^, and late Oligocene *Bulungu campbelli*^16^ by its retention of a ‘complete’ centrocrista with continuous postparacristae-premetacristae on all upper molars. The centrocrista is incomplete on M3 of the early Miocene *Galadi speciosus*^17^, and is formed by residual buccal crests on M3 of the early Miocene *Bulungu palara*^15^. *Galadi grandis*^18^ also from the early Miocene, and *Bulungu muirheadae*^16^ the oldest known late Oligocene bandicoot, possess ‘complete’ centrocristae along their upper molar rows but differ from *Lemdubuoryctes* in the presence of two mental foramina on the dentary, and the absence of anterior cingulae on M2–4 respectively. The lack of an elevated talonid separates *Lemdubuoryctes* from the latest Miocene–early Pliocene *Ischnodon*. Finally, oblique orientation of the posthypocristid relative to the lower molar row contrasts with late Oligocene–early Miocene *Yarala*^19^,^21^.

### Etymology

‘Lemdubu’ from the type locality, and ‘oryctes’ (*ορυκτης*, masculine) for ‘digger’; species name refers to its endemic occurrence on the Aru Islands.

### Holotype

WAM 14.9.6, left maxilla containing P3 and M1–2.

### Additional material

WAM 14.9.1–WAM 14.9.5 and WAM 14.9.7–WAM 14.9.20 (dentigerous elements, petrosals and calcanea).

### Type locality and horizon

Liang Lemdubu cave (‘Layer 4’, Spit 24) on Pulau Kobroor, Aru Islands group, Eastern Indonesia^29^. Additional material was recovered from Spit 6, 14 and 18–25 (‘Layers 3–5’) at Liang Lemdubu, and Spits 33–40 (‘Layer 4’) in a second cave on Pulau Kobroor – Liang Nabulei Lisa^29^. A combination of radiocarbon and U-series age determinations place these deposits within the latest Pleistocene to Holocene, between *ca* 28,000–9,000 BP for Liang Lemdubu, and *ca* 16,200–12,000 BP for Liang Nabulei Lisa^30^,^31^.

#### Description of the new fossil taxon

Both the holotype (Western Australian Museum [WAM] 14.9.6) and referred (WAM 14.9.9) maxillae of *Lemdubuoryctes* (Fig. 2) display tooth eruption and molar wear indicative of adult animals (this is most extreme in WAM 14.9.9). The remnant palatal shelf on WAM 14.9.9 preserves a vacuity in the molar region. The base of the zygomatic arch is level with the alveolar margin. The antorbital fossa extends posteriorly from above the M3 to behind the M4; this differs from many extant peramelemorphians, as well as *Bulungu palara*^15^ and *Galadi speciosus*^17^, but can be intraspecifically variable^13^. Posterior expansion of the antorbital fossa otherwise occurs only in living *Peroryctes*^34^ (Supplementary Fig. S1), some *Echymipera rufescens* (e.g. Australian Museum [AM] S1866)^35^, *Yarala burchfieldi*^20^, and osteologically mature *Madju*^13^ specimens. The antorbital fossa of *Macrotis* is uniquely elevated above the tooth row^11^. The anterior opening of the infraorbital canal in WAM 14.9.6 extends to the P3 alveolus (or the posterior margin of the M1 in WAM 14.9.9). This is similar to most bandicoots (including *B. palara*)^15^, in which the infraorbital canal usually has an expansive exit over the M1–P3. The infraorbital canal opens immediately above the P3 in *Y. burchfieldi*^20^ and species of *Galadi*^17^,^18^.

The upper premolars of *Lemdubuoryctes* are double-rooted with diastemata between P1 and P2 (WAM 14.9.11), suggesting an elongate maxillary rostrum. The P3 exhibits marked size dimorphism consistent with sexual variation observed in some extant peramelids, most notably species of *Peroryctes* and *Echymipera*^34^ (Supplementary Fig. S1). We therefore interpret WAM 14.9.6 as a probable male because the P3 exceeds the M1 in occlusal area (Fig. 2B). The P3 is smaller than the M1 in WAM 14.9.9 and thus represents a potential female (Supplementary Table S1). Both P3 morphotypes are otherwise identical in their triangular basal outline with conical central cusp, weak posterolingual cingulum and incipient anterobasal cuspule.

At up to 14.89 mm in length (Supplementary Table S1), the complete M1–M4 row of *Lemdubuoryctes* (WAM 14.9.9) is equal to the largest living bandicoot *Peroryctes broadbenti*^34^. The M1 (Fig. 2; Supplementary Fig. S2) is triangular in occlusal outline unlike the more quadrangular molars of peramelines, *Chaeropus* and *Macrotis*; the latter further distinguished by extreme lingual displacement of the metacone^27^. In *Lemdubuoryctes*, the metacone is positioned at the posterolabial margin of the trigon basin, which is bounded buccally by the paracone. A prominent protocone is situated lingually. There is no protoconule. The metaconule (metaconular hypocone) forms only a weak spur that connects the postprotocrista to the base of the metacone. This is compatible with extreme metaconular reduction seen in the stem peramelemorphians *Yarala*^19^,^21^, *Bulungu*^15^,^16^, and *Galadi*^17^,^18^, together with the early Pliocene *cf. Peroryctes tedfordi*^33^. Alternatively, living bandicoots^27^ as well as species of *Crash*^12^, *Madju*^13^ and *Kutjamarcoot*^14^ elaborate the metaconule into an enamel flange that is demarcated from the protocone via a vertical trough (Supplementary Fig. S1). Dasyuromorphian marsupial carnivores have a more prominent cusp-like metaconule^36^. Amongst bandicoots only *Macrotis* completely lacks a metaconular structure, but a small metaconule is present in the putative thylacomyid *Liyamayi*^12^.

The anterior cingulum on the M1 of *Lemdubuoryctes* is formed by the preprotocrista, which connects to the parastylar base. There is no posterior cingulum. The paracone lies directly behind the parastyle and the preparacrista runs posterobuccally towards the parastylar tip (a common trait amongst Peramelemorphia: see Supplementary Data, character 14). The postparacrista merges with the premetacrista to create a ‘complete’ centrocrista. The opposing buccal ectoflexus is shallowly incised between the remnants of stylar cusps B and D (there is no discernible stylar cusp E). Remarkably, there are broad ectolophs evident on the M2 and M3 that closely resemble those of the most ancient peramelemorphians *Yarala kida*^21^ and *Bulungu muirheadae*^16^. In other fossils, the postparacrista and premetacrista gradually retract resulting in a ridge-like centrocrista on the M3 of *B. palara*^15^, and total division of the ectoloph in *Bulungu campbelli*^16^, *Galadi*^17^,^18^, *Madju*^13^ and *Kutjamarcoot*^14^. Remnants of the centrocrista also occur on the M1–3 of extant *P. broadbenti* (Supplementary Fig. S1), less prominently on the M1–2 of *Peroryctes raffrayana*, and occasionally in *E. rufescens*^34^. Crests appear elsewhere on the M1–2 of *Crash*^12^ and *cf. P. tedfordi*^33^, which also has a small ‘stylar cusp C’, perhaps constituting another residual component.

The M2 and M3 of *Lemdubuoryctes* (Fig. 2; Supplementary Fig. S2) differ from the M1 in their lingually positioned paracone, less distinct ectoflexus, and transversely oriented preparacrista that trends towards stylar cusp B, but retains contact with the parastylar tip via a subsidiary crest. This forms a truncated anterior cingulum comparable to that on the M2–3 of *P. raffrayana*^34^.

The M4 of *Lemdubuoryctes* (Fig. 2; Supplementary Fig. S2) is reduced relative to the anterior molars and bears both a paracone and diminutive protocone. An anterobuccal cingulum is not visible but could be covered by matrix in WAM 14.9.9. The postprotocrista forms the posterior margin of the tooth and meets the postparacrista at stylar cups B.

Mandibular elements were referred to *Lemdubuoryctes* based on obvious morphological distinction from the sympatric bandicoots^29^ *Isoodon macrourus*, *E. rufescens* and *E. kalubu.* Relative hypertrophy of the p3 differentiates presumed male (WAM 14.9.1) and female (WAM 14.9.3) specimens (Fig. 3; Supplementary Fig. S3). The mandibular rami of *Lemdubuoryctes* (Supplementary Fig. S4) are ventrally convex and up to 9.7 mm deep below the m3 (WAM 14.9.3). The single mental foramen is level with the midline of p1, and the mandibular symphysis extends to the middle of p2. The ascending ramus in WAM 14.9.1 is angled at ~45°; the mandibular foramen opens low on the medial surface and the masseteric fossa is well defined. The i3 root on WAM 14.9.3 is separated from the canine alveolus (3.9/1.8 mm in maximum length/width) by a 2 mm diastema. Another diastema (3.5 mm) intersperses between c1 and p1 with a narrower gap between p1 and p2. The length and height of p1–3 decrease anteriorly (Supplementary Table S2) and are coupled with progressive migration of the blade-like central cusp forward over the anterior root. There are no accessory cuspids.

The complete m1–4 row of *Lemdubuoryctes* (Fig. 3; Supplementary Fig. S3) was up to 17.43 mm long (WAM 14.9.3: Supplementary Table S2), with marked constriction evident at the enamel crown–root interface (also visible on p1–3); this is typical of peramelemorphians except for *Isoodon* and *Macrotis*^27^. The m1 is laterally compressed with a bulbous trigonid incorporating a prominent paraconid, which is absent in *Ischnodon*^37^, living peramelines and *Echymipera*^27^. There are no median buccal cuspules between the trigonids and talonids as reported in *Y. burchfieldi*^19^. The anterior cingulid is reduced on m1–4 and the labial cingulids are weakly developed, similar to *Peroryctes*^34^ (Supplementary Fig. S1). The cristid obliqua terminates buccally against the posterior wall of the protoconid on m1–2 (rather than the metaconid as in many peramelines^27^,^38^) but is more lingually positioned on the m3, and immediately adjacent to the metacristid notch on m4. The hypoconulid is situated directly posterior to the entoconid, and sunken well below the talonid basin on all lower molars (synapomorphies for Peramelemorphia^27^). The posthypocristid is oblique to the molar row like that of *P. raffrayana*^34^ (Supplementary Fig. S1). The conical entoconids on m2–3 differ from the blade-like structures in *P. raffrayana*^34^ (Supplementary Fig. S1), some species of *Microperoryctes* (Supplementary Data, character 25), *cf. P. tedfordi*^33^ and *Y. kida*^21^. A discrete entoconid and hypoconid on the reduced m4 talonid are additional differences relative to *Y. burchfieldi*^19^ and *B. campbelli*^16^.

The petrosals of *Lemdubuoryctes* (WAM 14.9.13–WAM 14.9.15) were identified by their weakly inflated periotic hypotympanic sinuses (thus excluding *I. macrourus*^27^), and large size relative to those of *I. macrourus*, *E. rufescens* and *E. kalubu*. They also bear small rostral tympanic and caudal tympanic processes, as well as shallow mastoid sinuses and epitympanic recesses (Supplementary Fig. S5). The prominent ventral flange on the pars cochlearis is compatible with those of *Y. burchfieldi*^21^, *B. palara*^15^ and *P. raffrayana*^34^.

Calcanea were assigned to *Lemdubuoryctes* on the basis of their substantial size and striking morphological distinction from those of *I. macrourus*, *E. rufescens* and *E. kalubu*. The largest referred calcaneum (WAM 14.9.16: Supplementary Fig. S6) is 22.2 mm in maximum length. The compact tuber calcis, oblique calcaneo-cuboid facet, and projecting calcaneum-austragalus facet incorporating a short triangular lateral shelf, are all reminiscent of the condition in *Microperoryctes*^39^ and *P. raffrayana* (American Museum of Natural History [AMNH] 151936), but contrast with that of *Echymipera*^39^ (Supplementary Fig. S6). Calcanea have not yet been described for any other fossil peramelemorphian.

#### Phylogeny

**Figure 1:**
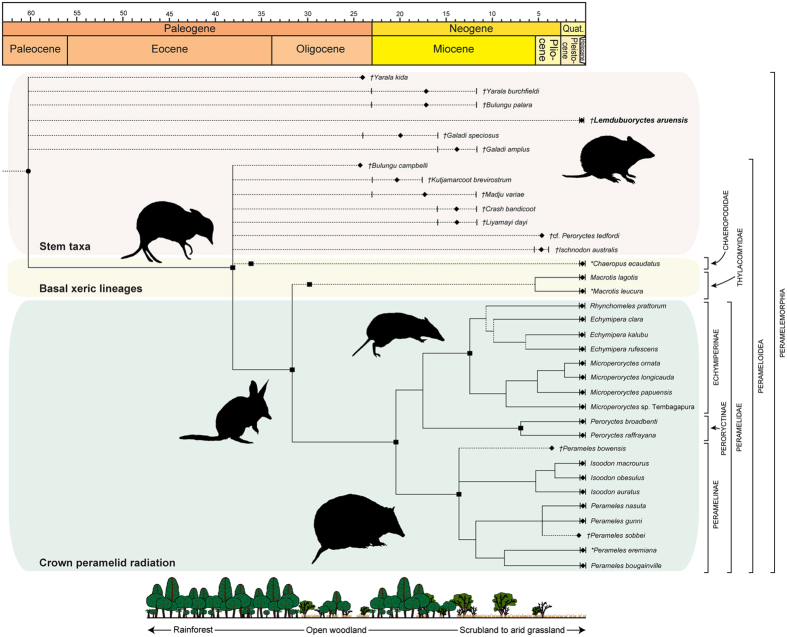
Geochronologically calibrated consensus hypothesis of peramelemorphian interrelationships. Colour gradient demarcates *Lemdubuoryctes aruensis* and other stem-grade bandicoots (pink) from xeric-adapted extant lineages (ochre), and the crown peramelid radiation (green). The peramelemorphian root node (●) and named subclades (■) are also correlated against a schematic of changing habitats through time. Dashed lines indicate phylogenetic uncertainty. ^†^Fossil; *recently extinct arid-zone taxon. Graphics produced with *Adobe* CS5 by B. P. K.

**Figure 2:**
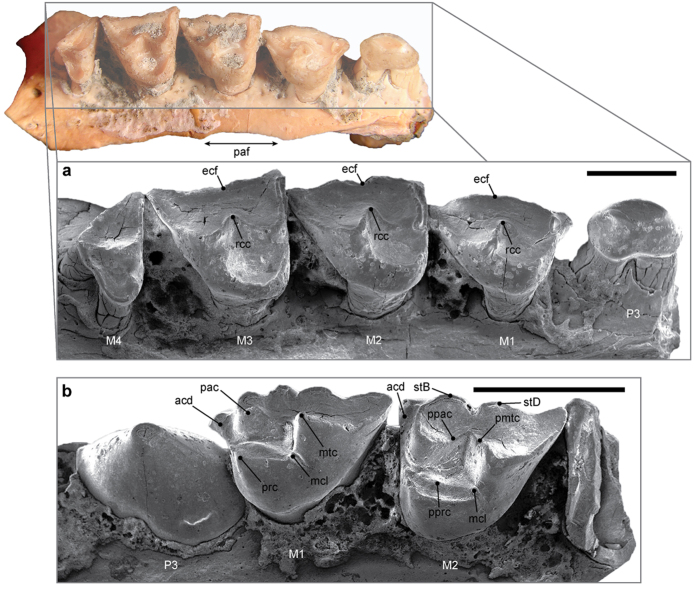
*Lemdubuoryctes aruensis* referred (WAM 14.9.9) and holotype (WAM 14.9.6) maxillae. (**a**) SEM image of P3–M4 from WAM 14.9.9 shown in oblique occlusal view. (**b**) SEM image of the hypertrophied P3 and M1–M2 from WAM 14.9.6 in oblique occlusal view. Scale bars: (**a**) 3 mm, (**b**) 4 mm. Anatomical abbreviations: acd, anterior cingulid; ecf, ectoflexus; mcl, metaconule; mtc, metacone; pac, paracone; paf, area of the palatal fenestra; pmtc, premetacrista; ppac, postparacrista; pprc, postprotocrista; prc, protocone; rcc, residual centrocrista; stB, stylar cusp B; stylar cusp D. Imaging by K.P.A.

**Figure 3:**
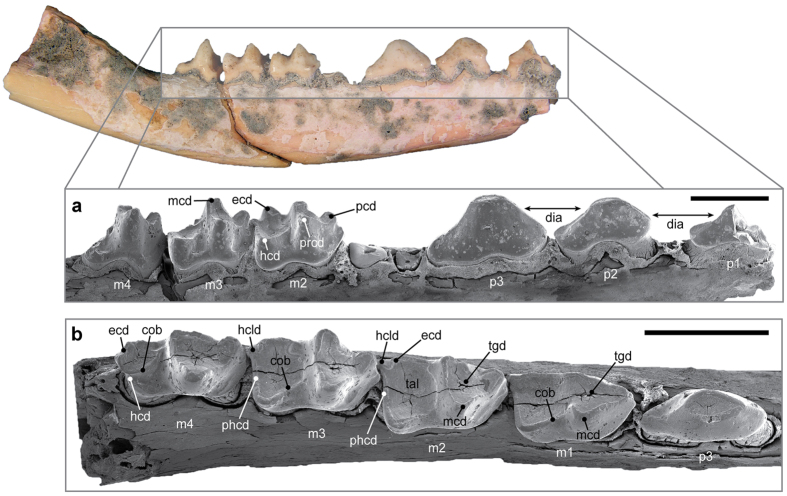
*Lemdubuoryctes aruensis* referred dentaries. (**a**) SEM image of p1–p3, and m2–m4 from WAM 14.9.1 shown in oblique occlusal view. (**b**) p3 and m1–m4 from WAM 14.9.3 in oblique occlusal view. Scale bars: 4 mm. Anatomical abbreviations: cob, cristid obliqua; dia, diastema; ecd, entoconid; hcd, hypoconid; hcld, hypoconulid; mcd, metaconid; pcd, paraconid; phcd, posthypocristid; prcd, protoconid, tal, talonid; tgd, trigonid. Imaging by K.P.A.

Analysis of our morphological dataset (including molecular backbone constraints: Supplementary Fig. S7) produced both poor ingroup resolution and node support for the placement of fossil taxa (Supplementary Figs S8 and S9). We attribute this to missing data and pervasive homoplasy, which was detected during construction of our matrix (see Methods) and subsequently examined via serial pruning of redundant fossils^40^ and wildcards^41^ (Supplementary Figs S10–S12). These procedures returned *Lemdubuoryctes*, together with *Bulungu palara*, *Yarala burchfieldi* and species of *Galadi* as labile stem peramelemorphians. This implies enormously protracted ghost lineages (Fig. 1), but no discrete character states served to unite these taxa as a clade. In fact, the only traits collectively distinguishing basal stem peramelemorphians from more crown-ward bandicoots were the deeply symplesiomorphic^20^,^25^ presence of ‘complete’ centrocristae on M1–3 (although this is polymorphic in *Galadi speciosus*^17^ and absent in *G. amplus*^18^), and an alisphenoid-parietal contact on the lateral wall of the neurocranium (also evident in *Madju*^13^). An alternatively derived squamosal-frontal contact is shared by all crown perameloids, and might be diagnostic for this radiation, but was not a major driver of our topologies (see Supplementary Data). Moreover, the monophyly of *Yarala*^21^ as a separate sister grouping to Perameloidea was equivocal (Supplementary Table S3), leading us to question the taxonomic utility of Yaraloidea based on these primitive states alone^3^,^20^,^21^.

The other fossil bandicoots *Bulungu campbelli*, *Kutjamarcoot*, *Crash* and *cf. Peroryctes tedfordi* were variously interpolated amongst crown Perameloidea, with *Madju* positioned as a sibling lineage. This rendered the genus *Bulungu* polyphyletic (although constraint trees showed no significant difference: Supplementary Table S3), and the respective classifications of *Kutjamarcoot* and *Crash* as either a stem perameloid^14^ or stem peramelids^3^,^12^, were likewise uncertain (Supplementary Figs S11 and S12). Similarly, the earliest dated peroryctine^1^,^4^, *cf. Peroryctes tedfordi*, was only intermittently nested within *Peroryctes* (Supplementary Fig. S10), a result that compromises existing molecular clock calibrations^16^. The affinity of *Perameles bowensis*^42^, which has also previously been used for dating constraints^1^,^4^, was unresolved^33^ (Supplementary Figs S8 and S9); however, this taxon manifests a posthypocristid-entoconid contact on its lower molars, which is distinctive for both *Perameles* and the Pleistocene species *P. sobbei*^38^ (topologically grouped with living *Perameles nasuta* and *P. gunnii*: Supplementary Figs S8 and S9), as well as the enigmatic Miocene–Pliocene genus *Ischnodon*. Notably, neither *Ischnodon* nor *Liyamayi* were recovered as ancestral bilbies^3^,^12^,^16^, and constraint tests on these placements were inconclusive (Supplementary Table S3). Such results corroborate the original taxonomic assessment of *Ischnodon*, which reported thylacomyid dental similarities but refrained from definitive classification^37^.

Our assessments of extant bandicoot morphology were consistent with DNA^1^,^4^ in returning the xeric-adapted *Macrotis lagotis* as the most divergent living peramelemorphian (Supplementary Fig. S13). On the other hand, alternate grouping of the extinct arid-zone chaeropodid *Chaeropus* within Peramelinae (Supplementary Fig. S14) suggests that either extensive dental/osteological convergence^3^, or incomplete characterization of its scant mitochondrial sequence data^43^ confound its relationships. Interestingly, inclusion of the extinct desert-adapted thylacomyid, *Macrotis leucura*, promoted extensive topological degradation (Supplementary Fig. S15). This might be due to its curious ‘peramelid-like’ dental attributes (see matrix scores in Supplementary Data), which could again denote either homoplasy, or the retention of ancestral perameloid states.

Trees generated by the concatenated dataset of morphology + DNA were identical to those produced by DNA alone^1^, but with amplified support values for weak nodes demonstrating overall signal congruence. This was most notable at the nodes excluding *Macrotis lagotis* from Peramelidae, and positioning of the Seram bandicoot *Rhynchomeles prattorum* within *Echymipera* (Supplementary Fig. S16). Successive deletion of molecular information for major clades^44^ pinpointed residual morphological conflict over a paraphyletic Peroryctinae + Echymiperinae, and repositioning of Chaeropodidae within Peramelinae (Supplementary Table S4). This concurs with previous studies^14^,^15^,^16^,^17^,^45^, which have placed *Macrotis* and *Chaeropus* outside of Peramelidae using a molecular backbone, but not with morphology on its own. As expected, the introduction of fossils completely degraded node support (Supplementary Fig. S17), and revealed long-branch effects in the clumped redistribution of taxa (Supplementary Fig. S18). Sequential deletion of highly homoplastic dental-dependant terminals did improve these results (Supplementary Figs S19 and S20), but still failed to yield stable positioning of fossils, perhaps because they integrate insufficient cranial-postcranial skeletal data to accurately discriminate relationships.

#### Divergence times and ancestral areas

We utilized a DNA dataset with expanded outgroup sampling of diprotodontian, notoryctemorphan and dasyuromorphian taxa to correlate the timeframes and settings for peramelemorphian intra-clade divergences. Alternative fossil constraints (Supplementary Table S5) and Bayesian random local clocks^46^ (Supplementary Fig. S21) were also implemented to assess possible sources of overestimation^16^. Despite these tests, our analyses demonstrated an unequivocal origination of the crown bandicoot total-group during the mid-Paleocene around 60 Ma (Table 1; Supplementary Tables S6–S8; Supplementary Figs S22–S30). This corroborates the discovery of possible stem peramelemorphian fossils from the early Eocene^3^,^9^,^47^, but massively predates previous molecular estimates^1^,^4^ by up to 40 Ma. In accordance, diversifications amongst chaeropodid, thylacomyid and peramelid family-level clades seem to have commenced in the middle Eocene to Oligocene (~40–30 Ma). These epochs coincide with the tectonic isolation of Australia and instigation of the circum-Antarctic current, which propagated seasonally cool-dry climates and the spread of sclerophyllous vegetation^48^. Compellingly, our S-DIVA/Bayesian Binary MCMC ancestral area optimisations onto morphological (Supplementary Figs S31 and S32), total evidence (Supplementary Fig. S33), and DNA trees (Supplementary Fig. S34) decisively correlated the basal peramelemorphian split with dispersal into open habitats and an early occupation of xeric ecosystems (Supplementary Tables S9–S12). Unanimous inference of a post early–middle Miocene (after ~20 Ma) rainforest-woodland radiation amongst peroryctines and echymiperines likewise coincides with uplift of the New Guinean landmass and onset “greenhouse” climates^49^, which propagated higher rainfall and coastal/riparian vegetation^50^. In contrast, our analyses failed to pinpoint an emergent habitat for peramelines. We attribute this to their rapid expansion into openly vegetated environments^1^,^3^,^51^, compounded by methodological dependence of our probability matrices upon predefined species distribution codes. These are particularly sensitive to highly dispersive organisms, as well as significant area changes through time^52^. In our case this included the pronounced middle–late Miocene (after ~16 Ma) resurgence of cool-dry climates, and Pliocene predominance of mosaic vegetation, especially incorporating intra-continental grasslands which proliferated across Australia during this interval^50^.

## Discussion

The ‘complete’ centrocristae delimiting broad ectolophs, and extreme metaconular reduction on the M1–3 of *Lemdubuoryctes* are virtually identical to the conditions found in the most ancient fossil bandicoots *Yarala kida*^21^ and *Bulungu muirheadae*^16^. As shown here, these unexpected state expressions have radical implications for bandicoot phylogeny in placing *Lemdubuoryctes* as an exceptionally late-surviving stem-grade peramelemorphian. Moreover, the presence of both residual centrocrista and metaconules on the upper molars of the early–middle Miocene *Bulungu*^15^,^16^ and *Galadi*^17^,^18^, as well as the early Pliocene *cf. Peroryctes tedfordi*^33^, and extant species of *Peroryctes* and *Echymipera*^34^ shows that these symplesiomorphies were persistent throughout bandicoot evolution, and could represent examples of repeated convergent atavism. Although postulated^3^,^16^,^18^,^27^, such rampant homoplasy has never previously been demonstrated within the fundamental discriminative features of the peramelomorphian dentition. Equally as significant is our topological nesting of *Bulungu campbelli* amongst living perameloids^3^, which implies a corresponding appearance of advanced dental traits within the stratigraphically earliest bandicoot lineages. *Bulungu campbelli* is a late Oligocene species (Etadunna Formation Zone C: 24.6−24.1 Ma^53^) that approximates the oldest known fossil peramelemorphian taxon *B. muirheadae* (Etadunna Formation Zone B: 24.9−24.6 Ma^53^). *Bulungu campbelli* is also important because it predates what is usually regarded as the most plesiomorphic bandicoot *Yarala kida* (Wipajiri Formation equivalent: <24 Ma^53^,^54^). Our new phylogenetic arrangement therefore pushes back the feasible minimum age for crown Peramelemorphia by more than 20 Ma (previous molecular clock constraints have used an upper limit of 4.36 Ma based on *cf. Peroryctes tedfordi*^1^,^3^,^22^,^23^,^24^), and also infers a pectinate pattern of past higher-level diversity (Fig. 1) that challenges the traditional yaraloid versus perameloid clade/time-division model^3^,^9^,^10^,^19^,^20^,^21^,^27^.

The survival of *Lemdubuoryctes* on what is today a rainforest prevalent^28^ island refuge, seemingly accords with the most ancient peramelemorphian habitats^12^,^13^,^16^,^17^,^18^. However, the stratigraphical horizons containing *Lemdubuoryctes* fossils date from prior to inundation of the Torresian Plain after the Last Glacial Maximum^28^,^29^,^30^,^31^. At this time, the Aru Islands were a limestone plateau surrounded by open savannah plains with dense riparian forest restricted to topographic lows along fault-controlled ‘sungai’ channels^28^,^29^,^30^,^31^. The fossil bandicoot species from these settings are dominated by both *Lemdubuoryctes* and *Isoodon macrourus*, the latter being an extant grassland–open woodland inhabitant. On the other hand, *Echymipera rufescens* which presently occupies lowland rainforests on the Aru Islands^29^ is comparatively rare, and *E. kalubu* which typifies rainforests and anthropogenic grasslands in high rainfall areas, has been tentatively identified from a few teeth but these post-date the Holocene marine transgression^29^. The numerical abundance of *Lemdubuoryctes* at Liang Lemdubu and Liang Nabulei Lisa, coupled with palynomorph evidence^28^, and its associated open savannah–moist forest vertebrate assemblage^29^, could therefore suggest a preference for heterogenous habitats. This pointedly compliments zoogeographic correlations of the late Pleistocene Aru Islands with mosaic ecotones in northern Australia and the Trans-Fly region of southern New Guinea^28^,^29^, as well as the reconstructed palaeoenvironments ascribed to other plesiomorphic Pliocene–Pleistocene bandicoots^3^,^27^,^55^,^56^. In stark contrast, early to middle Miocene peramelemorphians are usually considered to have been rainforest specialists, a conclusion based on associated mammalian faunas^57^, and most tellingly, their archaic craniodental morphologies^12^,^13^,^16^,^17^,^18^. This key premise underlies the Miocene ‘bottleneck’ hypothesis, under which environmentally constrained stem taxa were replaced by crown perameloid lineages^9^,^10^,^16^,^19^,^20^,^21^,^27^ that more successfully adjusted to changing climates^4^,^22^,^23^ and dietary competition with emergent dasyurid marsupial carnivores and rodents migrating from Asia^3^,^15^,^17^,^18^,^19^. Conversely, the discovery of *Lemdubuoryctes* reveals that these phenomena in fact did not prevent the survival of archetypal peramelemorphians through to the terminal Pleistocene–Holocene. In addition, our demonstration of profound antiquity for modern desert-living bandicoot lineages (a result unaffected by alternative constraint parameters or variation in substitution rate^16^: Supplementary Tables S6–S8; Supplementary Figs S21–S30), indicates that increasing aridity during the late Neogene likely did not initiate the genesis of crown Perameloidea, although it probably assisted in perameline intra-clade habitat expansion and localized speciation events. The undeniable rarity of definitive crown perameloid fossils in pre-Pliocene sediments might therefore be explained by sampling biases and/or incompletely documented collections^3^,^27^,^58^,^59^, as well as ecological underrepresentation^60^. Indeed, our tree-based ancestral area optimisations (Supplementary Tables S9–S12; Supplementary Figs S31–S34) infer that the seminal radiation of modern bandicoots accompanied widespread australidelphian niche dispersals into drier mosaic settings, perhaps such as mallee (*Eucalyptus*) woodlands that spread through Central Australia from the late Oligocene^50^,^61^. The scarcity of crown bandicoot antecedents in intensively studied fluvial or karstic contexts^54^,^57^,^58^ with higher preservation potential thus becomes understandable, as does the ecologically disjunct DNA-based phylogeny of living peramelemorphians. This now clearly captures one of the most deeply divergent radiations of xeric-adapted marsupials^1^,^3^,^4^,^22^,^23^,^24^,^32^, and reinforces a biota-wide exaptive response to late Neogene aridity^62^, including diversification amongst clades that had already maintained substantial habitat disparity for many millions of years.

## Methods

### Dataset construction

Peramelemorphian morphological phylogenies have suffered from persistently inadequate resolution^12^,^13^,^14^,^15^,^16^,^17^,^18^ prompting weighting of dental data via homologous sets^63^ and incremental qualitative subdivisions^64^. We therefore compiled a de novo matrix that emphasized partition sampling^65^ across 93 cranial-dental and postcranial characters assembled for demonstrable extant outgroup dasyurid/didelphid marsupials^66^ and 36 ingroup bandicoot species including 13 fossil taxa: *Bulungu campbelli* and *Yarala kida* (late Oligocene), *Bulungu palara*, *Kutjamarcoot brevirostrum*, *Madju variae* (late Oligocene−early Miocene), *Crash bandicoot*, *Galadi amplus*, *Galadi speciosus*, *Kutjamarcoot brevirostrum*, *Liamaya dayi* and *Yarala burchfieldi* (middle Miocene), *Ischnodon australis*, cf. *Peroryctes tedfordi* and *Perameles bowensis* (early Pliocene), *P. sobbei* (late Pleistocene), and *Lemdubuoryctes aruensis* (late Pleistocene−Holocene). Scores were taken from multiple (where available) original specimens (all crown taxa, *G. speciosus*, *Y. burchfieldi*, *I. australis*, *P. sobbei*, *L. aruensis*), high-definition casts (*B. campbelli*), and/or reference to relevant published works (*Y. kida*, *B. palara*, *K. brevirostrum*, *M. variae*, *C. bandicoot*, *G. amplus*, *L. dayi*; the type remains of cf. *P. tedfordi* and *P. bowensis* could not be located by their housing institutions). State definitions were either redrafted from earlier studies (1−63)^11^,^12^,^13^,^14^,^15^,^16^,^17^,^18^,^25^,^27^,^34^,^37^,^67^, or determined exclusively for this phylogeny (64−93). A complete character list with annotations describing modified state definitions, redundancy and/or rescoring, the morphological data matrix, and a catalogue of examined specimens are provided in the Supplementary Data.

The concatenated series of 9977 DNA sequence nucleotides representing five nuclear (ApoB, BRCA1, IRBP, RAG1, vWF) and three mitochondrial genes (12 S rRNA, cytochrome *b* and the 3′ portion of 16 S rRNA) was used to: (1) produce a backbone tree (Supplementary Fig. S7) that determined the best-supported position of fossils relative to the living species topology; and (2) compute a combined (non-weighted) total evidence analysis that examined effects of morphological data on molecular nodes. Laboratory procedures, DNA sequence derivation/alignment, and model testing of separate gene/codon regions were described in Westerman *et al.*^1^,^68^.

### Phylogenetic analysis

We implemented a six-stage strategy to manage the detrimental effects of incomplete fossils and characters^69^. (1) Initial selection of operational taxonomic units [OTUs] specifically targeted extant taxa with overlapping coverage of morphological and DNA sampling^70^. Fossil OTUs included only the most complete genus-level exemplars for branching lineages (*Bulungu*^16^, *Galadi*^18^, *Madju*^13^) as well as those species critical for molecular clock calibrations (*Yarala kida*, *cf. Peroryctes tedfordi*, *Perameles bowensis*)^1^,^4^ or uncontested crown clade referral (*Perameles sobbei*)^3^,^38^,^45^. (2) Excessively incomplete DNA characters were removed in morphology-only analyses to examine the effects of extinct taxa with numerous missing entries^71^. A manual screen for redundant taxa (safe taxonomic reduction^40^) identified all OTUs that degraded strict consensus resolution and pinpointed instability caused by missing data versus character conflict^72^. (3) The ‘amb-’ option was implemented during all PAUP* v4.0b10^73^ parsimony searches to eliminate ambiguous zero length branches^74^. (4) A posteriori screening of wildcard taxa produced a strict reduced consensus profile based on the semi-strict Adams consensus (where wildcards do not obscure adequately supported nodes^40^,^41^) and assessments of relative character support at affected nodes^74^. (5) Bootstrapping and branch (Bremer) decay indices were alternately employed with and without wildcard exclusion to test the impact of mobile OTUs upon support measures. (6) Sequential exclusion of fossils incorporating numerous missing entries for cranial and postcranial characters was used to assess the effects of sub-sampling and long-branch attraction^74^ within the total evidence framework^70^.

Parsimony trees and bootstrap frequencies (1000 repetitions) were computed using heuristic searches with TBR (tree-bisection-reconnection) branch swapping and 100 random-addition replicates. The molecular scaffold enforced monophyly for clades receiving ≥70% partitioned maximum likelihood bootstrap, and ≥0.95 Bayesian posterior probability support. Bremer values were calculated for unconstrained morphological data with *TNT* v1.1^75^, which also cross-referenced MP topologies via a ‘New Technology Search’ with sectorial searches, drift, and tree fusing enabled. Results were then processed using a ‘Traditional Search’ option with TBR. DELTRAN character state optimisation was preferentially employed for tree construction, but unequivocal synapomorphies were shared by both DELTRAN and ACCTRAN outputs (see Supplementary Data).

Bayesian analyses used *MrBayes* ver.3.2^76^ with the obligate standard discrete model (*Mkv*)^77^ for morphology. Because this assumes equal change between all character states, we set the rates variation parameter to ‘gamma’ (with coding to ‘variable’), thereby introducing heterogeneity, and also tested data partitioning via cranial/mandibular (1–6, 32–55, 59, 65), dental (7–31, 56–58, 60–64), and postcranial (66–93) character sets^65^. Gene partitions followed Westerman *et al.*^1^,^68^. Two simultaneous runs and four Markov Chains (one cold and three heated using default heating values) were applied for 5 × 10^6^ generations with default settings and the burn-in fraction set at 0.25.

### Molecular clocks

Time-trees were generated in *BEAST2*^78^ with uncorrelated relaxed lognormal clocks. A Bayesian random local clock model was also used to test for overestimation imposed by punctuated shifts in substitution rate^46^. We used traditional node dating to assess contested^16^ ingroup constraints for Peramelemorphia, Chaeropodidae, Thylacomyidae, Peroryctinae + Echymiperinae, Peramelinae and *Isoodon* + *Perameles*. Minimum and maximum fossil calibrations (Supplementary Table S5) were compiled according to best practice protocols^79^. Analyses incorporated a birth-death model and normal priors imposed on soft bounds with 95% distribution between the minimum and maximum. MCMC analyses were run for 50 × 10^6^ generations with a 25% burn-in for tree summaries. Runs were terminated when ESS values reached >200 for all estimated parameters.

### Ancestral areas

Because the precise topological placement and habitat preferences of fossil peremelemorphians are uncertain, we used a non-ultrametric Bayesian DNA tree sub-sample of extant taxa, and alternative S-DIVA (tree dataset) versus Bayesian Binary MCMC (condensed tree accommodating topological uncertainty) approaches implemented in *RASP* ver. 3.2^80^ to infer ancestral habitat dispersal patterns. Four Markov Chains (default heating values) were run twice over 5 × 10^6^ generations with sampling frequency and burn-in fixed at 500. Among-site rate variation was set to ‘gamma’ and state frequencies utilized a ‘fixed (JC)’ model. Habitat codings were generalized to accommodate for non-exclusivity, but broadly adhered to defined vegetation units^50^: A = rainforest; B = woodland (referring primarily to sclerophyll forests); C = shrubland (including ‘open’ and xeromorphic vegetation); and D = arid/semi-arid vegetation and desert.

### Nomenclatural acts

ZooBank (http://zoobank.org/) registration of nomenclatural acts in this published work can be accessed using the Life Science Identifier: urn:lsid:zoobank.org:pub:7AC0A046-A507-4AA2-933D-F1A36132F2FD.

## Additional Information

**How to cite this article**: Kear, B. P. *et al.* Bandicoot fossils and DNA elucidate lineage antiquity amongst xeric-adapted Australasian marsupials. *Sci. Rep.* **6**, 37537; doi: 10.1038/srep37537 (2016).

**Publisher's note:** Springer Nature remains neutral with regard to jurisdictional claims in published maps and institutional affiliations.

## Supplementary Material

Supplementary Information

## Figures and Tables

**Table 1:** Molecular divergence date estimates for peramelemorphian clades (million years BP).

Node	Divergence estimates	
	No constraints	Ingroup constraints
Dasyuromorphia + Peramelemorphia	60.08 (54.61–65.7)	60.34 (54.74–65.79)
*Chaeropus* v *Macrotis* + Peramelidae	37.87 (29.78–46.05)	38.06 (30.14–46.32)
*Macrotis* v Peramelidae	31.45 (25.38–38.03)	31.55 (25.76–38.24)
Peramelinae v Peroryctinae + Echymiperinae	20.24 (16.41–24.23)	20.49 (16.77–24.43)
Peroryctinae v Echymiperinae	17.37 (13.96–21.15)	17.55 (14.08–21.08)
*Peroryctes broadbenti* v *P. raffrayana*	8.01 (5.12–11.4)	8.08 (5.08–11.62)
*Rhynchomeles* + *Echymipera* v *Microperoryctes*	12.08 (9.53–14.97)	12.21 (9.62–15.14)
*Rhynchomeles* v *Echymipera*	10.6 (7.81–13.62)	10.7 (7.92–13.8)
*E. clara* v *E. kalubu* + *E. rufescens*	9.76 (7.36–12.51)	9.85 (7.36–12.59)
*E. kalubu* v *E. rufescens*	6.34 (4.22–8.73)	6.4 (4.18–8.85)
*Microperoryctes* sp. (Tembagapura) v *M. ornata* + *M. longicauda* (Sol) + *M. papuensis*	8.11 (5.83–10.58)	8.18 (5.84–10.74)
*M. ornata* + *M. longicauda* (Sol) v *M. papuensis*	4.95 (3.29–6.9)	5.01 (3.35–6.95)
*M. ornata* v *M. longicauda* (Sol)	2.16 (1.16–3.4)	2.17 (1.15–3.42)
*Isoodon* v *Perameles*	13.2 (10.34–16.5)	13.73 (10.84–16.91)
*I. auratus* v *I. macrourus* + *I. obesulus*	5.18 (3.42–7.2)	5.25 (3.46–7.3)
*I. macrourus* v *I. obesulus*	3.27 (1.94–4.93)	3.31 (1.94–4.94)
*Perameles bougainville* + *P. eremiana* v *P. gunnii* + *P. nasuta*	11.38 (8.52–14.44)	11.83 (9–14.8)
*P. bougainville* v *P. eremiana*	8.5 (5.07–12.15)	8.81 (5.27–12.51)
*P. gunnii* v *P. nasuta*	4.71 (2.81–7.04)	4.77 (2.82–7.12)

Results based on partitioned data with revised fossil constraints (minimum–maximum): Peramelemorphia (24.6–54.6); Chaeropodidae (2.47–24.6); Thylacomyidae (11.608–24.6); Peroryctinae + Echymiperinae (4.36–24.6); Peramelinae (14.64–24.6); *Perameles* + *Isoodon* (0.04515–14.82). Outcomes of alternative constraint tests are shown in Supplementary Tables S6–S8.
