# Supplementary material for: Bandicoot fossils and DNA elucidate lineage antiquity amongst xeric-adapted Australasian marsupials
Source: Sci Rep. 2016 Nov 24;6:37537. doi: 10.1038/srep37537 (PMC5121598; doi:10.1038/srep37537)
Supplement: Supplementary Information [file srep37537-s1.doc]

Supplementary Information for

Bandicoot fossils and DNA elucidate lineage antiquity amongst xeric-adapted Australasian marsupials

Benjamin P. Kear^1^, Ken P. Aplin^2^ & Michael Westerman^3^

^1^Museum of Evolution, Uppsala University, Norbyvägen 16, SE-752 36 Uppsala, Sweden.

^2^Division of Mammals, National Museum of Natural History, Smithsonian Institution, P.O. Box. 37012, Washington, DC, 20013-7012, USA.

^3^Department of Ecology, Environment and Evolution, La Trobe University, Melbourne, Victoria 3086, Australia.

Correspondence and requests for materials should be addressed to B.P.K. (email: benjamin.kear@em.uu.se)

**Supplementary Tables**

**Supplementary Table S1. Upper tooth measurements (mm) of *Lemdubuoryctes aruensis*.** Holotype and referred maxillae: l = maximum length; w = maximum width; *holotype.

| **Specimen** | **lP1** | **wP1** | **lP2** | **wP2** | **lP3** | **wP3** | **lM1** | **wM1** | **lM2** | **wM2** | **lM3** | **wM3** | **lM4** | **wM4** |
| --- | --- | --- | --- | --- | --- | --- | --- | --- | --- | --- | --- | --- | --- | --- |
| *WAM 14.9.6 | - | - | - | - | 4.93 | 3.07 | 5.13 | 3.12 | 4.43 | 3.68 | - | - | - | - |
| WAM 14.9.9 | - | - | - | - | 3.05 | 1.68 | 4.46 | 2.95 | 4.15 | 3.48 | 4.24 | 3.92 | 2.84 | 3.58 |
| WAM 14.9.11 | 3.33 | 1.32 | 4.25 | 1.75 | - | - | - | - | - | - | - | - | - | - |

**Supplementary Table S2. Upper tooth measurements (mm) of *Lemdubuoryctes aruensis*.** Referred mandibles: l = maximum length; w = maximum width; ^i^incomplete.

| **Specimen** | **lp1** | **wp1** | **lp2** | **wp2** | **lp3** | **wp3** | **lm1** | **wm1** | **lm2** | **wm2** | **lm3** | **wm3** | **lm4** | **wm4** |
| --- | --- | --- | --- | --- | --- | --- | --- | --- | --- | --- | --- | --- | --- | --- |
| WAM 14.9.1 | 4.05 | 1.42 | 4.7 | 1.79 | 5.9 | 2.53 | - | - | 4.36 | 2.48 | 4.32 | 2.31 | - | 2.14 |
| WAM 14.9.2 | - | - | 4.1 | 1.9 | 5.62 | 2.53 | - | - | 4.33 | 2.58 | 4.37 | 2.4 | - | - |
| WAM 14.9.3 | - | - | - | - | 4.1 | 1.78 | 3.99 | 2.2 | 4.19 | 2.38 | 4.19 | 2.3 | 4.17 | 2.1 |
| WAM 14.9.4 | - | - | - | - | - | - | - | - | 4.25 | 2.24 | 2.37 | - | - | - |
| WAM 14.9.5 | - | - | - | - | 6.17 | 2.78 | - | - | - | - | - | - | - | - |
| WAM 14.9.7 | - | - | - | - | - | - | 4.33 | 2.42 | 4.32 | 2.59 | 4.33 | 2.42 | 4.32 | 2.14 |
| WAM 14.9.8 | 3.65 | 1.27 | 4.58^i^ | 1.73 | - | - | - | - | - | - | - | - | - | - |
| WAM 14.9.10 | - | - | - | - | - | - | - | - | - | - | 4.51 | 2.46 | - | - |
| WAM 14.9.12 | - | - | - | - | - | - | 4.32 | 2.14 | - | - | - | - | - | - |

**Supplementary Table S3. Parsimony (non-parametric Templeton test) and Bayes factor (1,000,000 generations) comparisons of topological relationships using the morphological data set of Peramelemorphia.** Bayesian log unit values represent hard/negative constraints for each specified partition.

| **Constraint** | **Parsimony (excluding other fossils)** | **Harmonic Mean Estimate** | **Stepping-Stone Sampling** |
| --- | --- | --- | --- |
| (Yaraloidea / *Yarala burchfieldi* + *Y. kida*) | 1/2 best trees | –814.53/-809.48 | –878.54/–880.29 |
| (*Bulungu palara* + *B. campbelli*) | +2 steps; *p* > 0.1573 | –812.05/–805.58 | –875.44/–875.32 |
| (*Galadi speciosus* + *G. amplus*) | 16/16 best trees | –819.62/–823.36 | –890.41/–891.11 |
| (*Madju variae* + Peroryctinae + Echymiperinae) | 2/8 best trees | –793.3/–794.14 | –846.13/–863.01 |
| (*Kutjamarcoot brevirostrum* + Thylacomyidae) | +1 step; *p* > 0.3173 | –783.3/–784.94 | –843.50/–846.75 |
| (*Liyamayi dayi* + Thylacomyidae) | Equal steps; *p* > 0.0047 | –775.24/–773.47 | –836.17/–839.05 |
| (*Ischnodon australis* + Thylacomyidae) | +1 step; *p* > 0.3173 | –781.82/–778.35 | –844.02/–845.92 |
| (*Crash bandicoot* + Peramelinae) | 15/21 best trees | –778.52/–780.53 | –836.31/–843.72 |
| (*cf. Peroryctes tedfordi* + Peroryctinae) | Equal steps; *p* > 0.9604 | –780.46/–786.47 | –847.35/–845.13 |
| (*Perameles bowensis* + Peramelinae) | 6/54 best trees | –784.86/–777.39 | –838.58/–841.47 |
| (*Perameles sobbei* + *Perameles gunni* + *Perameles nasuta*) | Equal steps; *p* = 0.6831 | –775.85/–777.26 | –839.5/–842.01 |
| (Crown Perameloidea backbone) | +77 steps; *p* < 0.0001 | –783.63/–780.4 | –842.46/–843 |

Constraint clades. Crown Perameloidea *–* (Chaeropus_ecaudatus + Echymipera_clara + Echymipera_kalubu + Echymipera_rufescens + Isoodon_auratus + Isoodon_macrourus + Isoodon_obesulus + Macrotis_lagotis + Macrotis_leucura + Microperoryctes_longicauda_Sol + Microperoryctes_ornata + Microperoryctes_papuensis + Microperoryctes_Tembagapura + Perameles_bougainville + Perameles_eremiana + Perameles_gunnii + Perameles_nasuta + Peroryctes_broadbenti + Peroryctes_raffrayana + Rhynchomeles_prattorum); Thylacomyidae – (Macrotis_lagotis + Macrotis_leucura); Peroryctinae – (Peroryctes_broadbenti + Peroryctes_raffrayana); Echymiperinae – (Echymipera_clara + Echymipera_kalubu + Echymipera_rufescens + Microperoryctes_longicauda_Sol + Microperoryctes_ornata + Microperoryctes_papuensis + Microperoryctes_Tembagapura + Rhynchomeles_prattorum); Peramelinae – (Isoodon_auratus + Isoodon_macrourus + Isoodon_obesulus + Perameles_bougainville + Perameles_eremiana + Perameles_gunnii + Perameles_nasuta).

**Supplementary Table S4. Results from parsimony and Bayesian analyses of data sets sequentially excluding DNA sequence information^44^ for peramelemorphian family and subfamily-level clades.** Bracketed numbers represent parsimony bootstrap percentages/Bayesian posterior probabilities (see main text for parameters).

| **Clade** | **Monophyletic** | **Parsimony Topology** | **Bayes Topology** |
| --- | --- | --- | --- |
| Chaeropodidae | Monotypic | Nested within Peramelinae (99); Chaeropodidae + *Perameles* (<50) | Nested within Peramelinae (1); Chaeropodidae + *Isoodon* (0.67) |
| Thylacomyidae | Monotypic | Basal-most peremelemorphian (<50) | Nested within Peramelidae (0.77); Peramelinae (0.7); Thylacomyidae + *Perameles* (0.51) |
| Peramelidae | No/Yes | Paraphyletic with *Microperoryctes* (51); Thylacomyidae + Chaeropididae + Peramelinae (84); Chaeropididae + Peramelinae (60) | Monophyletic with paraphyletic Peramelinae (0.75); paraphyletic Peroryctinae + Echymiperinae (0.99) |
| Peramelinae | Yes | Thylacomyidae + Chaeropididae + Peramelinae (68); Chaeropididae + Peramelinae (52) | Chaeropididae + Peramelinae (0.88) |
| Peroryctinae | Yes/No | Nested within Echymiperinae (68) | Polyphyetic within Echymiperinae (1) |
| Echymiperinae | No/Yes | Basal polytomy with *Rhynchomeles* +*Microperoryctes* (<50); *Microperoryctes* spp. (54) | Echymiperinae (0.45) with Rhynchomeles +*Microperoryctes* (0.66) |

**Supplementary Table S5. Revised fossil constraints for molecular dating analyses.** Clade parameters were modified from Westerman *et al*.^1^: PB = phylogenetic bracketing; SB = stratigraphical bracketing. *An alternative early Miocene SB (23.03) was also tested because phylogenetic placement of *Barinya* spp. within Dasyuridae is uncertain^81,82^.

| **Clade** | **Min. (Ma)** | **Max. (Ma)** | **Oldest Fossil** | **PB** | **SB** | **Reference for Min.** | **Reference for Max.** |
| --- | --- | --- | --- | --- | --- | --- | --- |
| Australasian marsupials | 54.6 | 65.8 | Murgon ‘stem perameloid’ | *Peradectes* (Puercan) | - | Godthelp *et al*.^47,83^; Meredith *et al*.^24^; Archer *et al*.^27^ | Beck *et al*.^84^; Westerman *et al*.^1^ |
| Australiadelphia + Didelphimorpha | 66 | 72.1 | *Peradectes* (Puercan); *Swaindelphys* (mid-late Torrejonian); *Mimoperadectes* (early Wasatchian) | - | Maastrichtian | Horovitz *et al*.^85^; Williamson *et al*.^86^ | Case *et al*.^87^ |
| Dasyuridae + Myrmecobiidae | *17.85 | 54.6 | *Barinya wangala* | Murgon ‘stem perameloid’ | - | Wroe^88^; Woodhead *et al*.^89^ | Westerman *et al*.^68^ |
| *Antechinus* + *Phascogale* | 4.36 | *17.85 | *Antechinus* sp. | *Barinya wangala* | - | Turnbull *et al*.^12^ | Westerman *et al*.^68^; Wroe^88^; Woodhead *et al*.^89^ |
| *Dasyurus* + *Phascolosorex* | 4.36 | *17.85 | cf. *Dasyurus* sp. | *Barinya wangala* | - | Turnbull *et al*.^12^ | Westerman *et al*.^68^; Wroe^88^; Woodhead *et al*.^89^ |
| Peramelemorphia | 24.6 | 54.6 | *Bulungu muirheadae* | Murgon ‘stem perameloid’ | - | Travoullion *et al*.^17^; Megirian *et al*.^53^ | Westerman *et al*.^1^ |
| Peroryctinae + Echymiperinae | 4.36 | 24.6 | cf. *Peroryctes tedfordi* | *Bulungu campbelli* | - | Turnbull *et al*.^12^ | Travoullion *et al*.^17^; Megirian *et al*.^53^ |
| Peramelinae | 14.64 | 24.6 | *Crash bandicoot* | *Bulungu campbelli* | - | Travoullion *et al*.^13^; Woodhead *et al*.^89^ | Travoullion *et al*.^17^; Megirian *et al*.^53^ |
| *Perameles + Isoodon* | 0.04515 | 14.82 | *Perameles sobbei* | *Crash bandicoot* | - | Price^38^ | Travoullion *et al*.^13^; Woodhead *et al*.^89^ |
| Thylacomyidae | 11.608 | 24.6 | *Liyamayi dayi* | *Bulungu campbelli* | Middle Miocene | Travoullion *et al*.^13^ | Travoullion *et al*.^17^; Megirian *et al*.^53^ |
| Chaeropodidae | 2.47 | 24.6 | *Chaeropus baynesi* | *Bulungu campbelli* |  | Travoullion *et al*.^60^ | Travoullion *et al*.^17^; Megirian *et al*.^53^ |
| Vombatiformes | 24.9 | 54.6 | *Perikoala robusta* | Murgon ‘stem perameloid’ | - | Woodburne *et al*.^58^; Megirian *et al*.^53^ | Meredith *et al*.^90^ |
| Petauridae + Pseudocheiridae | 24.9 | 54.6 | *Paljara* sp. A | Murgon ‘stem perameloid’ | - | Woodburne *et al*.^58^; Megirian *et al*.^54^ | Meredith *et al*.^91^ |
| Phalangeridae + Burramyidae | 24.6 | 54.6 | *Eocuscus sarastamppi* | Murgon ‘stem perameloid’ | - | Case *et al*.^92^; Megirian *et al*.^53^ | Meredith *et al*.^90^ |
| Macropodidae + Potoroidae | 24.1 | 54.6 | *Bulungamaya* | Murgon ‘stem perameloid’ | - | Kear *et al*.^93^; Kear & Pledge^94^; Black *et al*.^95^; Megirian *et al*.^53^ | Meredith *et al*.^90^ |

**Supplementary Table S6. Results of alternative revised constraints for Peramelemorphia and its constituent ingroup clades.** Minimum–maximum values are shown in Supplementary Table S5. Divergence date estimates (million years BP) are graphically represented in Supplementary Figs S22–S24. Divergence estimates based on Peremelemorphia only excluded all ingroup node calibrations except for *Perameles + Isoodon*.

| **Node** | **Divergence Estimates** | | |
| --- | --- | --- | --- |
|  | **Peramelemorpha** | **Peror. + Echym.**  **/ Peramelinae** | **Peror. + Echym.**  **/ Peram. / Thylaco.** |
| Dasyuromorphia v Peramelemorphia | 60.08 (54.61–65.7) | 60.38 (55.07–66.15) | 60.37 (54.95–66.01) |
| *Chaeropus* v *Macrotis* + Peramelidae | 37.87 (29.78–46.05) | 38.05 (29.88–46.27) | 38.14 (29.82–46.22) |
| *Macrotis* v Peramelidae | 31.45 (25.38–38.03) | 31.56 (25.64–38.41) | 31.75 (25.63–38.2) |
| Peramelinae v Peroryctinae + Echymiperinae | 20.24 (16.41–24.23) | 20.56 (16.77–24.54) | 20.52 (16.92–24.66) |
| Peroryctinae v Echymiperinae | 17.37 (13.96–21.15) | 17.61 (14.26–21.4) | 17.57 (14.12–21.26) |
| *Peroryctes broadbenti* v *P. raffrayana* | 8.01 (5.12–11.4) | 8.01 (4.93–11.49) | 8.13 (5.1–11.48) |
| *Rhynchomeles + Echymipera* v *Microperoryctes* | 12.08 (9.53–14.97) | 12.31 (9.74–15.32) | 12.22 (9.65–15.05) |
| *Rhynchomeles* v *Echymipera* | 10.6 (7.81–13.62) | 10.82 (8.06–13.97) | 10.73 (7.91–13.72) |
| *E. clara* v *E. kalubu* + *E. rufescens* | 9.76 (7.36–12.51) | 9.98 (7.51–12.76) | 9.87(7.3–12.5) |
| *E. kalubu* v *E. rufescens* | 6.34 (4.22–8.73) | 6.47 (4.23–8.97) | 6.42 (4.23–8.92) |
| *Microperoryctes* sp. (Tembagapura) v *M. ornata + M. longicauda* (Sol) *+ M. papuensis* | 8.11 (5.83–10.58) | 8.26 (5.96–10.79) | 8.15 (5.88–10.7) |
| *M. ornata + M. longicauda* (Sol) v *M. papuensis* | 4.95 (3.29–6.9) | 5.05 (3.39–7.01) | 5.02 (3.34–7.01) |
| *M. ornata* v *M. longicauda* (Sol) | 2.16 (1.16–3.4) | 2.18 (1.2–3.45) | 2.19 (1.18–3.43) |
| *Isoodon* v *Perameles* | 13.2 (10.34–16.5) | 13.72 (10.85–17.08) | 13.71 (10.87–16.98) |
| *I. auratus* v *I. macrourus + I. obesulus* | 5.18 (3.42–7.2) | 5.22 (3.48–7.29) | 5.32 (3.55–7.44) |
| *I. macrourus* v *I. obesulus* | 3.27 (1.94–4.93) | 3.29 (1.93–4.89) | 3.35 (2–5.03) |
| *Perameles bougainville* + *P. eremiana* v *P. gunnii + P. nasuta* | 11.38 (8.52–14.44) | 11.83 (9.1–15.07) | 11.83 (9.05–14.93) |
| *P. bougainville* v *P. eremiana* | 8.5 (5.07–12.15) | 8.81 (5.22–12.53) | 8.83 (5.23–12.59) |
| *P. gunnii* v *P. nasuta* | 4.71 (2.81–7.04) | 4.85 (2.87–7.17) | 4.83 (2.88–7.18) |

Echym., Echymiperinae; Peram., Peramelinae; Peror., Peroryctinae; Thylaco., Thylacomyidae.

**Supplementary Table S7. Results of alternative revised constraints for peramelemorphian family/subfamily-level ingroup clades.** Minimum–maximum values are shown in Supplementary Table S5. Divergence date estimates (million years BP) are graphically represented in Supplementary Figs S25–S27.

| **Node** | **Divergence Estimates** | | |
| --- | --- | --- | --- |
|  | **Peror. + Echym.** | **Peramelinae** | **Thylaco.** |
| Dasyuromorphia v Peramelemorphia | 60 (54.45–65.6) | 60.37 (54.81–65.91) | 60.16 (54.56–65.58) |
| *Chaeropus* v *Macrotis* + Peramelidae | 37.9 (30.01–46.35) | 38.16 (30.38–46.63) | 37.62 (29.69–45.9) |
| *Macrotis* v Peramelidae | 31.48 (20.9–30.1) | 31.83 (25.56–38.29) | 31.24 (25.49–37.8) |
| Peramelinae v Peroryctinae + Echymiperinae | 20.22 (16.5–24.16) | 20.63 (16.93–24.87) | 20.2 (16.64–24.29) |
| Peroryctinae v Echymiperinae | 17.34 (14.1–21.06) | 17.7 (14.21–21.62) | 17.33 (13.89–20.91) |
| *Peroryctes broadbenti* v *P. raffrayana* | 8 (9.49–14.83) | 8.19 (5.06–11.66) | 7.95 (5.19–11.31) |
| *Rhynchomeles + Echymipera* v *Microperoryctes* | 12.08 (7.96–13.62) | 12.29 (9.68–15.21) | 12.11 (9.53–14.98) |
| *Rhynchomeles* v *Echymipera* | 10.6 (7.96–13.62) | 10.79 (8–13.92) | 10.62 (7.81–13.57) |
| *E. clara* v *E. kalubu* + *E. rufescens* | 9.77 (7.35–12.4) | 9.93 (7.4–12.67) | 9.81 (7.28–12.45) |
| *E. kalubu* v *E. rufescens* | 6.32 (4.14–8.63) | 6.44 (4.17–8.85) | 6.4 (4.3–8.83) |
| *Microperoryctes* sp. (Tembagapura) v *M. ornata + M. longicauda* (Sol) *+ M. papuensis* | 8.06 (5.81–10.64) | 8.22 (5.97–10.84) | 8.08 (5.78–10.59) |
| *M. ornata + M. longicauda* (Sol) v *M. papuensis* | 4.94 (3.24–6.91) | 5.03 (3.35–7.06) | 4.97 (3.27–6.93) |
| *M. ornata* v *M. longicauda* (Sol) | 2.13 (1.14–3.39) | 2.18 (1.18–3.4) | 2.16 (1.16–3.35) |
| *Isoodon* v *Perameles* | 13.21 (10.31–16.45) | 13.75 (10.75–16.96) | 13.17 (10.3–16.28) |
| *I. auratus* v *I. macrourus + I. obesulus* | 5.18 (3.4–7.33) | 5.28 (3.54–7.4) | 5.16 (3.41–7.2) |
| *I. macrourus* v *I. obesulus* | 3.26 (1.93–4.98) | 3.35 (2–5.03) | 3.27 (1.99–4.92) |
| *Perameles bougainville* + *P. eremiana* v *P. gunnii + P. nasuta* | 11.4 (8.54–14.4) | 11.86 (8.95–14.98) | 11.37 (8.61–14.36) |
| *P. bougainville* v *P. eremiana* | 8.49 (5.04–12.07) | 8.83 (5.22–12.59) | 8.49 (5.04–12.03) |
| *P. gunnii* v *P. nasuta* | 4.72 (2.91–7.04) | 4.84 (2.98–7.24) | 4.68 (2.73–6.85) |

**Supplementary Table S8. Results of alternative revised constraints for Chaeropodidae and Peramelemorphia using stratigraphical bracketing (SB) for Dasyuridae.** Minimum–maximum values are shown in Supplementary Table S5. Divergence date estimates (million years BP) are graphically represented in Supplementary Figs S28–S30.

| **Node** | **Divergence Estimates** | | |
| --- | --- | --- | --- |
|  | **Chaeropodidae** | **Peror. + Echym.**  **/ Peram. / Thylaco. / Chaeropodidae** | **Peramelemorpha (SB for Dasyuridae)** |
| Dasyuromorphia v Peramelemorphia | 60.17 (54.47–65.64) | 60.34 (54.74–65.79) | 60.33 (54.66–65.81) |
| *Chaeropus* v *Macrotis* + Peramelidae | 37.66 (29.56–45.85) | 38.06 (30.14–46.32) | 38.02 (29.85–45.81) |
| *Macrotis* v Peramelidae | 31.26 (25.14–37.95) | 31.55 (25.76–38.24) | 31.61 (25.56–38.04) |
| Peramelinae v Peroryctinae + Echymiperinae | 20.14 (16.55–24.12) | 20.49 (16.77–24.43) | 20.32 (16.74–24.54) |
| Peroryctinae v Echymiperinae | 17.31 (14.07–20.99) | 17.55 (14.08–21.08) | 17.43 (14.12–21.27) |
| *Peroryctes broadbenti* v *P. raffrayana* | 7.98 (5.13–11.31) | 8.08 (5.08–11.62) | 8.02 (5.07–11.56) |
| *Rhynchomeles + Echymipera* v *Microperoryctes* | 12.09 (9.62–14.99) | 12.21 (9.62–15.14) | 12.22 (9.64–15.1) |
| *Rhynchomeles* v *Echymipera* | 10.62 (7.9–13.59) | 10.7 (7.92–13.8) | 10.77 (7.94–13.7) |
| *E. clara* v *E. kalubu* + *E. rufescens* | 9.78 (7.39–12.53) | 9.85 (7.36–12.59) | 9.93 (7.49–12.58) |
| *E. kalubu* v *E. rufescens* | 6.33 (4.09–8.71) | 6.4 (4.18–8.85) | 6.45 (4.34–8.8) |
| *Microperoryctes* sp. (Tembagapura) v *M. ornata + M. longicauda* (Sol) *+ M. papuensis* | 8.05 (5.8–10.62) | 8.18 (5.84–10.74) | 8.15 (5.81–10.63) |
| *M. ornata + M. longicauda* (Sol) v *M. papuensis* | 4.93 (3.27–6.88) | 5.01 (3.35–6.95) | 4.98 (3.31–6.82) |
| *M. ornata* v *M. longicauda* (Sol) | 2.14 (1.19–3.38) | 2.17 (1.15–3.42) | 2.18 (1.21–3.42) |
| *Isoodon* v *Perameles* | 13.12 (10.28–16.27) | 13.73 (10.84–16.91) | 13.24 (10.22–16.4) |
| *I. auratus* v *I. macrourus + I. obesulus* | 5.15 (3.49–7.17) | 5.25 (3.46–7.3) | 5.24 (3.52–7.26) |
| *I. macrourus* v *I. obesulus* | 3.27 (1.95–4.85) | 3.31 (1.94–4.94) | 3.31 (1.98–4.93) |
| *Perameles bougainville* + *P. eremiana* v *P. gunnii + P. nasuta* | 11.34 (8.6–14.32) | 11.83 (9–14.8) | 11.45 (8.62–14.55) |
| *P. bougainville* v *P. eremiana* | 8.43 (4.98–11.91) | 8.81 (5.27–12.51) | 8.54 (5.07–12.15) |
| *P. gunnii* v *P. nasuta* | 4.71 (2.9–6.95) | 4.77 (2.82–7.12) | 4.68 (2.81–6.95) |

**Supplementary Table S9. Ancestral area results for crown Peramelemorphia and its constituent ingroup clades based on the parsimony consensus tree of morphology.** S-DIVA was not employed because of polytomies. Bayesian Binary MCMC (BBM) analyses are shown with posterior probability (PP) values returned at each node. Superscript: ^M^multi-area; ^S^single area. Graphic tree output is presented in Supplementary Fig. S31.

| **Node** | **Ancestral Area Optimization** | | | |
| --- | --- | --- | --- | --- |
|  | **BBM^M^** | **PP** | **BBM^S^** | **PP** |
| Dasyuromorphia v Peramelemorphia | AB  A  * | 0.8225  0.1323  0.0425 | A  B  C  D | 0.9592  0.8614  0.0057  0.0044 |
| *Macrotis lagotis* v *Macrotis leucura* | D  * | 0.9530  0.0470 | A  B  C  D | 0.0317  0.0112  0.0026  0.9571 |
| *Microperoryctes* sp. (Tembagapura) v *M. ornata* v *M. longicauda* (Sol) v *M. papuensis* | A  * | 0.9943  0.0057 | A  B  C  D | 0.9987  0.0046  0.0005  0.0006 |
| *Perameles bogainville* + *P. eremiana* + *Chaeropus* + *Isoodon auratus* v *I. obesulus* + *I. macrourus* | BCD  CD  BD  D  * | 0.4256  0.3867  0.0767  0.0697  0.0413 | A  B  C  D | 0.0026  0.5239  0.8472  0.9548 |
| *I. obesulus* v *I. macrourus* | BCD  BC  * | 0.9946  0.1523  0.0054 | A  B  C  D | 0.0006  0.9972  0.9988  0.9443 |

Area codes: A = rainforest; B = woodland (referring primarily to sclerophyll forests); C = shrubland (including ‘open’ and xeromorphic vegetation); D = arid/semi-arid vegetation and desert.

**Supplementary Table S10. Ancestral area results for crown Peramelemorphia and its constituent ingroup clades based on Bayesian morphology trees.** Both S-DIVA and BBM analyses are shown with PP values returned at each node. Graphic tree output is presented in Supplementary Fig. S32.

| **Node** | **Ancestral Area Optimization** | | | | | |
| --- | --- | --- | --- | --- | --- | --- |
|  | **S-DIVA** | **PP** | **BBM^M^** | **PP** | **BBM^S^** | **PP** |
| Dasyuromorphia v Peramelemorphia | AD  AB  BD  BCD  AC  ACD  * | 0.272  0.2191  0.1399  0.106  0.0981  0.0763  0.0886 | A  AB  * | 0.5104  0.3362  0.1534 | A  B  C  D | 0.8896  0.3971  0.0458  0.0620 |
| *Echymipera* v *Rhynchomeles + Peroryctes* + *Microperoryctes* | A  * | 0.9889  0.0111 | A  AB  * | 0.7633  0.2226  0.0141 | A  B  C  D | 0.9691  0.2258  0.0033  0.0036 |
| *E. clara* v *E. kalubu* + *E. rufescens* | A | 1 | A  AB  * | 0.785  0.2131  0.0019 | A  B  C  D | 0.9988  0.2135  0.0009  0.0008 |
| *E. kalubu* v *E. clara* | A | 1 | A  AB  * | 0.877  0.1212  0.0018 | A  B  C  D | 0.9982  0.1214  0.0008  0.0008 |
| *Peroryctes broadbenti* v *Rhynchomeles* + *P. raffrayana* + *Microperoryctes* | A  AB | 0.7727  0.2273 | A  AB  * | 0.696  0.2637  0.0403 | A  B  C  D | 0.8788  0.2747  0.0019  0.0017 |
| *Rhynchomeles* + *P. raffrayana* + *Microperoryctes* | AB | 1 | A  * | 0.9623  0.0377 | A  B  C  D | 0.9959  0.0364  0.0006  0.0005 |
| *P. raffrayana* v *Microperoryctes* | A | 1 | A  * | 0.9681  0.0319 | A  B  C  D | 0.999  0.0304  0.0008  0.0007 |
| *Microperoryctes* sp. (Tembagapura) v *M. longicauda* (Sol) | A | 1 | A  * | 0.9978  0.0022 | A  B  C  D | 0.9985  0.0013  0.0004  0.0004 |
| *P. raffrayana* v *M. ornata* + *M. papuensis* | A | 1 | A  AB  * | 0.8805  0.1177  0.0018 | A  B  C  D | 0.9989  0.1179  0.0009  0.0008 |
| *M. ornata* v *M. papuensis* | A | 1 | A  * | 0.9947  0.0053 | A  B  C  D | 0.9995  0.0045  0.0004  0.0004 |
| *Macrotis* + *Perameles gunnii* v *P. nasuta* v *P. bougainville + P. eremiana + Isoodon* | D  CD  * | 0.7630.2231  0.0133 | D  CD  C  BD  * | 0.3334  0.2436  0.1553  0.0502  0.2175 | A  B  C  D | 0.1036  0.1308  0.4222  0.6107 |

| **Node (Continued)** | **Ancestral Area Optimization** | | | | | |
| --- | --- | --- | --- | --- | --- | --- |
|  | **S-DIVA** | **PP** | **BBM^M^** | **PP** | **BBM^S^** | **PP** |
| *Macrotis* v *P. gunnii* v *P. nasuta* | CD  BCD  BD | 0.7561  0.1415  0.1024 | D  CD  C  * | 0.3921  0.2851  0.202  0.1208 | A  B  C  D | 0.0096  0.0857  0.4211  0.5853 |
| *P. gunnii* v *P. nasuta* | C  * | 0.9917  0.0083 | C  BC  * | 0.729  0.2135  0.0575 | A  B  C  D | 0.002  0.2265  0.9609  0.0455 |
| *Perameles bougainville* v *P. eremiana* + *Isoodon* | D  CD  * | 0.7665  0.2183  0.0152 | CD  D  BCD  * | 0.7656  0.01423  0.0683  0.0238 | A  B  C  D | 0.0045  0.0819  0.8433  0.9923 |
| *I. macrourus* v *I. obesulus* | D  CD  * | 0.7738  0.2185  0.0077 | BCD  * | 0.9959  0.0041 | A  B  C  D | 0.0014  0.9990  0.9991  0.9993 |
| *Perameles bougainville* + *P. eremiana* v *P. gunnii + P. nasuta* | CD  C | 0.5  0.5 | CD  BCD  C  BC  * | 0.4137  0.2162  0.2086  0.1091  0.0524 | A  B  C  D | 0.0082  0.3433  0.9437  0.6647 |
| *Perameles eremiana* v *Isoodon* | CD  C | 0.5  0.5 | CD  D  BCD  BD  * | 0.5559  0.2094  0.1668  0.0628  0.0051 | A  B  C  D | 0.0027  0.2308  0.7264  0.9968 |
| *I. auratus* v *I. macrourus + I. obesulus* | D | 1 | BCD  * | 0.9667  0.0333 | A  B  C  D | 0.0010  0.9733  0.9948  0.9994 |
| *I. macrourus* v *I. obesulus* | D | 1 | BCD  BC  * | 0.9953  0.1445  0.0047 | A  B  C  D | 0.0008  0.9986  0.9983  0.9292 |

**Supplementary Table S11. Ancestral area results for Peramelemorphia and its constituent ingroup clades based on parsimony/Bayesian trees of combined morphology + DNA data.** Both S-DIVA and BBM analyses are shown with PP values returned at each node. Graphic tree output is presented in Supplementary Fig. S33.

| **Node** | **Ancestral Area Optimization** | | | | | |
| --- | --- | --- | --- | --- | --- | --- |
|  | **S-DIVA** | **PP** | **BBM^M^** | **PP** | **BBM^S^** | **PP** |
| Dasyuromorphia v Peramelemorphia | ACD  BCD  ABCD  CD  D  AD  BD | 0.1429  0.1429  0.1429  0.1429  0.1429  0.1429  0.1429 | AB  ABD  B  A  * | 0.4541  0.1704  0.1195  0.0974  0.1586 | A  B  C  D | 0.7916  0.8234  0.0657  0.2729 |
| *Chaeropus* + *Macrotis* v Peramelidae | BCD  ABCD  ACD  D  CD | 0.2  0.2  0.2  0.2  0.2 | BD  ABD  B  D  AB  AD  A  * | 0.1793  0.1529  0.1394  0.1298  0.1189  0.1107  0.0861  0.0829 | A  B  C  D | 0.4602  0.58  0.0752  0.5625 |
| *Macrotis* v *Chaeropus* | D | 1 | D  * | 0.945  0.055 | A  B  C  D | 0.0194  0.0259  0.0102  0.9905 |
| *Macrotis lagotis* v *Macrotis leucura* | D | 1 | D  * | 0.9959  0.0041 | A  B  C  D | 0.0012  0.0011  0.0018  0.9981 |
| Peramelinae v Peroryctinae + Echymiperinae | AD  BD  ABD  AC  BC  ABC | 0.1667  0.1667  0.1667  0.1667  0.1667  0.1667 | AB  B  ABD  BD  A  AD  D  * | 0.1719  0.1567  0.1369  0.1272  0.0769  0.0624  0.0569  0.2084 | A  B  C  D | 0.5231  0.691  0.1948  0.4481 |
| Peroryctinae v Echymiperinae | AB  A | 0.5  0.5 | AB  A  B  * | 0.5439  0.2767  0.1215  0.0579 | A  B  C  D | 0.8174  0.6628  0.0158  0.0395 |
| *Peroryctes broadbenti* v *P. raffrayana* | AB  B | 0.5  0.5 | AB  B  * | 0.7542  0.2302  0.0156 | A  B  C  D | 0.7661  0.9846  0.0017  0.0021 |
| *Rhynchomeles + Echymipera* v *Microperoryctes* | A | 1 | A  * | 0.9578  0.0422 | A  B  C  D | 0.9931  0.0394  0.0012  0.0015 |
| *Rhynchomeles* v *Echymipera* | A | 1 | A  * | 0.9947  0.0053 | A  B  C  D | 0.9986  0.0039  0.0008  0.0007 |

| **Node (Continued)** | **Ancestral Area Optimization** | | | | | |
| --- | --- | --- | --- | --- | --- | --- |
|  | **S-DIVA** | **PP** | **BBM^M^** | **PP** | **BBM^S^** | **PP** |
| *Rhynchomeles* v *E. kalubu* + *E. rufescens* | A | 1 | A  AB  * | 0.9211  0.0774  0.0015 | A  B  C  D | 0.9988  0.0775  0.0007  0.0007 |
| *Rhynchomeles* v *E. kalubu* | A | 1 | A  * | 0.9903  0.0097 | A  B  C  D | 0.9992  0.0087  0.0005  0.0005 |
| *Microperoryctes* sp. (Tembagapura) v *M. ornata + M. longicauda* (Sol) *+ M. papuensis* | A | 1 | A  * | 0.9973  0.0027 | A  B  C  D | 0.9986  0.0013  0.0007  0.0007 |
| *M. ornata + M. longicauda* (Sol) v *M. papuensis* | A | 1 | A  * | 0.9987  0.0013 | A  B  C  D | 0.9992  0.0004  0.0004  0.0004 |
| *M. ornata* v *M. longicauda* (Sol) | A | 1 | A  * | 0.9990  0.0010 | A  B  C  D | 0.9994  0.0003  0.0003  0.0003 |
| *Isoodon* v *Perameles* | D  C | 0.5  0.5 | BCD  CD  BC  BD  * | 0.5116  0.1432  0.1297  0.0623  0.1532 | A  B  C  D | 0.0834  0.7813  0.8915  0.7978 |
| *I. auratus* v *I. macrourus + I. obesulus* | D  C | 0.5  0.5 | BCD  * | 0.9852  0.0148 | A  B  C  D | 0.0059  0.9963  0.9979  0.9969 |
| *I. macrourus* v *I. obesulus* | D  C | 0.5  0.5 | BCD  BC  * | 0.9959  0.1568  0.0041 | A  B  C  D | 0.0014  0.9990  0.9991  0.8943 |
| *Perameles bougainville* + *P. eremiana* v *P. gunnii + P. nasuta* | CD  C | 0.5  0.5 | CD  BCD  C  BC  * | 0.4137  0.2162  0.2086  0.1091  0.0524 | A  B  C  D | 0.0082  0.3433  0.9437  0.6647 |
| *P. bougainville* v *P. eremiana* | CD  D | 0.5  0.5 | CD  D  * | 0.8161  0.1413  0.0426 | A  B  C  D | 0.0016  0.0316  0.8524  0.9987 |
| *P. gunnii* v *P. nasuta* | C | 1 | BC  C  * | 0.4768  0.4698  0.0534 | A  B  C  D | 0.0025  0.5037  0.9968  0.0494 |

**Supplementary Table S12. Ancestral area results for Peramelemorphia and its constituent ingroup clades based on Bayesian DNA trees with expanded outgroup sampling.** Both S-DIVA and BBM analyses are shown with PP values returned at each node. Graphic tree output is presented in Supplementary Fig. S34.

| **Node** | **Ancestral Area Optimization** | | | | | |
| --- | --- | --- | --- | --- | --- | --- |
|  | **S-DIVA** | **PP** | **BBM^M^** | **PP** | **BBM^S^** | **PP** |
| Dasyuromorphia v Peramelemorphia | BD | 1 | D  BD  AD  B  A  ABD  * | 0.4238  0.1624  0.1486  0.0768  0.0703  0.0558  0.0533 | A  B  C  D | 0.2556  0.2729  0.0223  0.6788 |
| *Chaeropus* v *Macrotis* + Peramelidae | D | 1 | D  * | 0.9112  0.0888 | A  B  C  D | 0.0314  0.0453  0.0119  0.9696 |
| *Macrotis* v Peramelidae | ACD  CD  ABCD  BCD  AD  D  ABD  BD | 0.125  0.125  0.125  0.125  0.125  0.125  0.125  0.125 | D  * | 0.9073  0.0927 | A  B  C  D | 0.0203  0.0442  0.0279  0.9638 |
| Peramelinae v Peroryctinae + Echymiperinae | ABD  BD  ACD  ABCD  BCD  AC  B  BC  AD  ABC | 0.1  0.1  0.1  0.1  0.1  0.1  0.1  0.1  0.1  0.1 | D  BD  CD  AD  * | 0.5517  0.1518  0.097  0.053  0.1465 | A  B  C  D | 0.0876  0.2158  0.1495  0.7918 |
| Peroryctinae v Echymiperinae | AB  A | 0.5  0.5 | A  AB  B  * | 0.4891  0.2278  0.1551  0.128 | A  B  C  D | 0.5949  0.3178  0.0103  0.0866 |
| *Peroryctes broadbenti* v *P. raffrayana* | AB  A | 0.5  0.5 | AB  B  * | 0.6139  0.3597  0.0264 | A  B  C  D | 0.6306  0.9681  0.0024  0.0037 |
| *Rhynchomeles + Echymipera* v *Microperoryctes* | A | 1 | A  * | 0.9848  0.0152 | A  B  C  D | 0.9894  0.0114  0.0015  0.0022 |
| *Rhynchomeles* v *Echymipera* | A | 1 | A  * | 0.9964  0.0036 | A  B  C  D | 0.9987  0.0021  0.0008  0.0007 |

| **Node (Continued)** | **Ancestral Area Optimization** | | | | | |
| --- | --- | --- | --- | --- | --- | --- |
|  | **S-DIVA** | **PP** | **BBM^M^** | **PP** | **BBM^S^** | **PP** |
| *E. clara* v *E. kalubu* + *E. rufescens* | A | 1 | A  * | 0.992  0.0080 | A  B  C  D | 0.9993  0.007  0.0006  0.0005 |
| *E. kalubu* v *E. rufescens* | A | 1 | A  AB  * | 0.9249  0.0735  0.0016 | A  B  C  D | 0.9989  0.0736  0.0008  0.0007 |
| *Microperoryctes* sp. (Tembagapura) v *M. ornata + M. longicauda* (Sol) *+ M. papuensis* | A | 1 | A  * | 0.9976  0.0024 | A  B  C  D | 0.9987  0.0009  0.0008  0.0007 |
| *M. ornata + M. longicauda* (Sol) v *M. papuensis* | A | 1 | A  * | 0.9986  0.0014 | A  B  C  D | 0.9994  0.0005  0.0005  0.0004 |
| *M. ornata* v *M. longicauda* (Sol) | A | 1 | A  * | 0.9988  0.0012 | A  B  C  D | 0.9995  0.0004  0.0004  0.0003 |
| *Isoodon* v *Perameles* | BD  CD  BCD  D  B  C  B  BC | 0.1429  0.1429  0.1429  0.1429  0.1429  0.1429  0.1429  0.1429 | CD  BCD  D  BD  * | 0.396  0.3853  0.0562  0.0547  0.1078 | A  B  C  D | 0.0113  0.4931  0.8757  0.8965 |
| *I. auratus* v *I. macrourus + I. obesulus* | BD  CD  BCD  B  C  D | 0.1667  0.1667  0.1667  0.1667  0.1667  0.1667 | BCD  * | 0.9480  0.052 | A  B  C  D | 0.0023  0.9897  0.9977  0.9624 |
| *I. macrourus* v *I. obesulus* | CD  BCD  BD  B  C | 0.2  0.2  0.2  0.2  0.2 | BCD  BC  * | 0.8459  0.1479  0.0062 | A  B  C  D | 0.002  0.9976  0.9984  0.8512 |
| *Perameles bougainville* + *P. eremiana* v *P. gunnii + P. nasuta* | CD  BCD  C  BD | 0.25  0.25  0.25  0.25 | CD  BCD  C  BC  * | 0.5384  0.1813  0.1671  0.0563  0.0569 | A  B  C  D | 0.0035  0.2519  0.9357  0.7631 |
| *P. bougainville* v *P. eremiana* | CD  D | 0.5  0.5 | CD  D  * | 0.8198  0.1386  0.0416 | A  B  C  D | 0.0014  0.0336  0.8554  0.9921 |

| **Node (Continued)** | **Ancestral Area Optimization** | | | | | |
| --- | --- | --- | --- | --- | --- | --- |
|  | **S-DIVA** | **PP** | **BBM^M^** | **PP** | **BBM^S^** | **PP** |
| *P. gunnii* v *P. nasuta* | BC  C | 0.5  0.5 | C  BC  * | 0.5261  0.403  0.0709 | A  B  C  D | 0.0019  0.4338  0.996  0.0673 |

**Supplementary Figures**


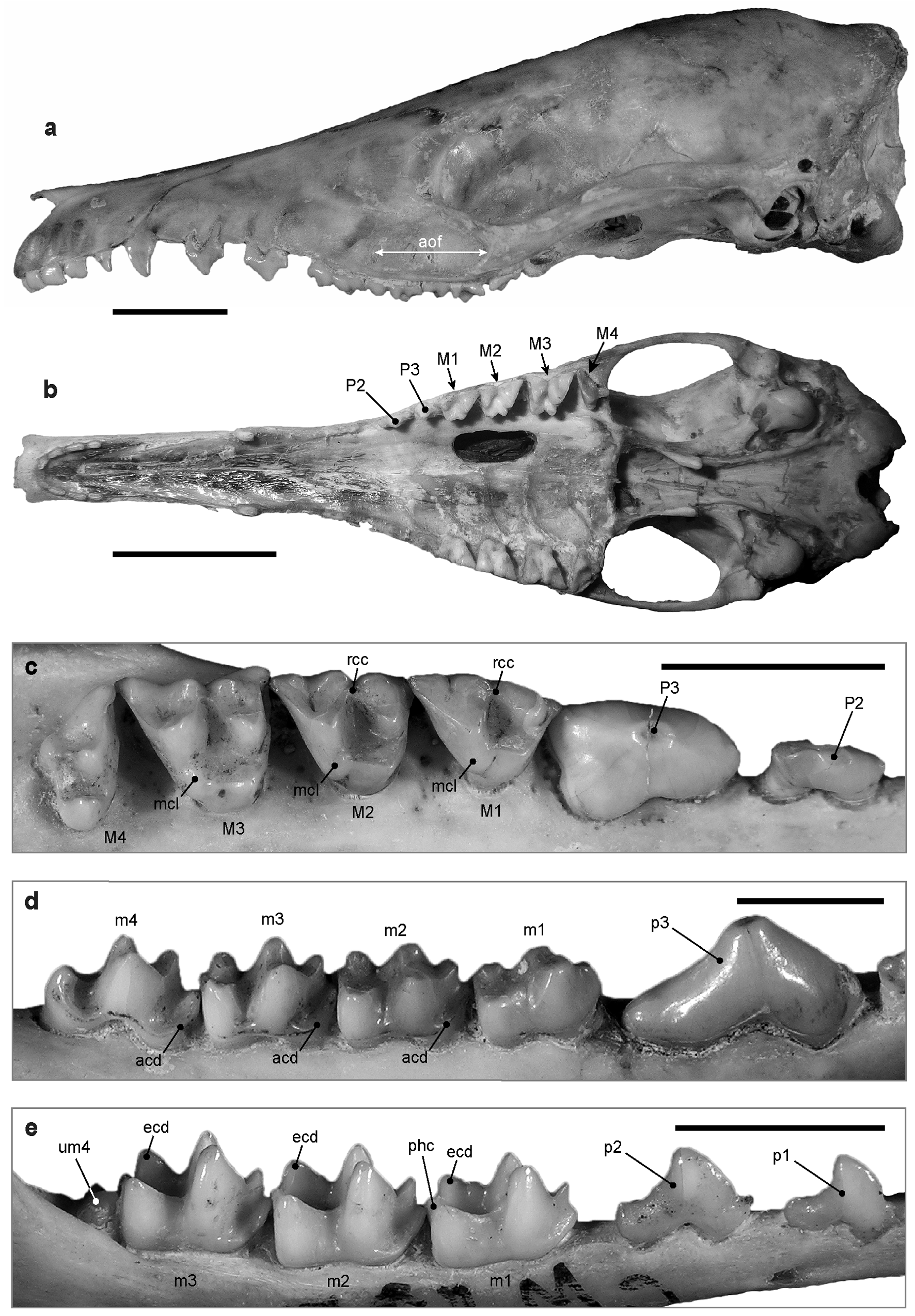


**Supplementary Figure S1. Comparative examples of the extant bandicoot species *Perpryctes broadbenti* (a, c, d), *Echymipera echinista* (b) and *P. raffrayana* (e).** (**a**) American Museum of Natural History (AMNH) 157132 subadult male *P. broadbenti* skull in lateral view. (**b**) University of Papua New Guinea (UP) 3075 adult female *E. echinista* holotype skull in occlusal view. (**c**) AMNH 157132 upper premolar (P) and molar (M) series of *P. broadbenti* in occlusal view. (**d**) AMNH 157134 lower premolar (p) and molar (m) series of an adult male *P. broadbenti* in lateral view. (**e**) Australian National Wildlife Collection (CSIRO) M12250 lower premolar/molar series of a juvenile male *P. raffrayana* in lateral view. Scale bars: 20 mm in **a**, **b**; 5 mm in **c**–**e**. Anatomical abbreviations: acd, anterior cingulid; aof, antorbital fossa; ecd, entoconid; mcl, metaconule; phc, posthypocristid; rcc, residual centrocrista; um4, unerupted m4. Images prepared from original photographs with (**c**–**e**) depicted in Aplin *et al*.^34^.


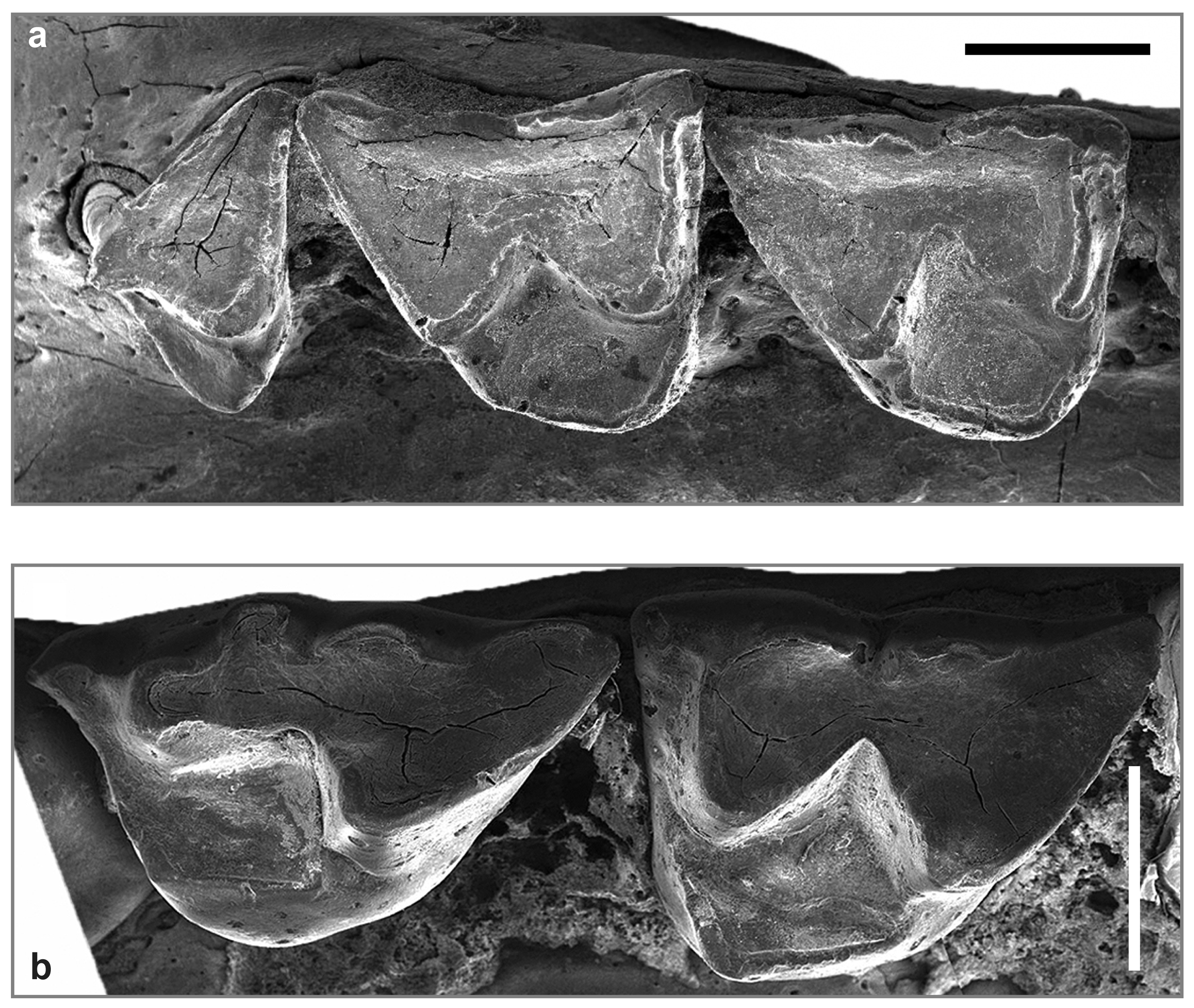


**Supplementary Figure S2. *Lemdubuoryctes aruensis* maxillary teeth imaged using SEM.** (**a**) WAM 14.9.9 showing M2–4 in occlusal view. (**b**) WAM 14.9.6 showing M1–2 in occlusal view. Scale bars: 2 mm.


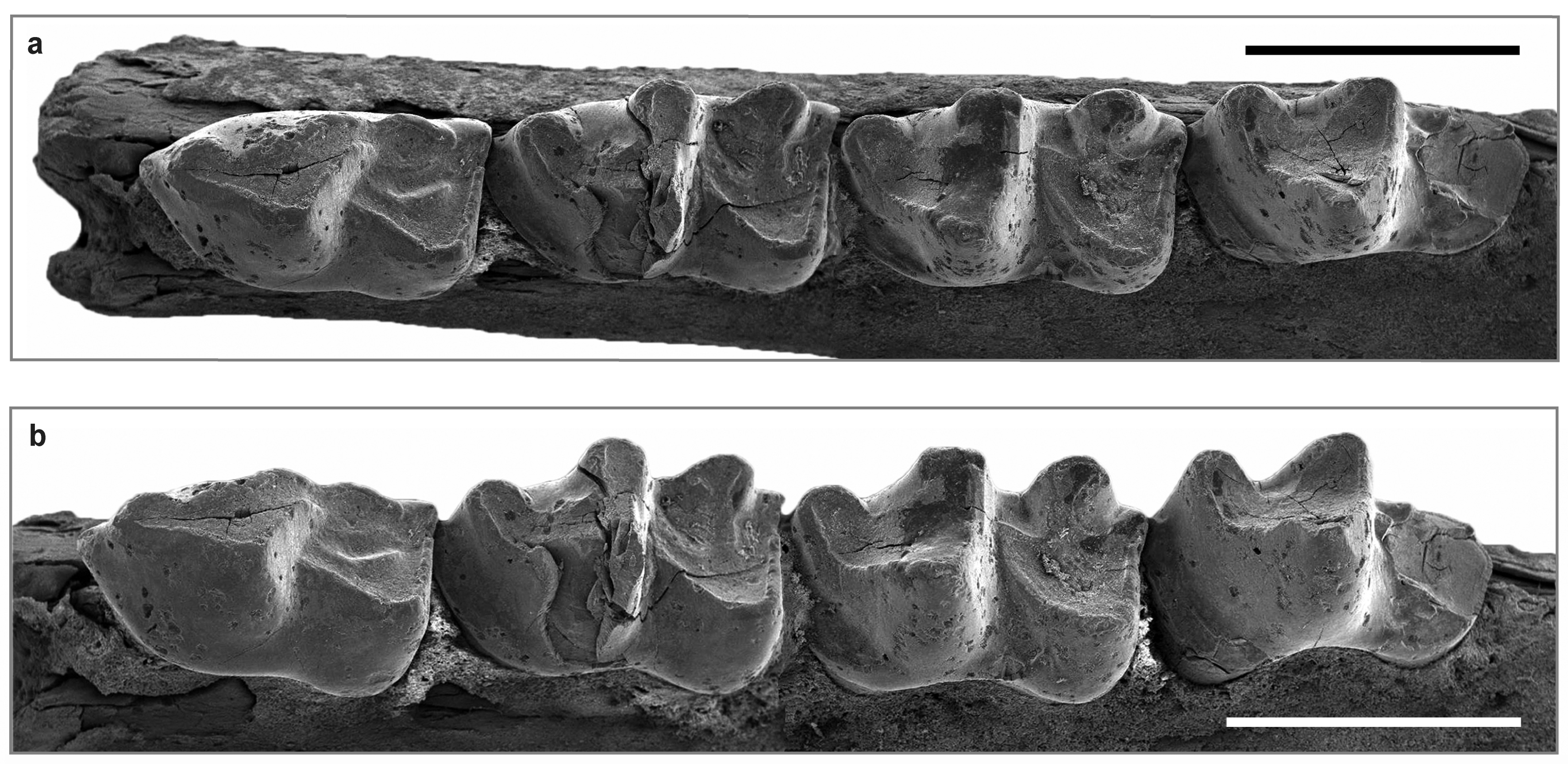


**Supplementary Figure S3. *Lemdubuoryctes aruensis* dentary teeth imaged using SEM.** (**a**) WAM 14.9.7 showing m1–4 in occlusal view. (**b**) WAM 14.9.7 m1–4 in oblique occlusal view. Scale bars: 4 mm.


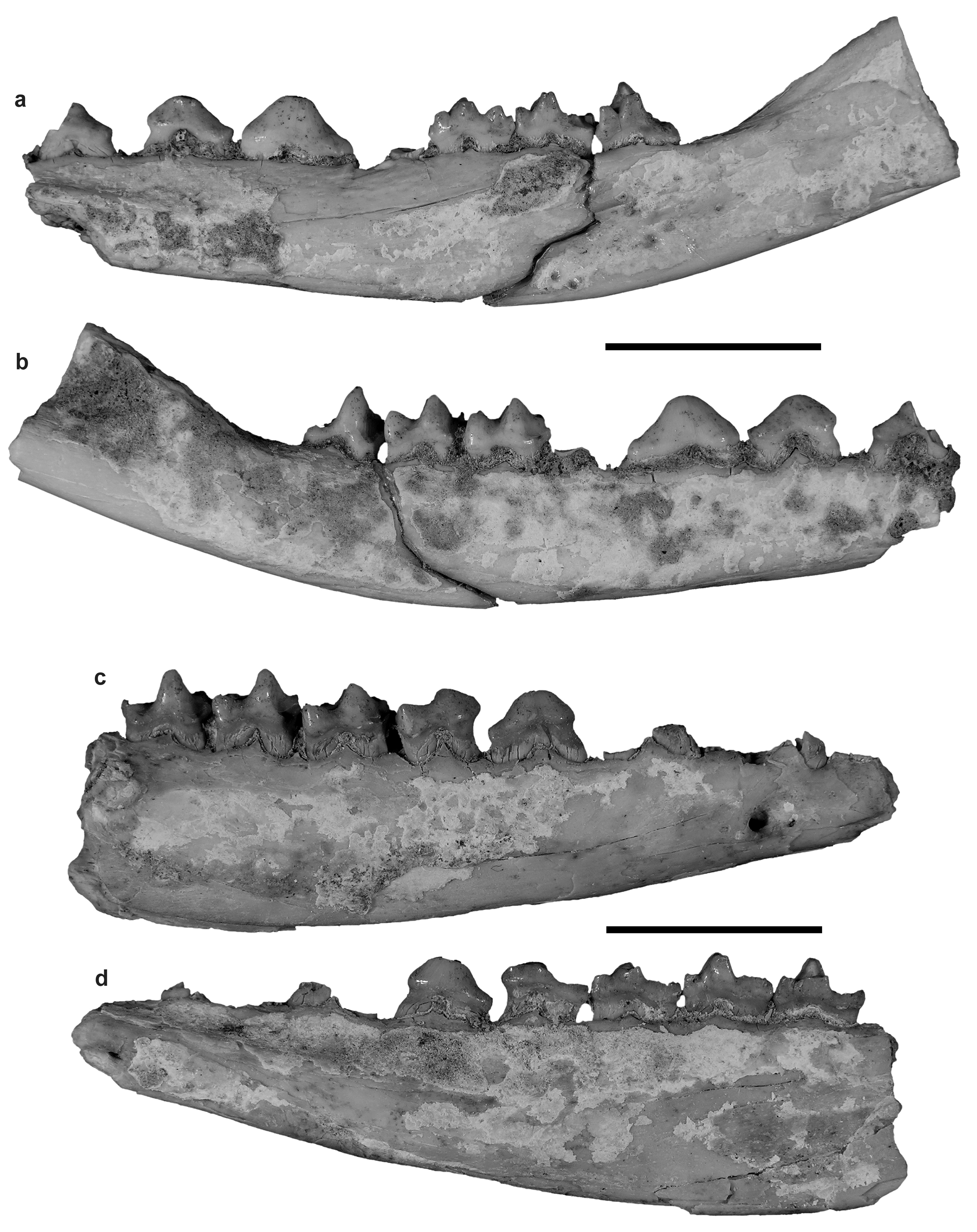


**Supplementary Figure S4. *Lemdubuoryctes aruensis* referred dentaries.** (**a**) WAM 14.9.1 in lingual and buccal views. (**b**) WAM 14.9.3 in buccal and lingual views. Scale bars: 10 mm.


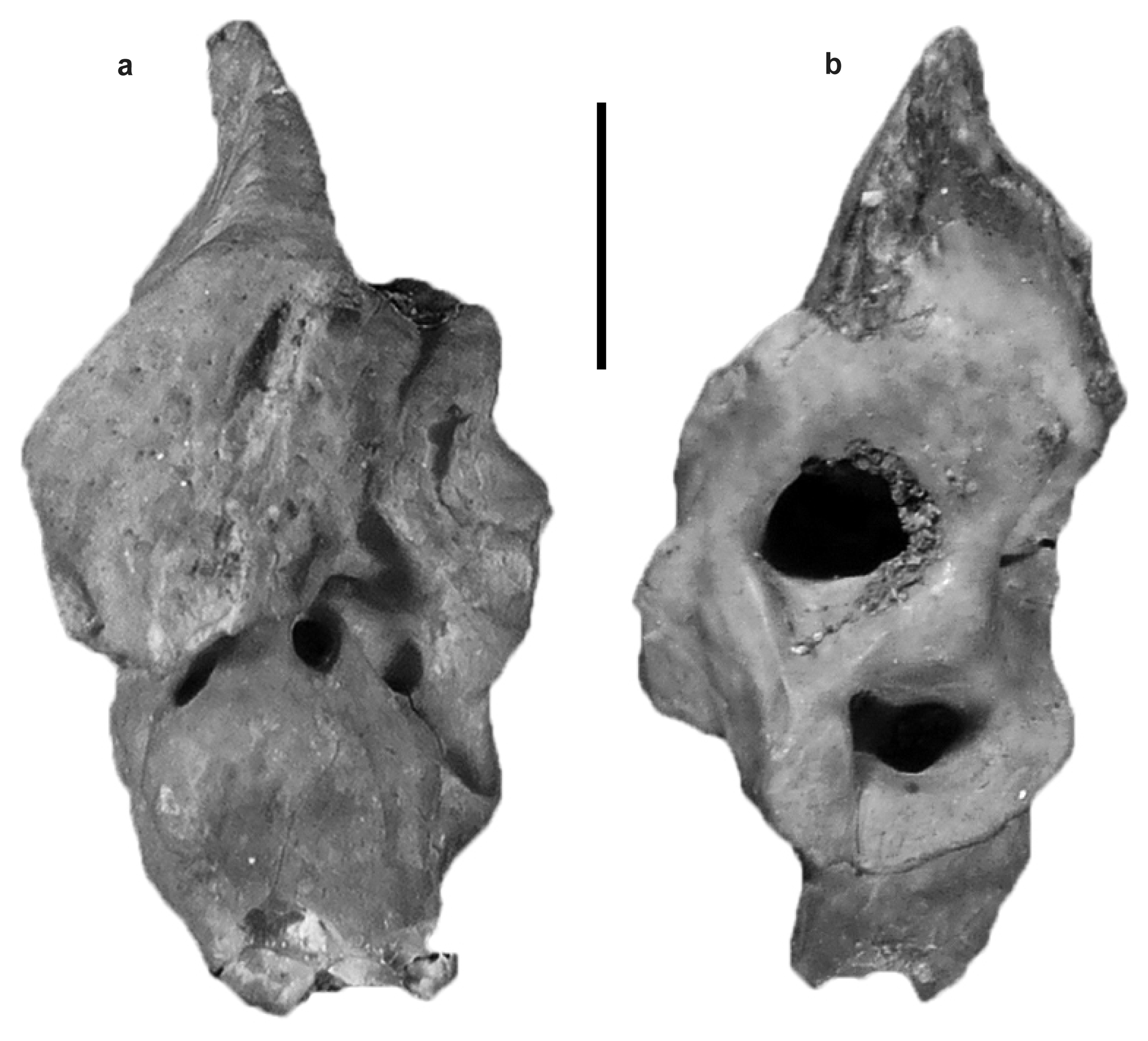


**Supplementary Figure S5. *Lemdubuoryctes aruensis* petrosal elements.** WAM 14.9.15 in (**a**) internal, and (**b**) external views. Scale bar: 2 mm.


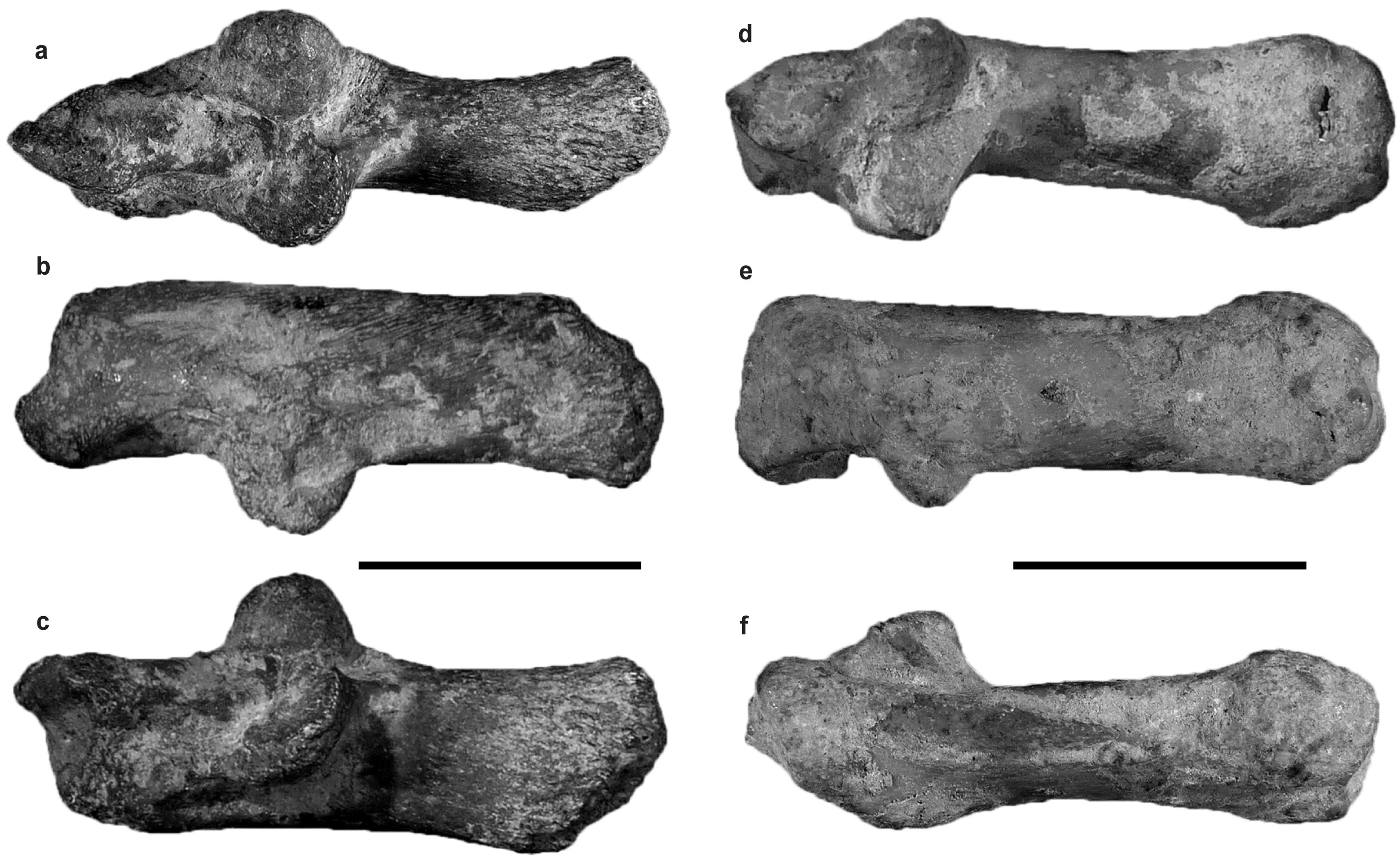


**Supplementary Figure S6. Isolated peramelemorphian calcanea from the Liang Lemdubu locality.** (**a–c**) WAM 14.9.16 *Lemdubuoryctes aruensis* (**d–f**) WAM 14.9.19 *Echymipera kalubu*. Both are shown in dorsal, lateral, and oblique views. Scale bars: 10 mm.


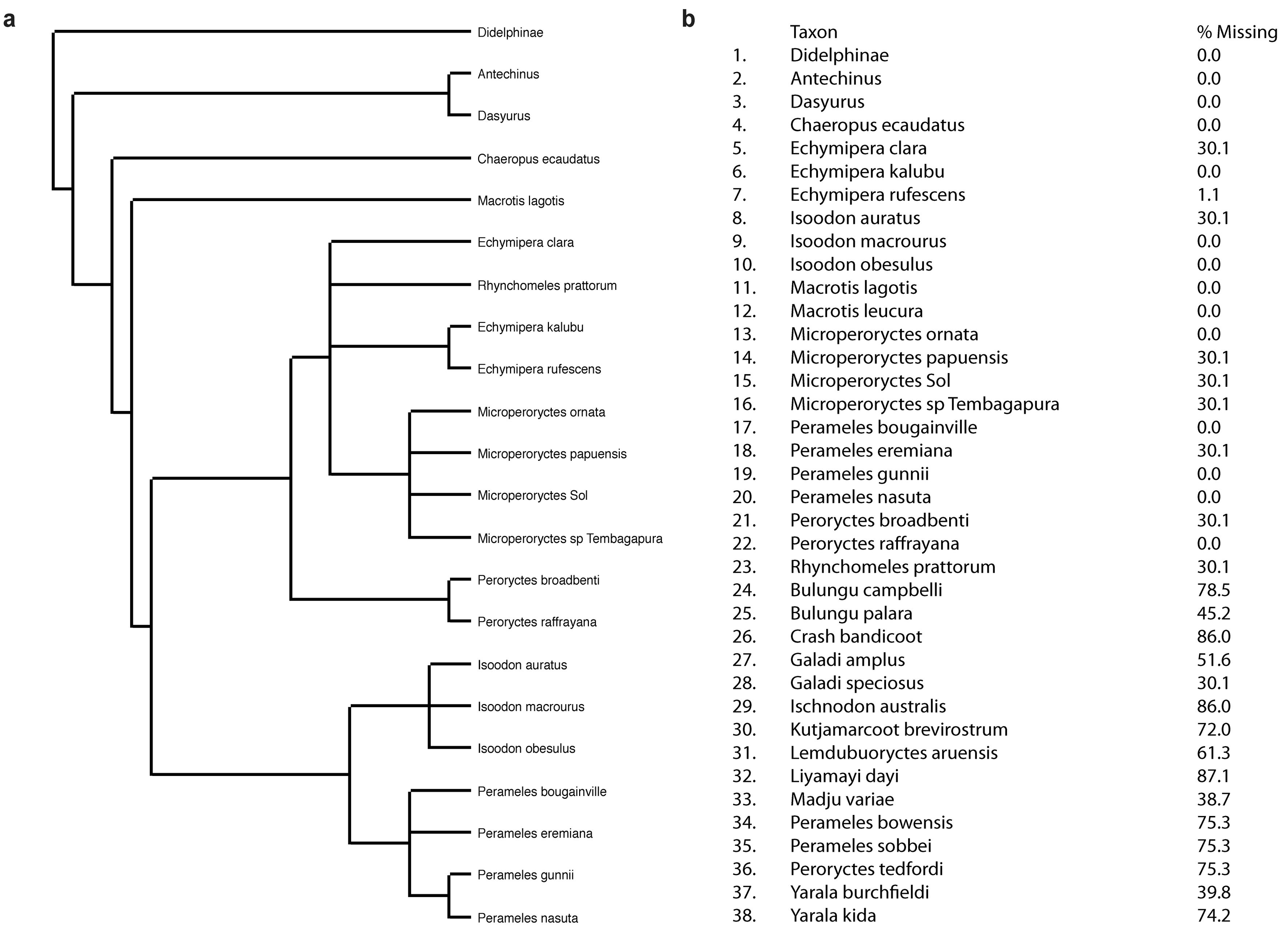


**Supplementary Figure S7. Constraint parameters output from MacClade ver.4.08a^97^.** (**a**) Molecular backbone topology based on Westerman *et al*.^1^ and our DNA dataset. (**b**) Taxon list indicating % missing data.


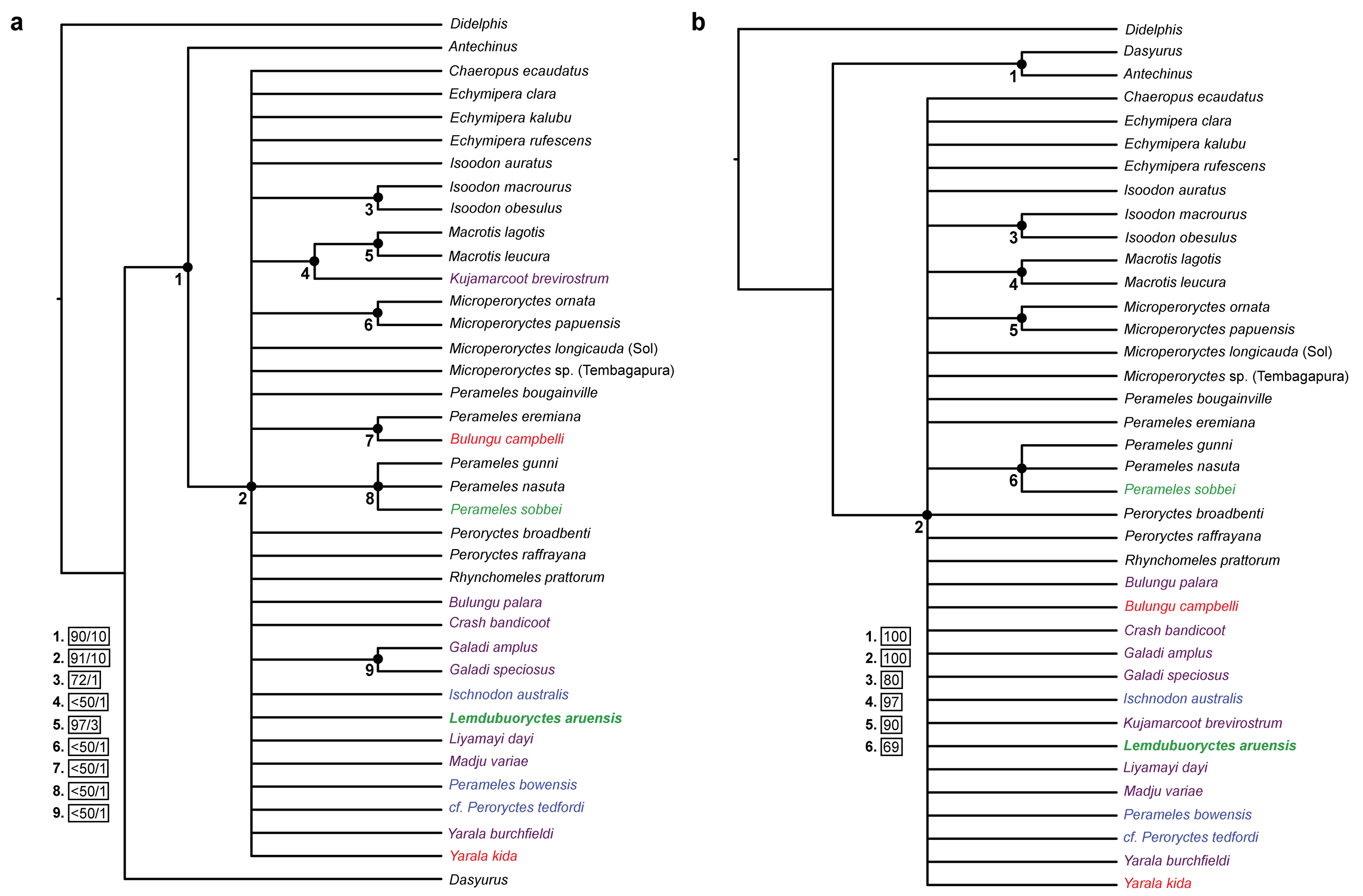


**Supplementary Figure S8. Parsimony strict consensus trees of the morphological data.** (**a**) Topology without constraints (Most parsimonious trees [MPTs] = >10000 [maxtrees], Length [L] = 268, Consistency index [CI] = 0.4407). (**b**) Molecular backbone enforced (MPTs = >10000, L = 296, CI = 0.4020). Extant or recently extinct taxa depicted in black. Fossils: Oligocene (red); Miocene (purple); Pliocene (blue); Pleistocene–Holocene (green). Boxed numbers represent (**a**) bootstrap/Bremer or (**b**) boostrap support values at resolved nodes.


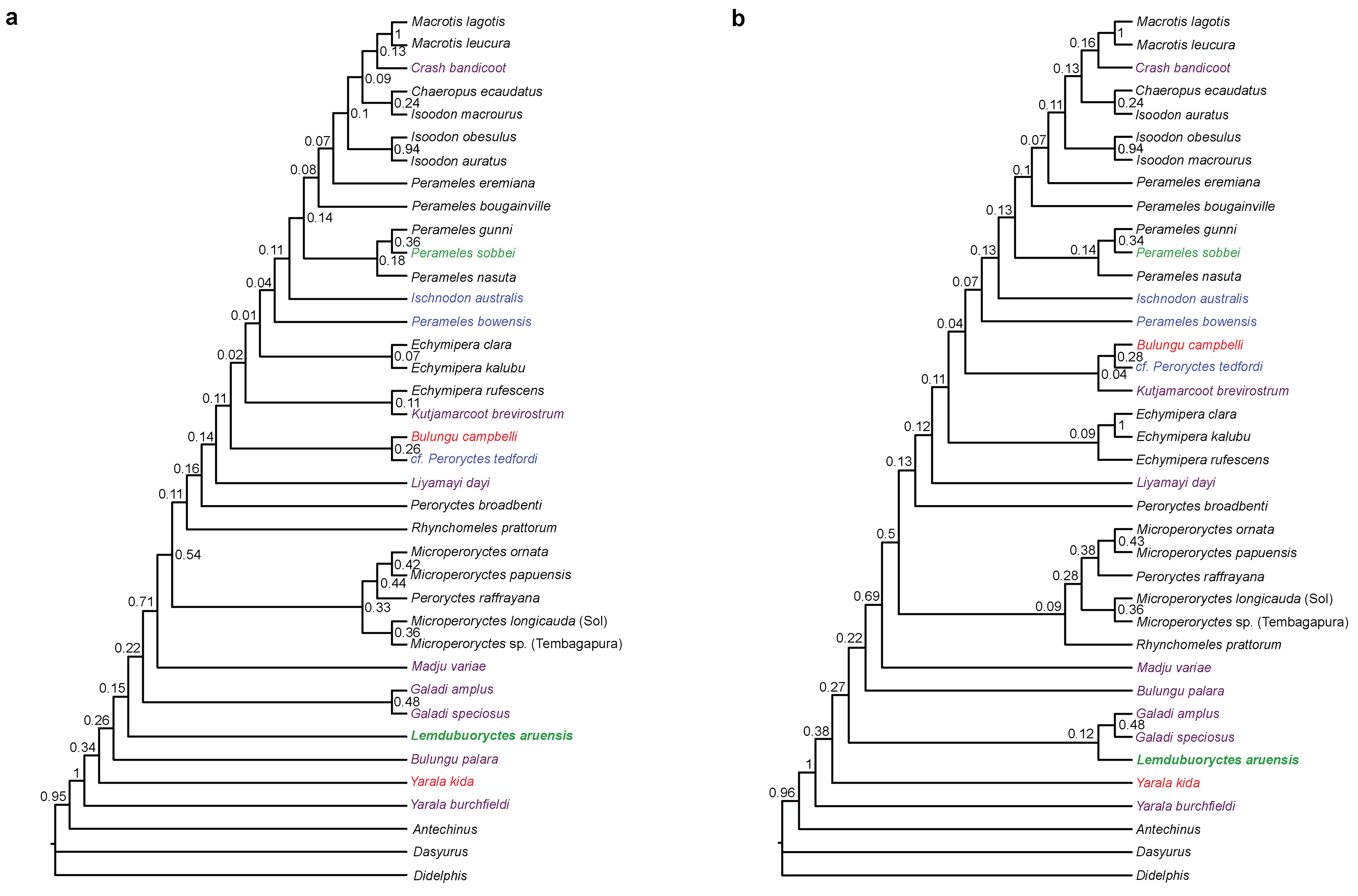


**Supplementary Figure S9. Bayesian consensus trees of the morphological data.** (**a**) Topology incorporating all taxa without constraints. (**b**) Molecular backbone enforced. Posterior probability support values are indicated at respective nodes.


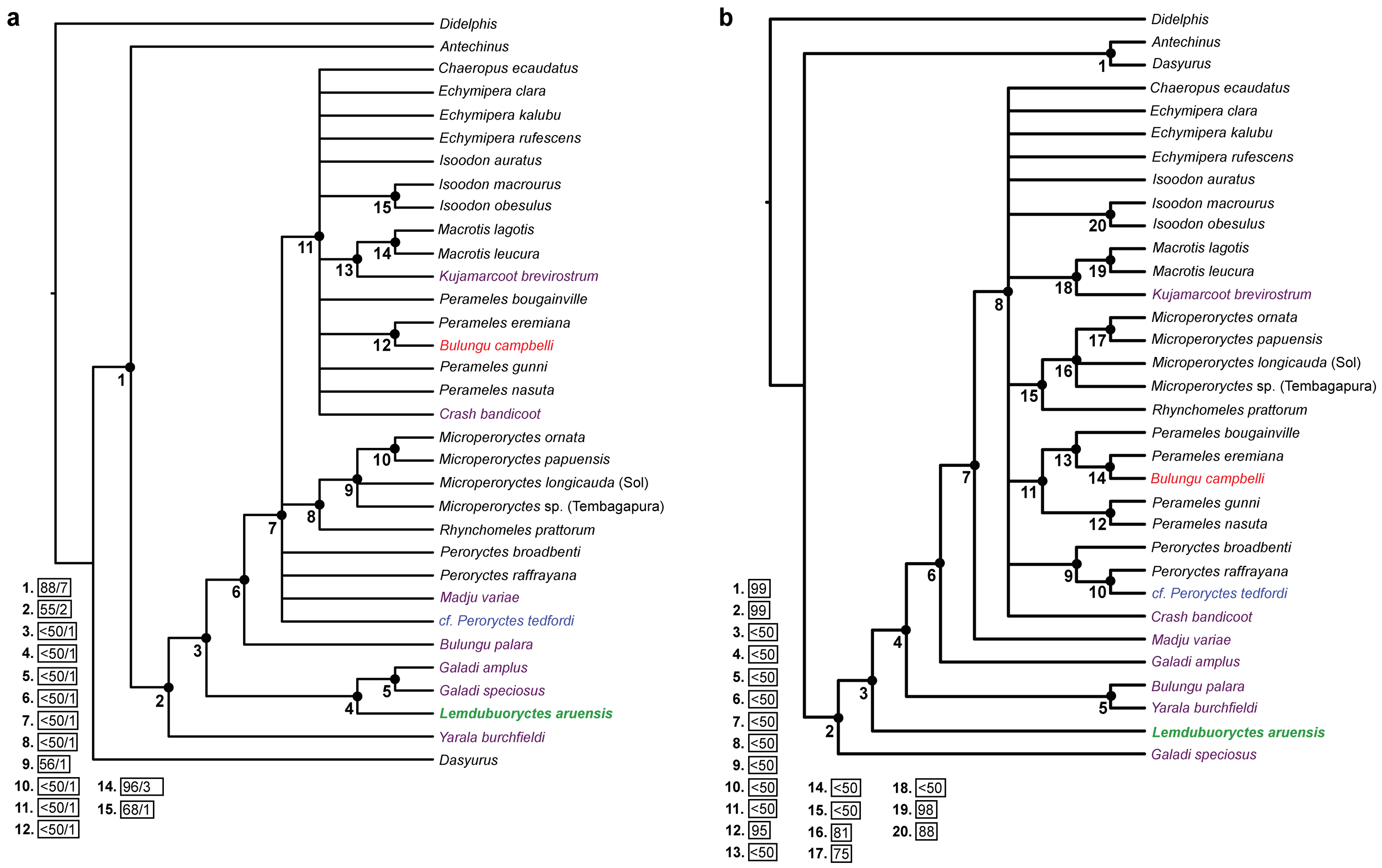


**Supplementary Figure S10. Parsimony strict consensus trees of the morphological data with redundant fossil taxa excluded.** (**a**) Topology without constraints (MPTs = 28, L = 260, CI = 0.4346). (**b**) Molecular backbone enforced (MPT = 4, L = 288, CI = 0.3937).


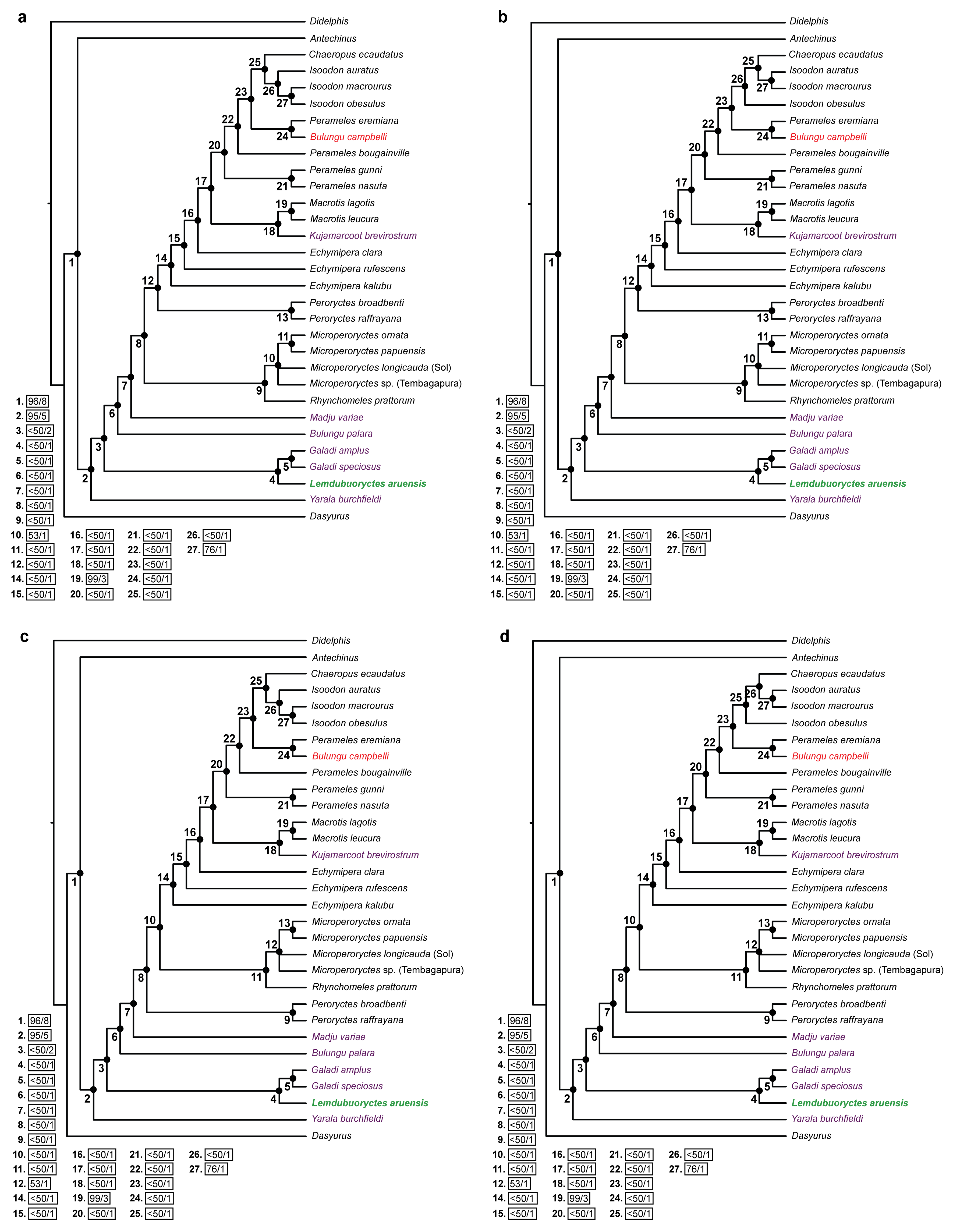


**Supplementary Figure S11. Parsimony best trees (a–d) derived from the morphological data with wildcard taxa pruned using assessment of Adams consensus results and % missing data (see Supplementary Fig. S7).** Topologies following deletion of the wildcard taxa *Crash bandicoot* and cf. *Peroryctes tedfordi* (MPTs = 4, L = 256, CI = 0.4375).


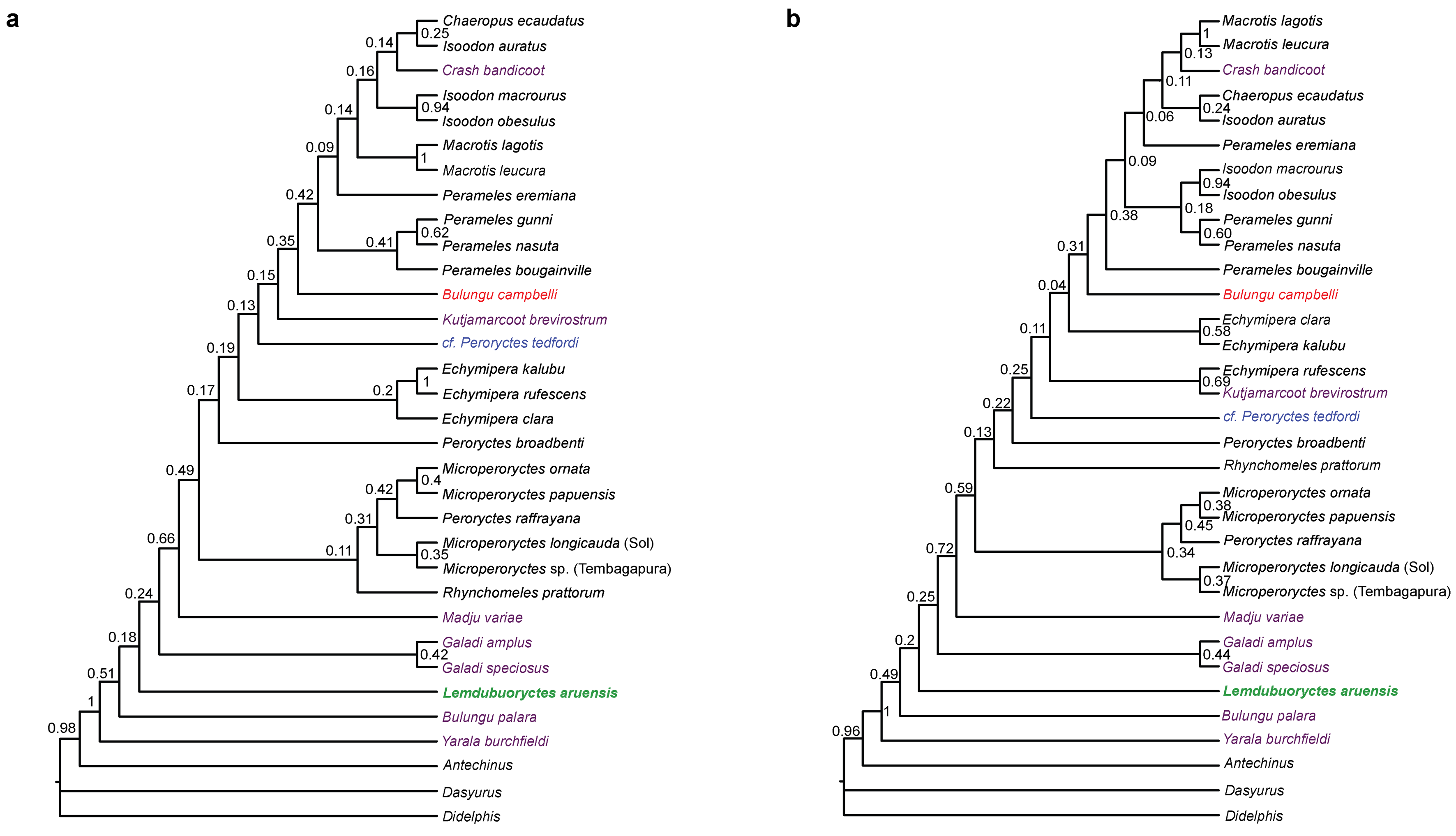


**Supplementary Figure S12. Bayesian consensus trees of the morphological data with redundant fossil taxa excluded.** (**a**) Topology without constraints. (**b**) Molecular backbone enforced. Posterior probability support values are indicated at respective nodes.

**
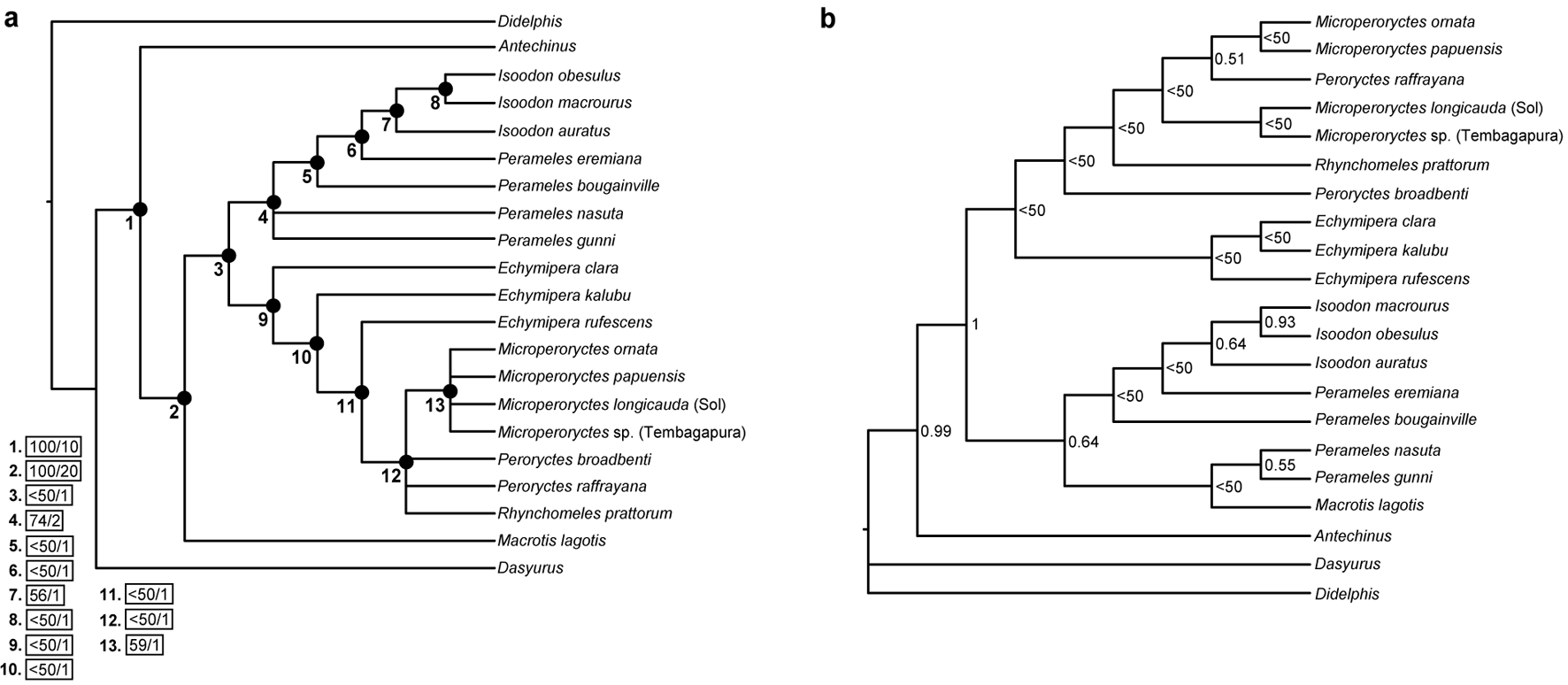
**

**Supplementary Figure S13. Parsimony strict (a)** **and Bayesian consensus trees (b)** **of the morphological data with all fossil taxa, *Chaeropus ecaudatus*, and *Macrotis leucura* excluded.** (**a**) Parsimony topology based on MPTs = 24, L = 216, CI = 0.4722. Node values = (**a**) bootstrap, and (**b**) posterior probability support at all respective nodes.


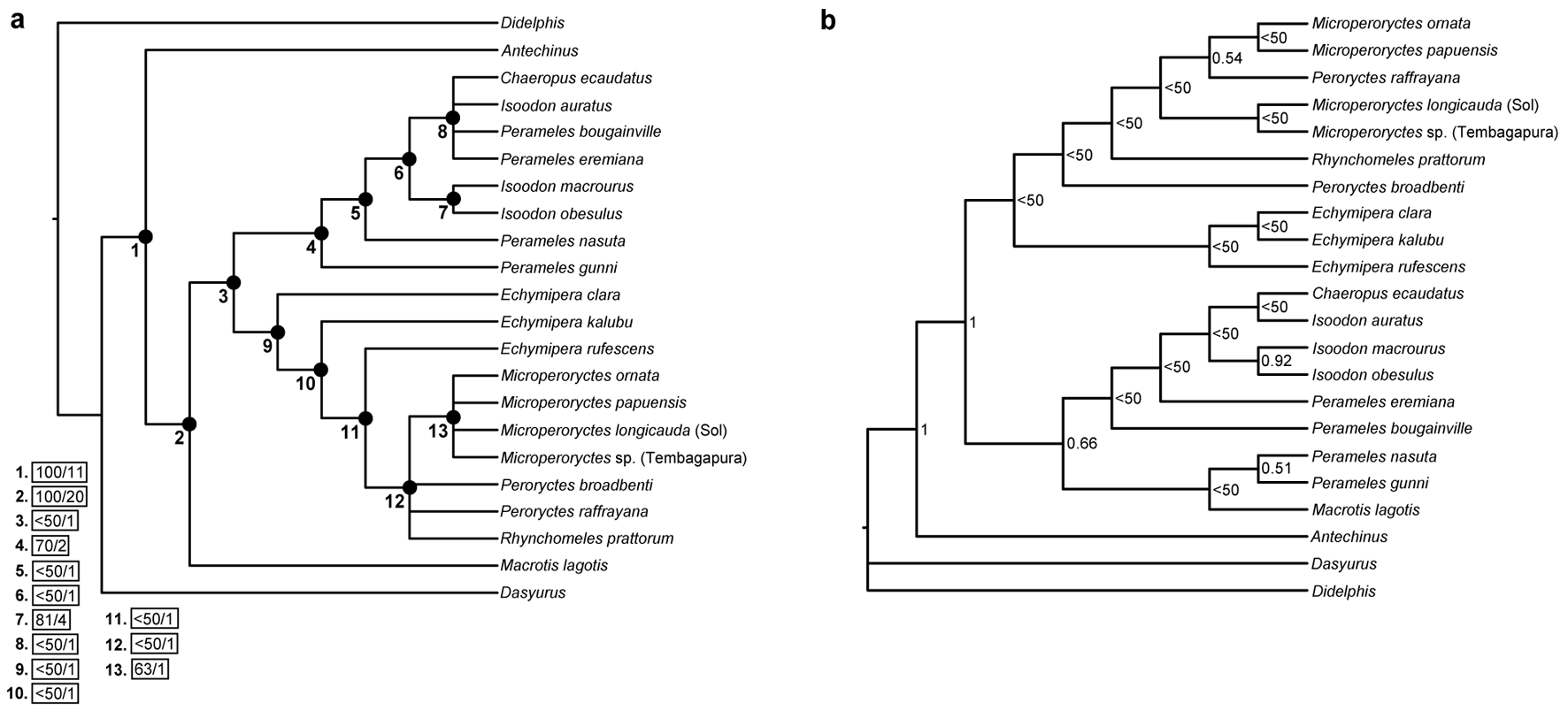


**Supplementary Figure S14. Parsimony strict (a)** **and Bayesian consensus trees (b)** **of the morphological data with all fossil taxa and *Macrotis leucura* excluded.** (**a**) Parsimony topology based on MPTs = 24, L = 216, CI = 0.4722. Node values = (**a**) bootstrap, and (**b**) posterior probability support at all respective nodes.


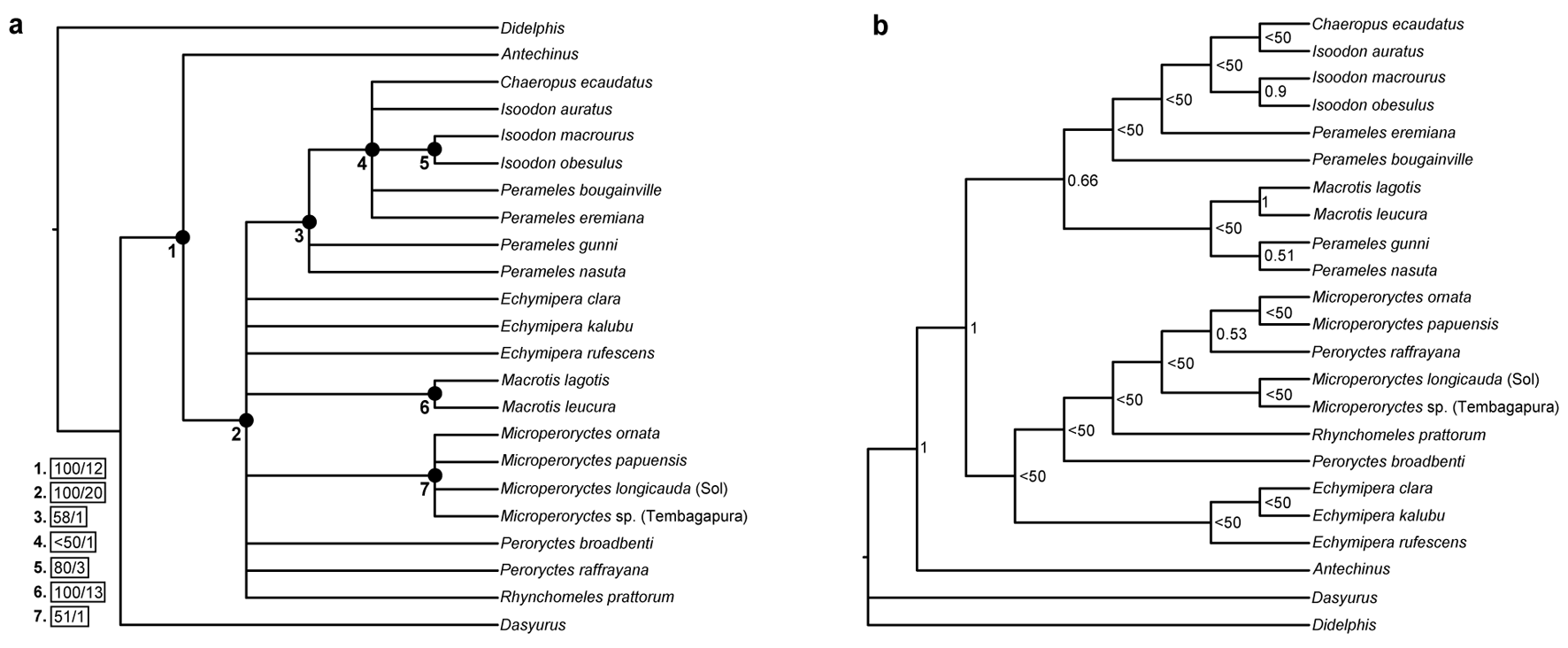


**Supplementary Figure S15. Parsimony strict (a)** **and Bayesian consensus trees (b)** **of the morphological data with all fossil taxa excluded, but retaining *Chaeropus ecaudatus* and *Macrotis leucura*.** (**a**) Parsimony topology based on MPTs = 23, L = 225, CI = 0.4756. Node values = (**a**) bootstrap, and (**b**) posterior probability support at all respective nodes.

**
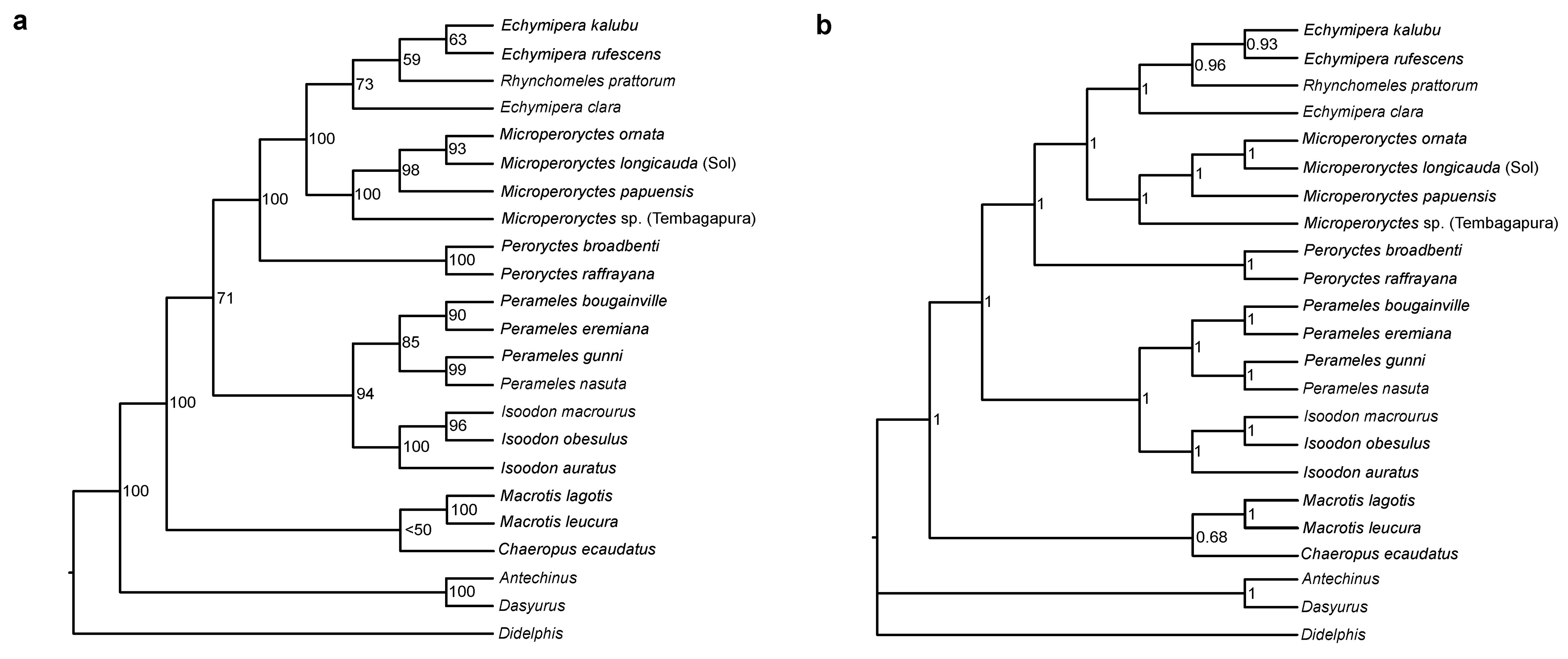
**

**Supplementary Figure S16. Parsimony strict consensus (a)** **and Bayesian consensus trees (b)** **of combined morphological + DNA data.** Excluding all fossils yielded MPT = 1, L = 5383, CI = 0.5681. Sequential exclusion of the recently extinct taxa *Macrotis leucura* (MPT = 1, L = 5379, CI = 0.5683), which was represented by morphology only, and *Chaeropus ecaudatus* (MPT = 1, L = 5399, CI = 0.5772), produced no chnge in topology. Node values = (**a**) bootstrap, and (**b**) posterior probability support at all respective nodes.

**
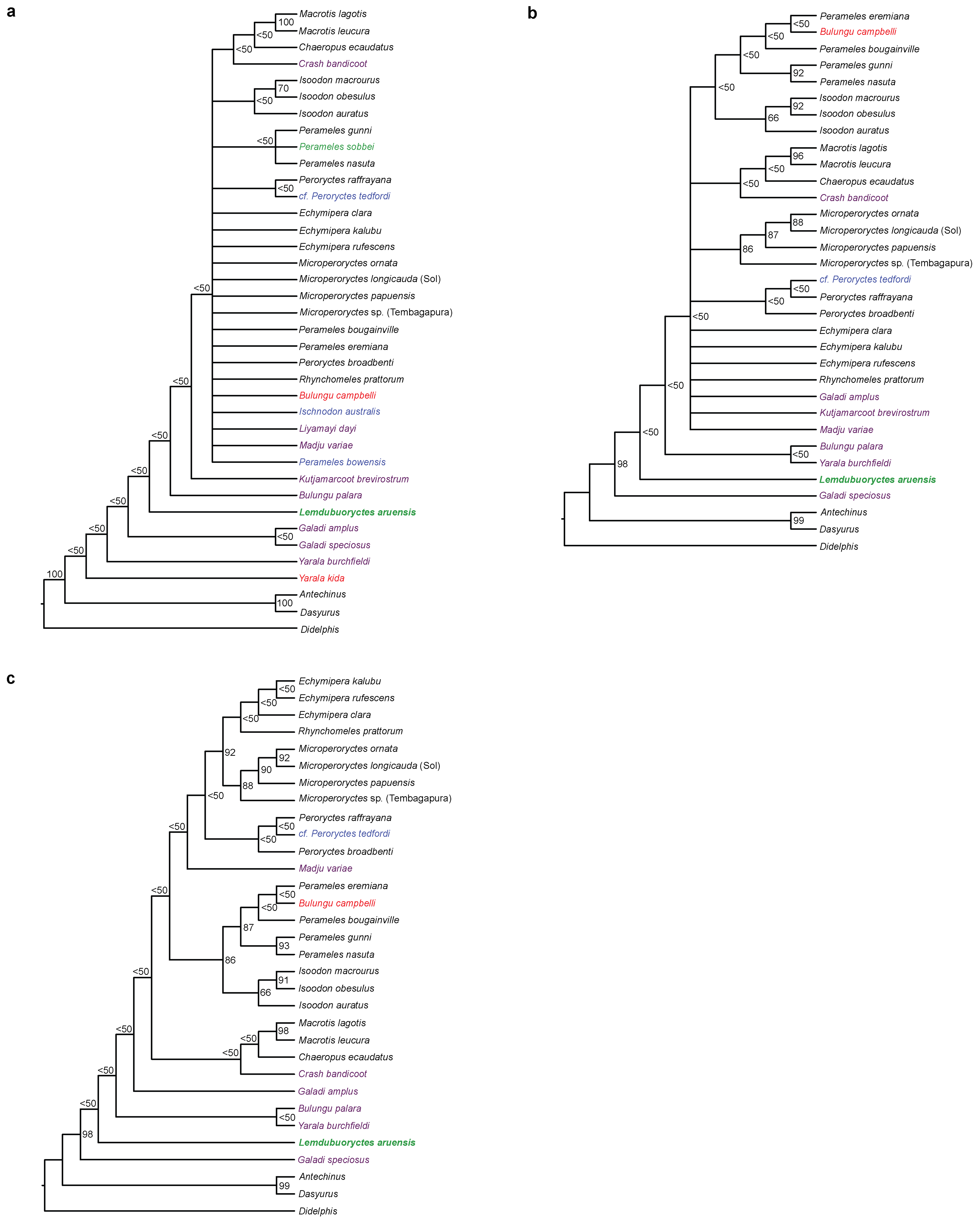
**

**Supplementary Figure S17. Parsimony strict consensus trees of the combined DNA + morphological data.** (**a**) Data set incorporating all taxa (MPTs = 149, L = 6880, CI = 0.6592). (**b**) Redundant fossil taxa excluded (MPTs = 3, L = 6875, CI = 0.6598). (**c**) Topology following deletion of *Kutjamarcoot brevisrostrum*, identified as a wildcard using assessment of Adams consensus results and % missing data (MPTs = 1, L = 6874, CI = 0.6599). Bootstrap support values are indicated at all respective nodes.

**
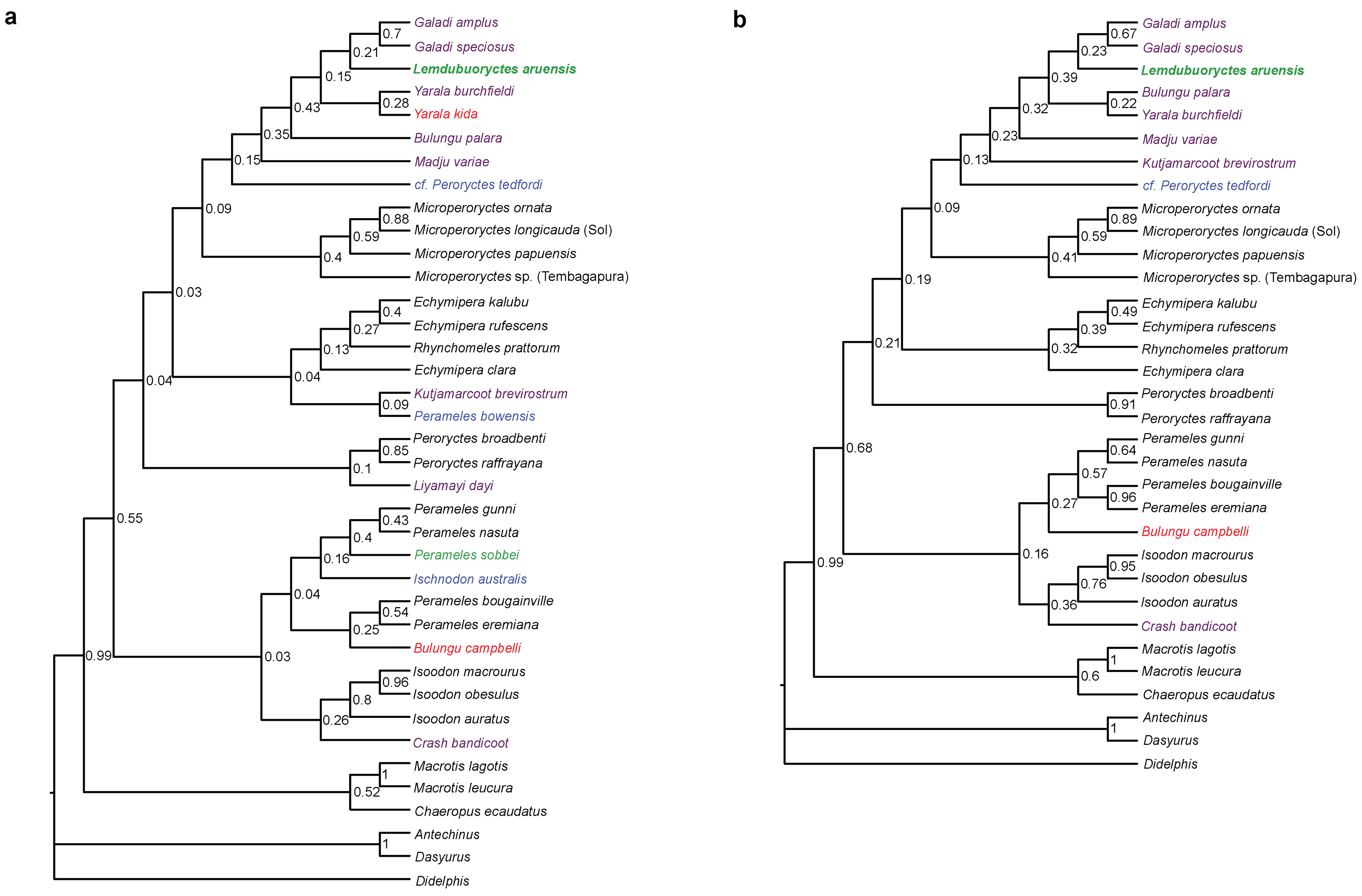
**

**Supplementary Figure S18. Bayesian consensus trees of combined DNA + morphological data.** (**a**) Data set incorporating all taxa. (**b**) Redundant fossil taxa excluded. Posterior probability support values are indicated at respective nodes.

**
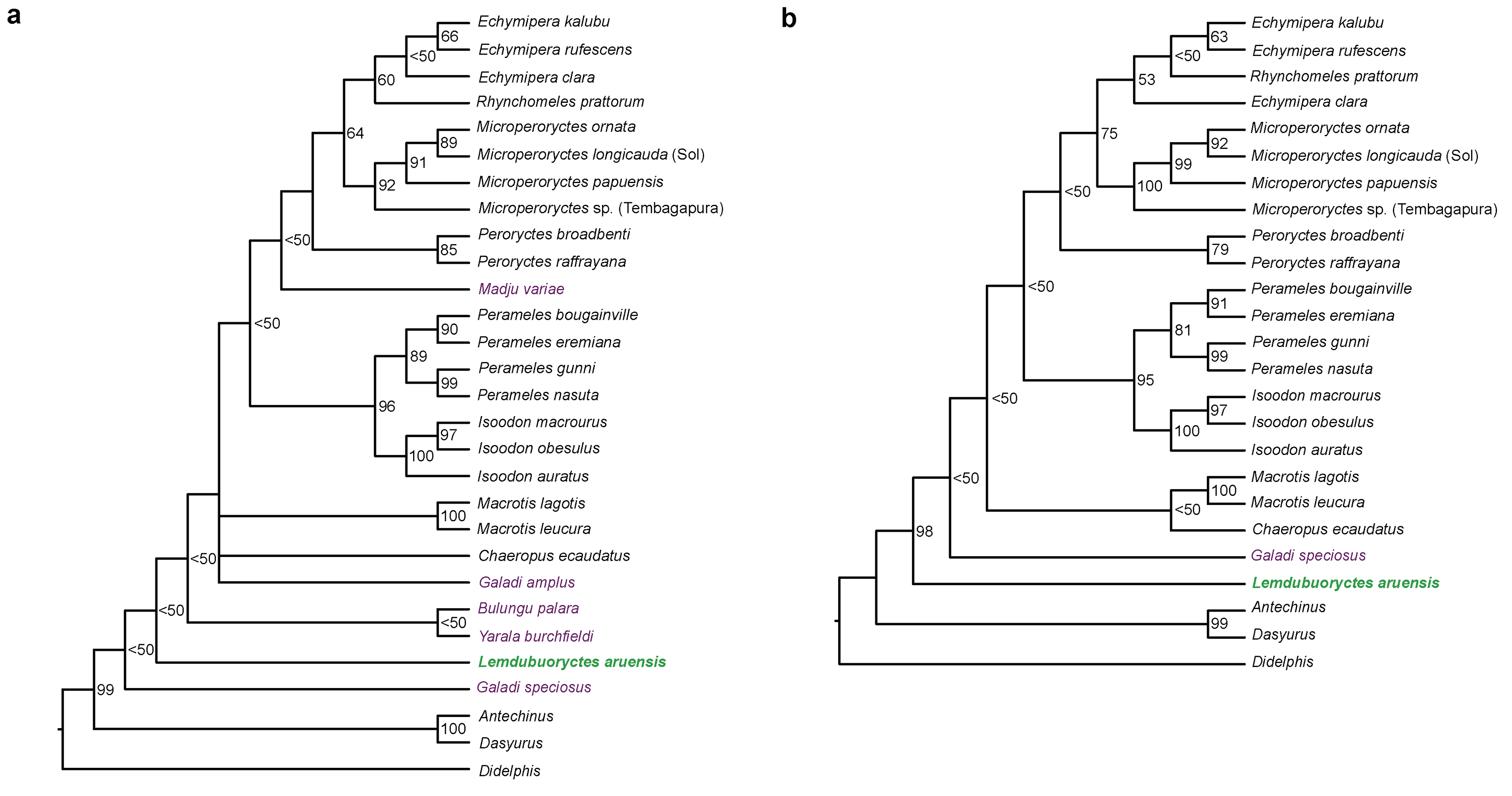
**

**Supplementary Figure S19. Parsimony strict consensus trees of the combined DNA + morphological data.** (**a**) Excluding fossil taxa represented by dental characters only (MPTs = 28, L = 260, CI = 0.4346). (**b**) Excluding fossil taxa represented by dental+cranial characters only (MPTs = 28, L = 260, CI = 0.4346). Bootstrap support values are indicated at all respective nodes.


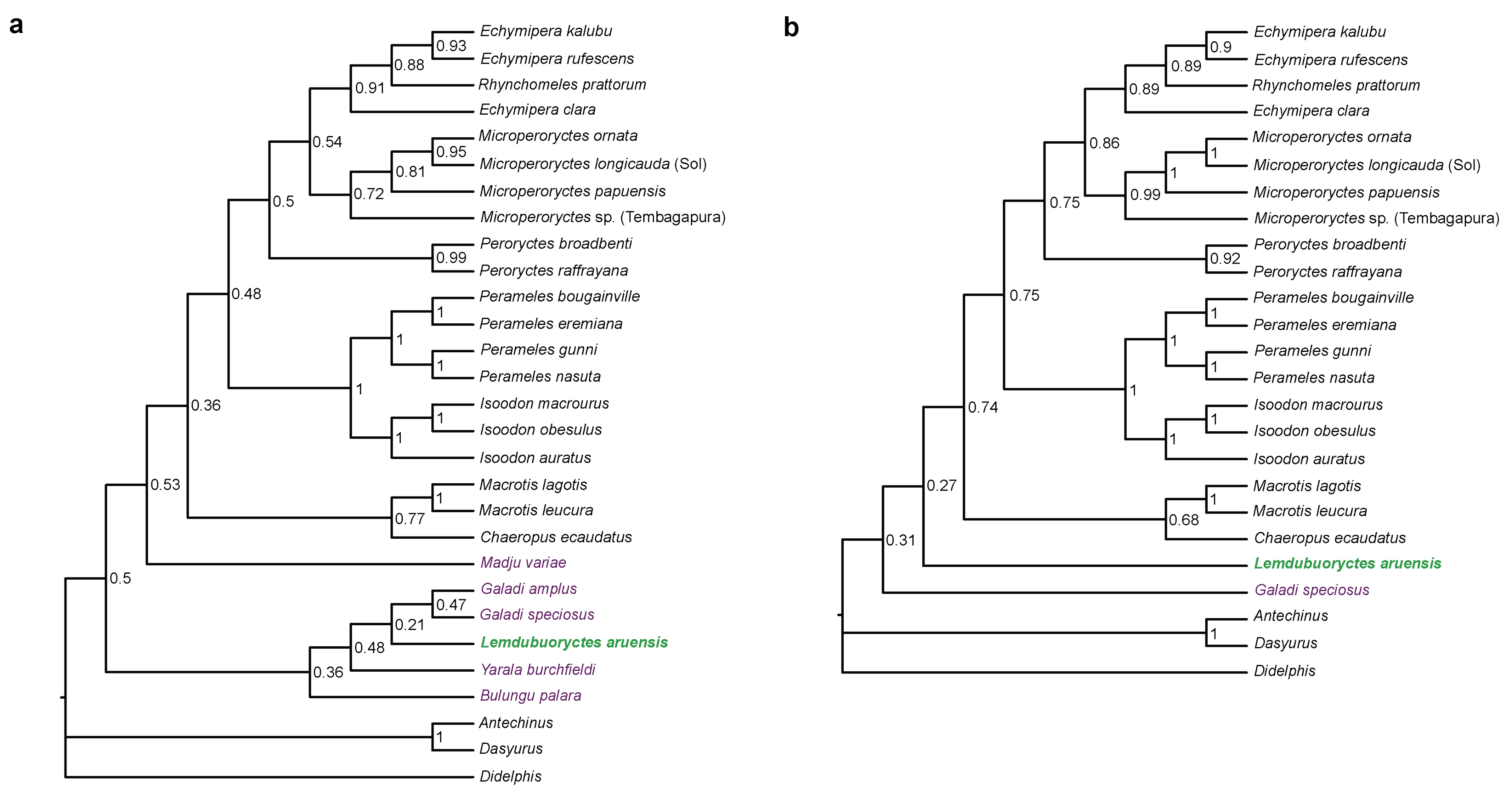


**Supplementary Figure S20. Bayesian consensus trees of combined DNA + morphological data.** (**a**) Excluding fossil taxa represented by dental characters only. (**b**) Excluding fossil taxa represented by dental+cranial characters only. Posterior probability support values are indicated at respective nodes.


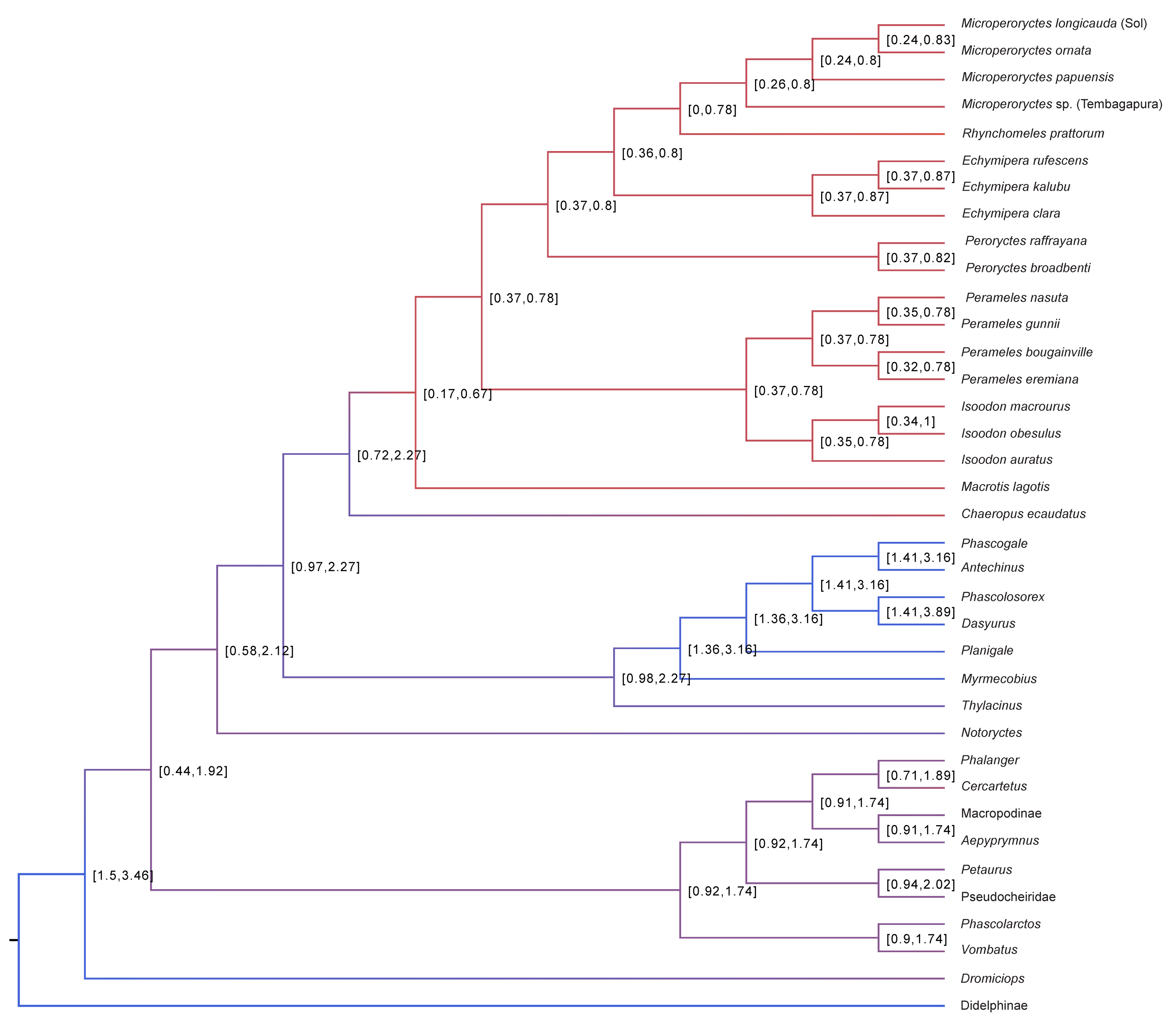


**Supplementary Figure S21. Bayesian inference of random local clocks**^46^ **generated using *BEAST2***^78^**.** Consensus tree incorporating branch colours to indicate different local clocks. Posterior probabilities depict key branch changes over two independent runs. Colour gradient represents decreasing (blue) to increasing (red) rates, which are virtually uniform across Peramelemorphia.

.


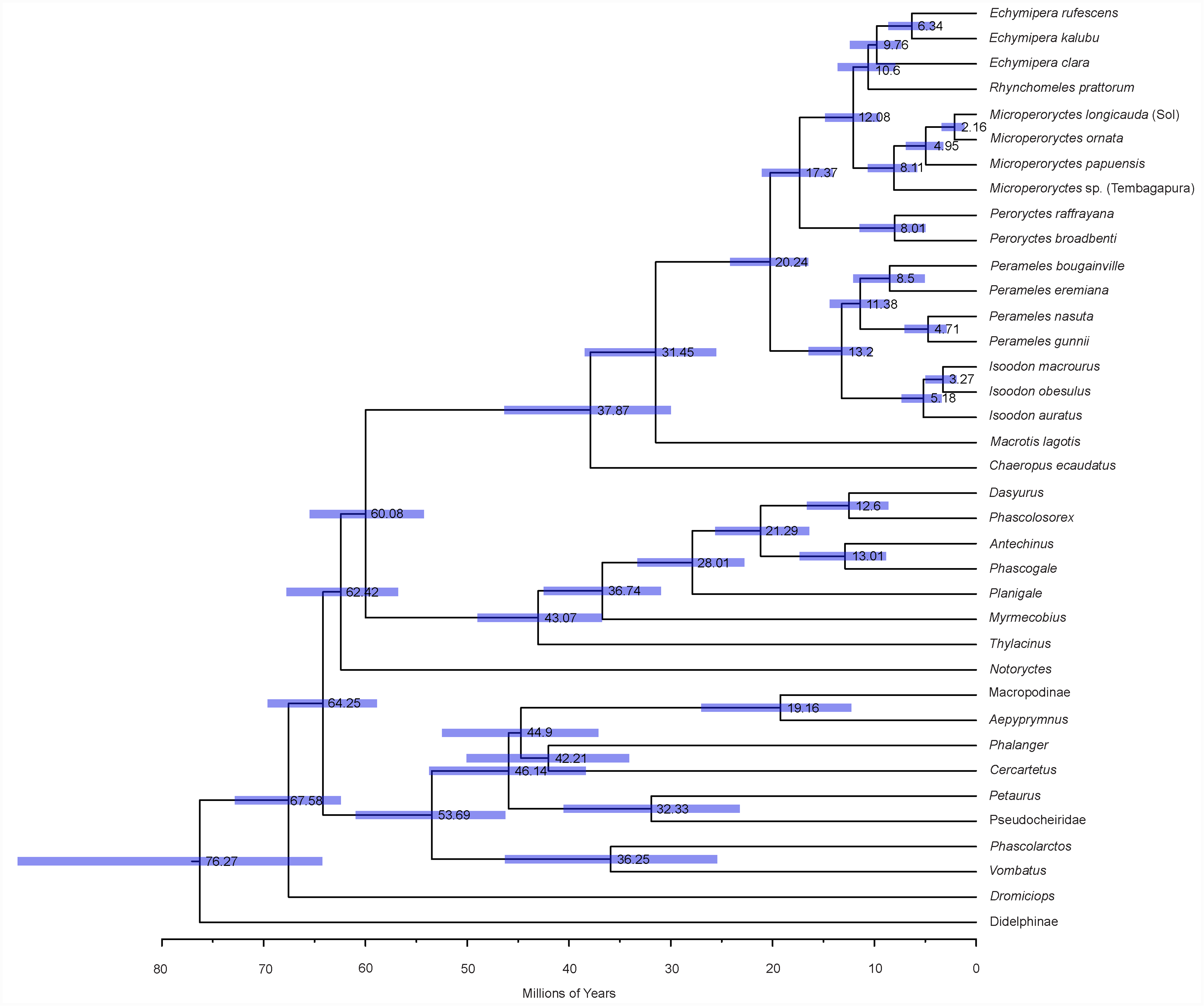


**Supplementary Figure S22. Divergence time-tree generated using *BEAST2***^78^***.*** Peramelemorphian calibration points adjusted to exclude all internal nodes except *Perameles + Isoodon*. Blue boxes represent 95% confidence intervals at each node.


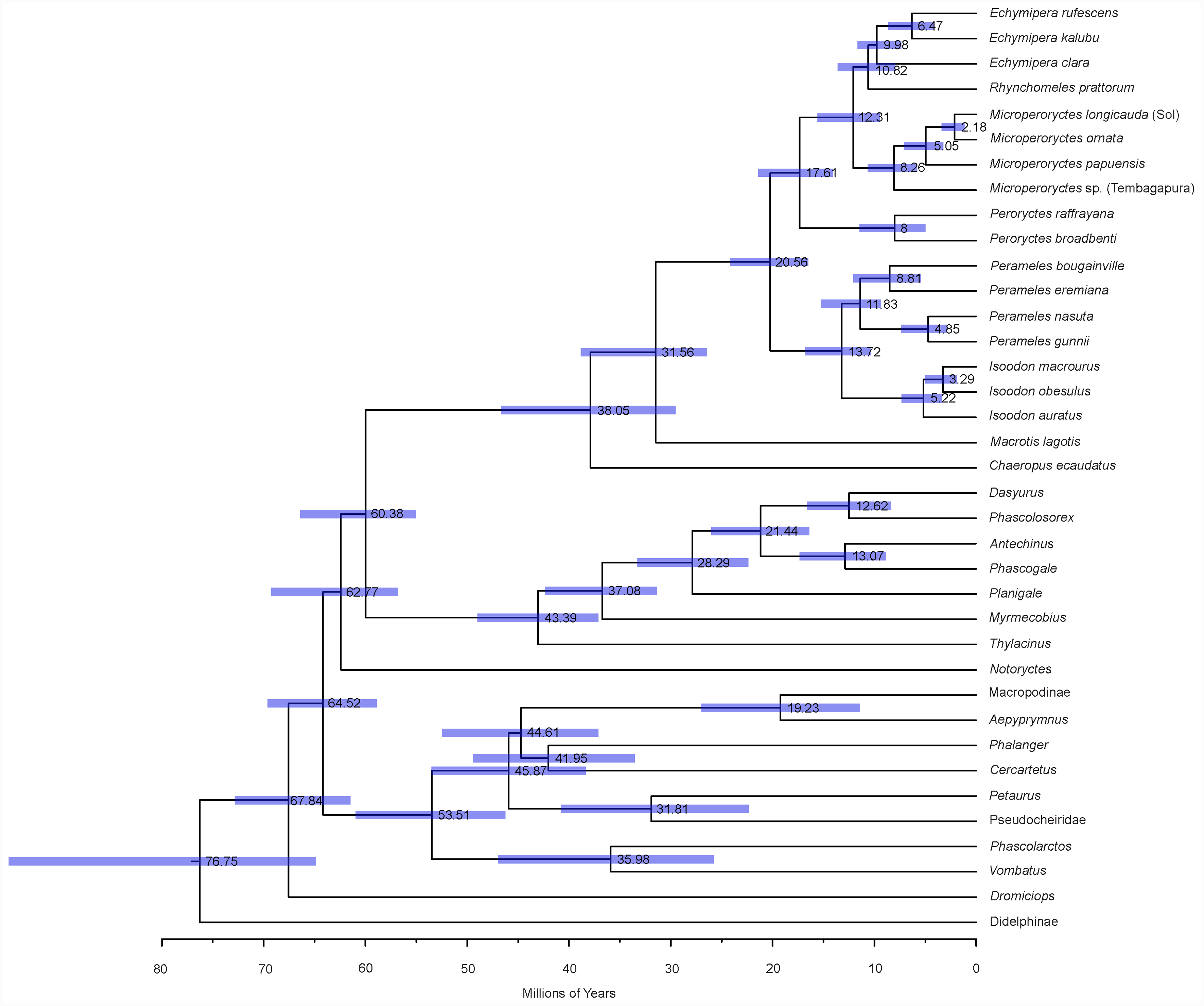


**Supplementary Figure S23. Divergence time-tree generated using *BEAST2***^78^***.*** Peramelemorphian calibration points adjusted to include both Peroryctinae+Echymiperinae and Peramelinae as constrained clades.


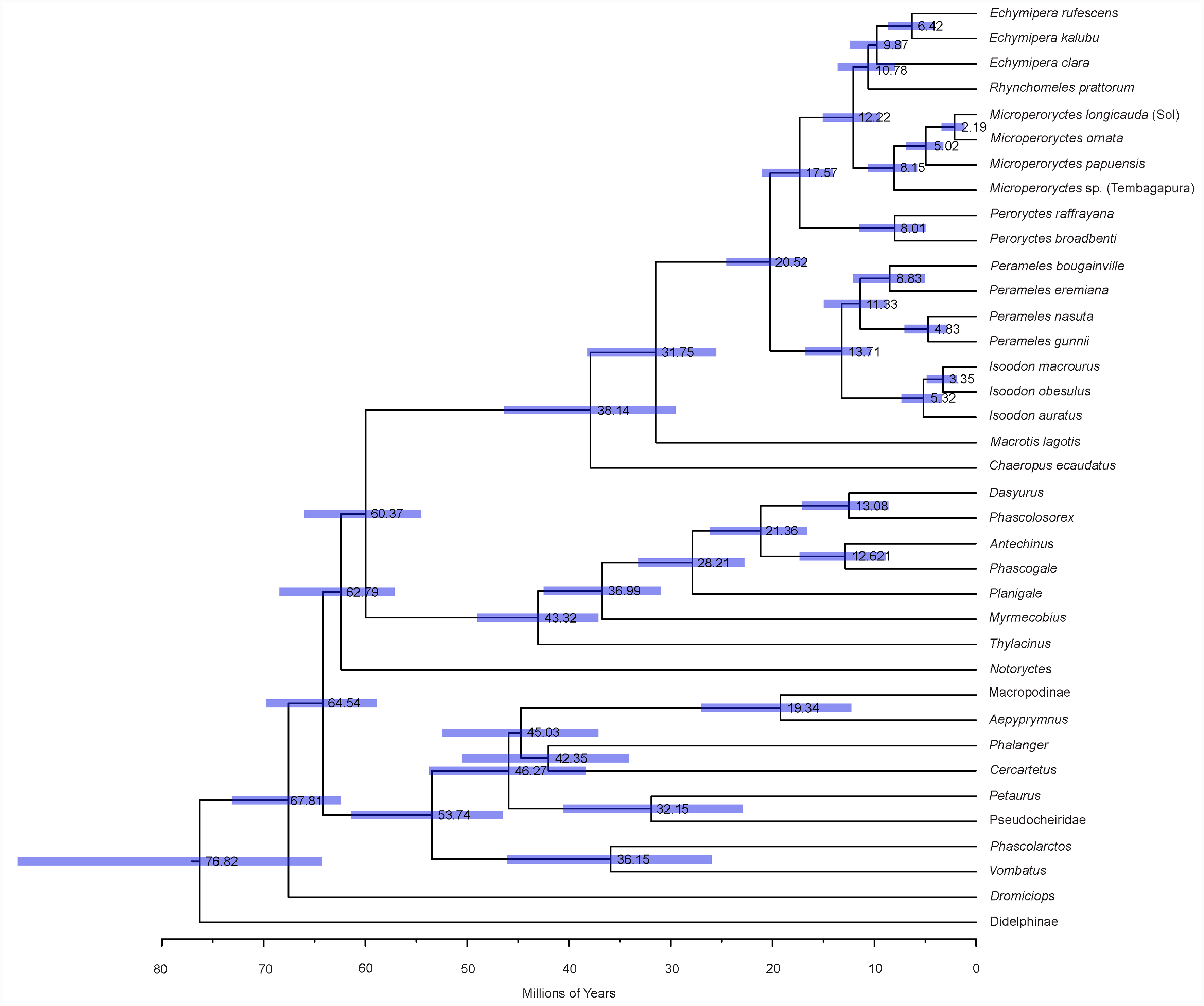


**Supplementary Figure S24. Divergence time-tree generated using *BEAST2***^78^***.*** Peramelemorphian calibration points adjusted to include Peroryctinae+Echymiperinae, Peramelinae and Thylacomyidae as constrained clades.


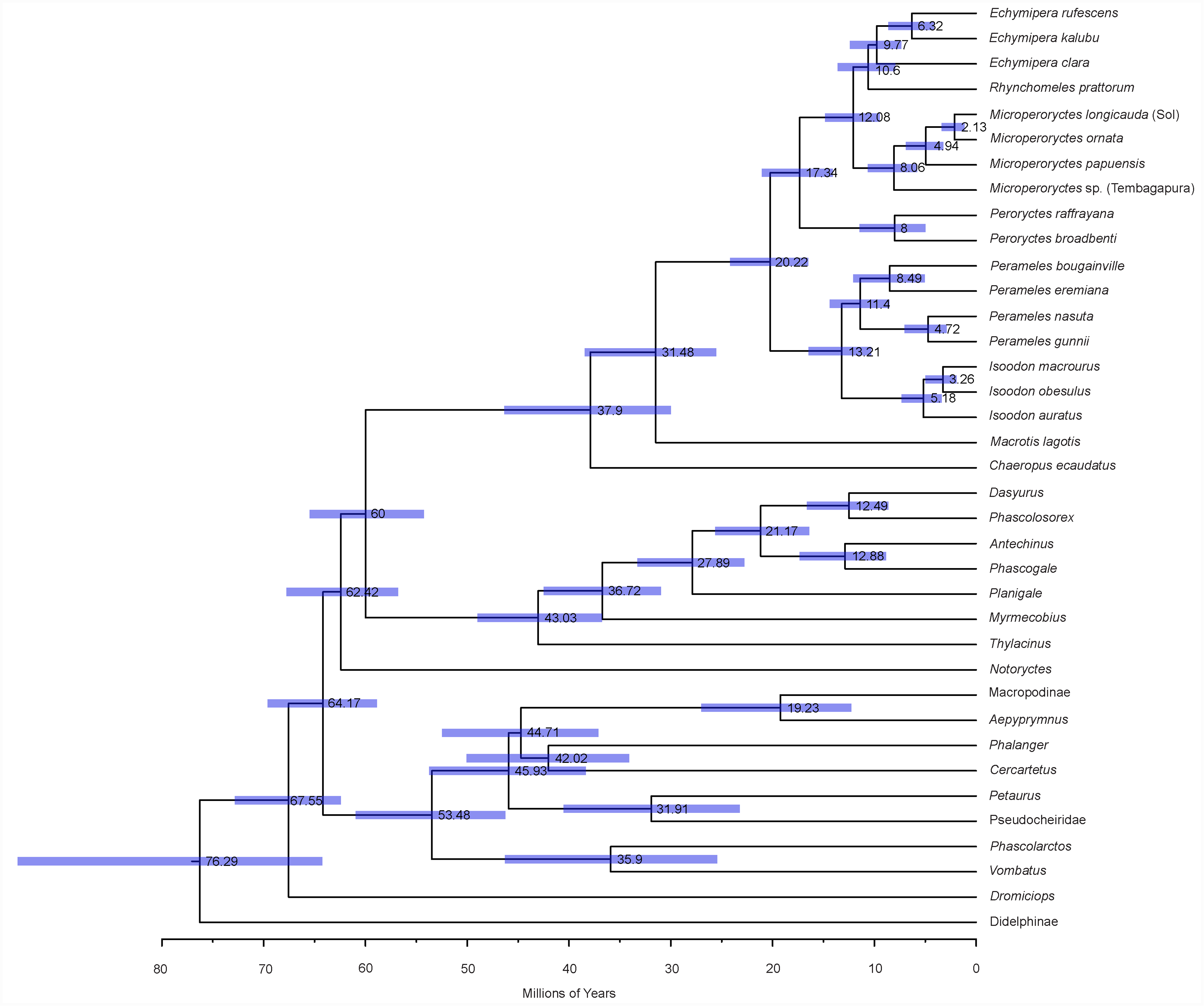


**Supplementary Figure S25. Divergence time-tree generated using *BEAST2***^78^**.** Peramelemorphian calibration points adjusted to include Peroryctinae+Echymiperinae as a constrained clade, but excuding Peramelinae and Thylacomyidae.


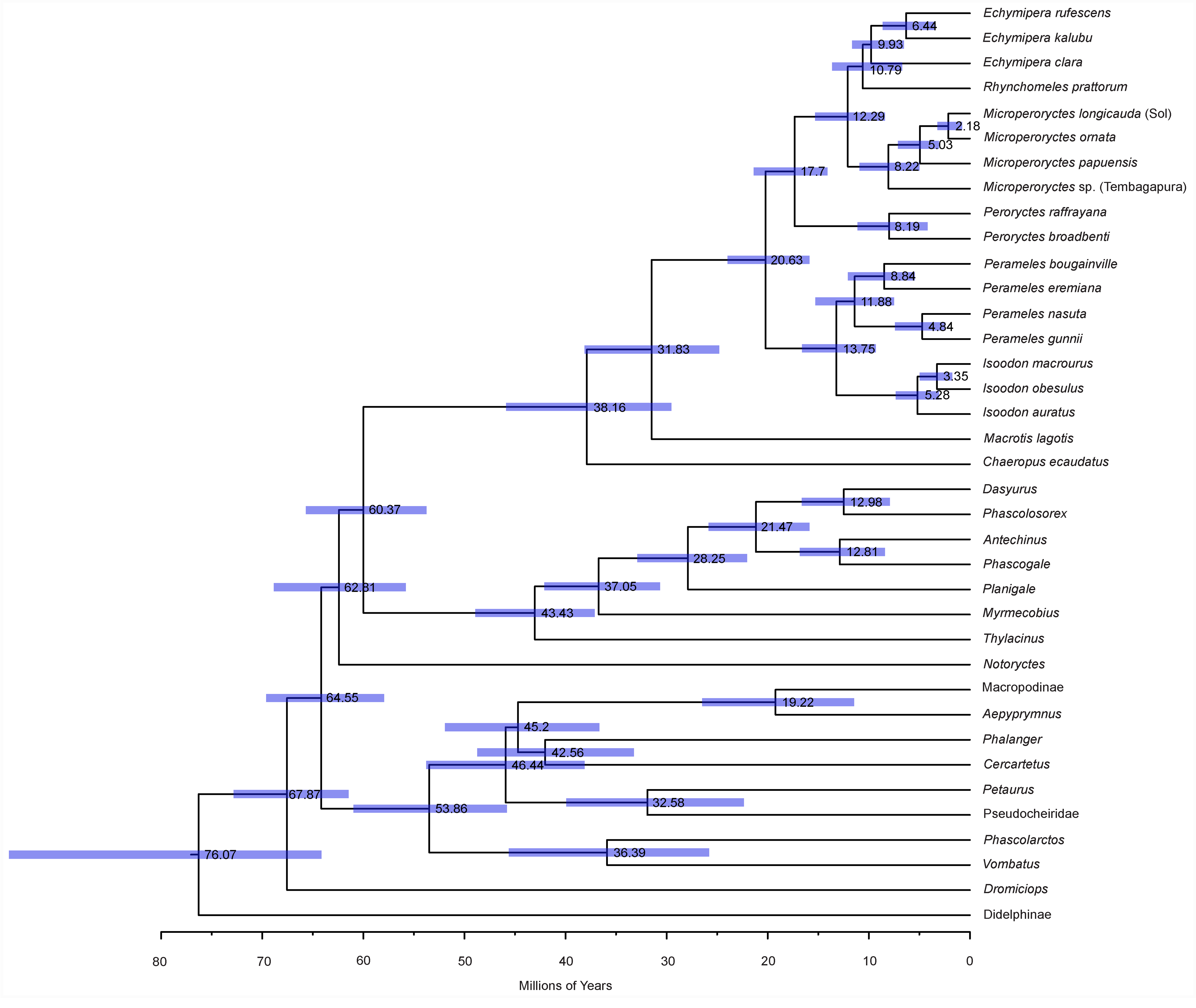


**Supplementary Figure S26. Divergence time-tree generated using *BEAST2***^78^***.*** Peramelemorphian calibration points adjusted to include Peramelinae as a constrained clade, but excluding Peroryctinae+Echymiperinae and Thylacomyidae.


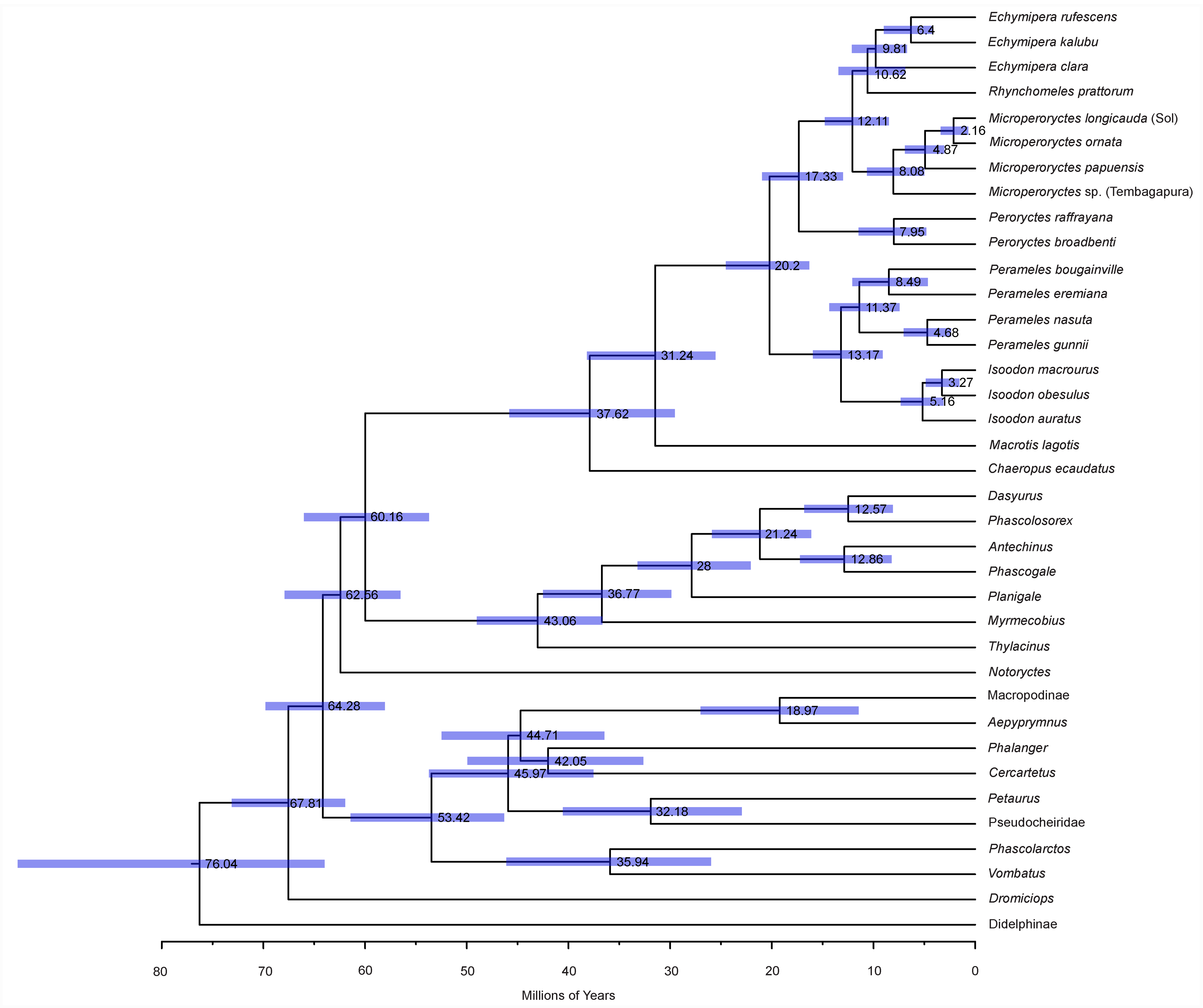


**Supplementary Figure S27. Divergence time-tree generated using *BEAST2***^78^***.*** Peramelemorphian calibration points adjusted to include Thylacomyidae as a constrained clade, but excluding Peroryctinae+Echymiperinae and Peramelinae.


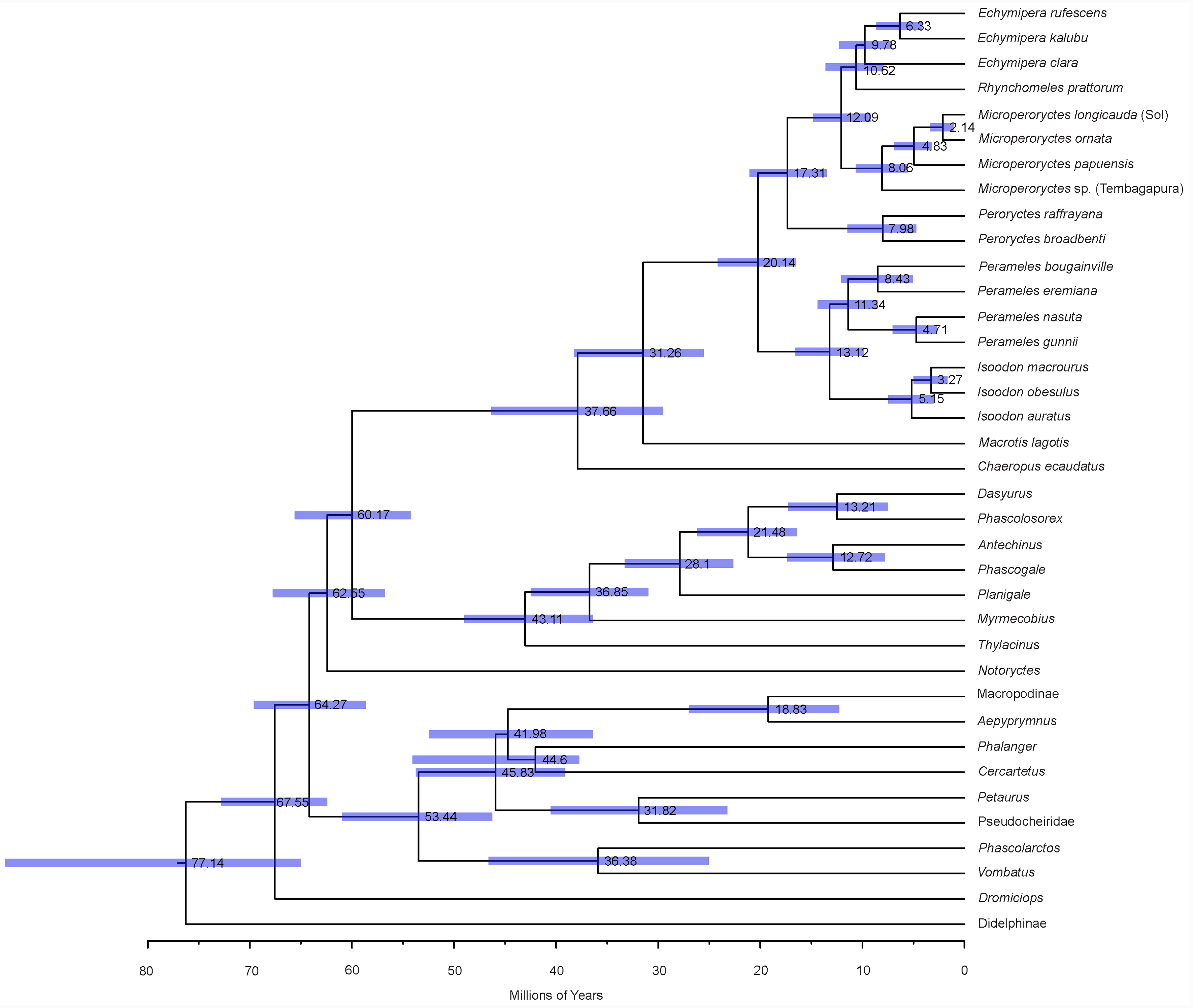


**Supplementary Figure S28. Divergence time-tree generated using *BEAST2***^78^***.*** Peramelemorphian calibration points adjusted to include Chaeropodidae as a constrained clade, but excluding Thylacomyidae, Peroryctinae+Echymiperinae and Peramelinae.


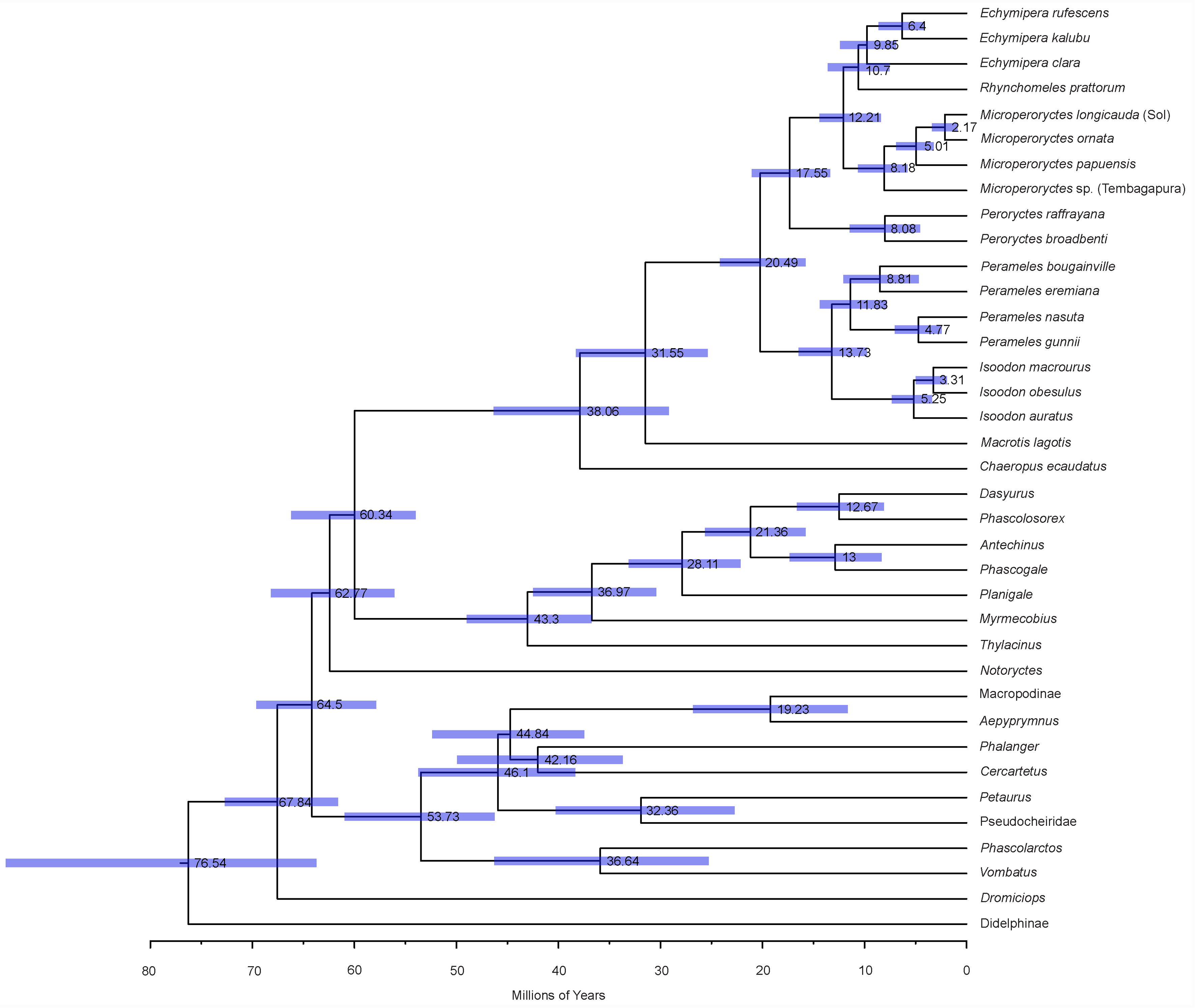


**Supplementary Figure S29. Divergence time-tree generated using *BEAST2***^78^***.*** Peramelemorphian calibration points adjusted to include Peroryctinae+Echymiperinae, Peramelinae, Thylacomyidae and Chaeropodidae as constrained clades.


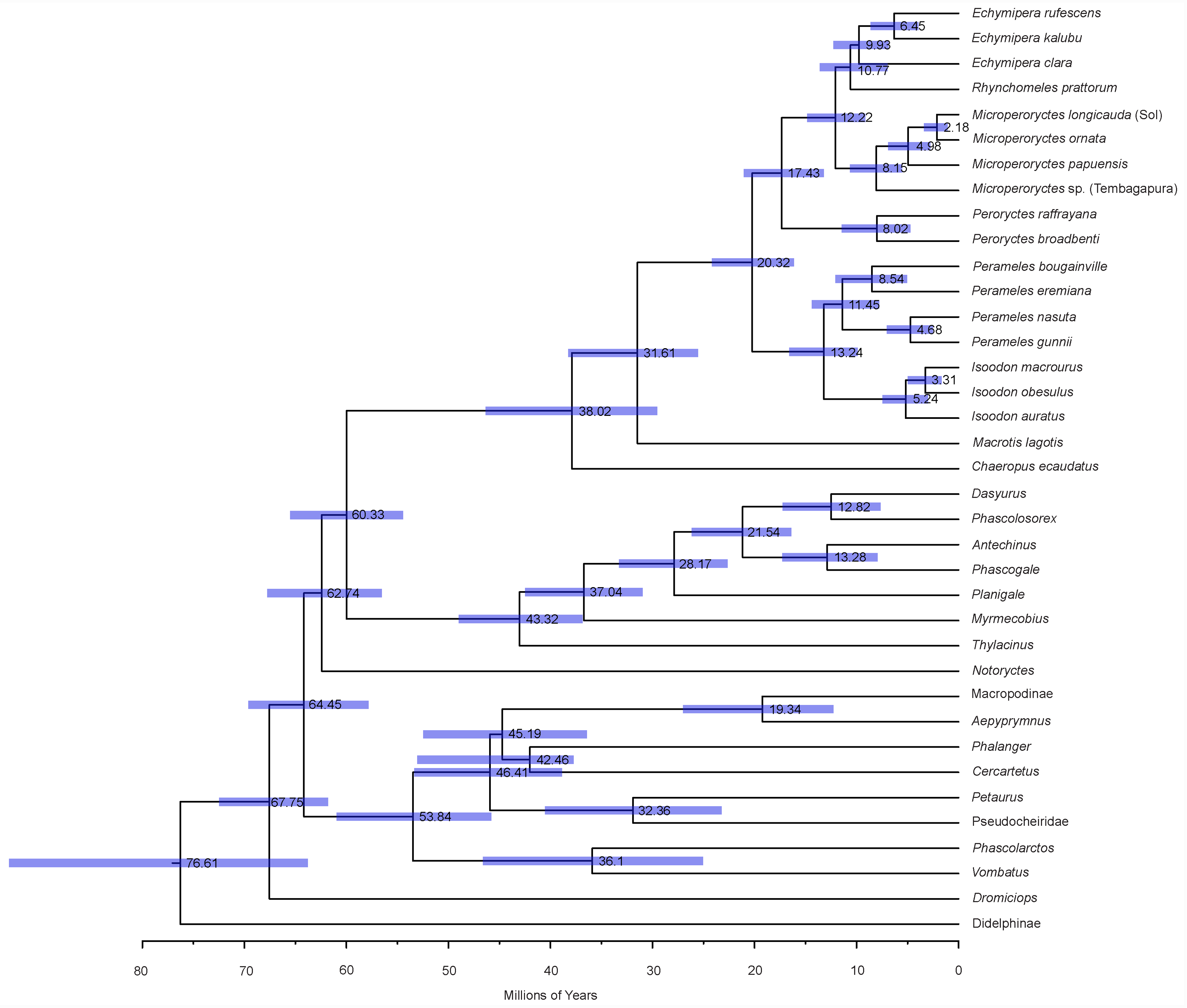


**Supplementary Figure S30. Divergence time-tree generated using *BEAST2***^78^***.*** Stratigraphic bracketing enforced for Dasyuridae (see Supplementary Table S5). Peramelemorphian calibration points exclude all internal nodes except *Perameles + Isoodon*.


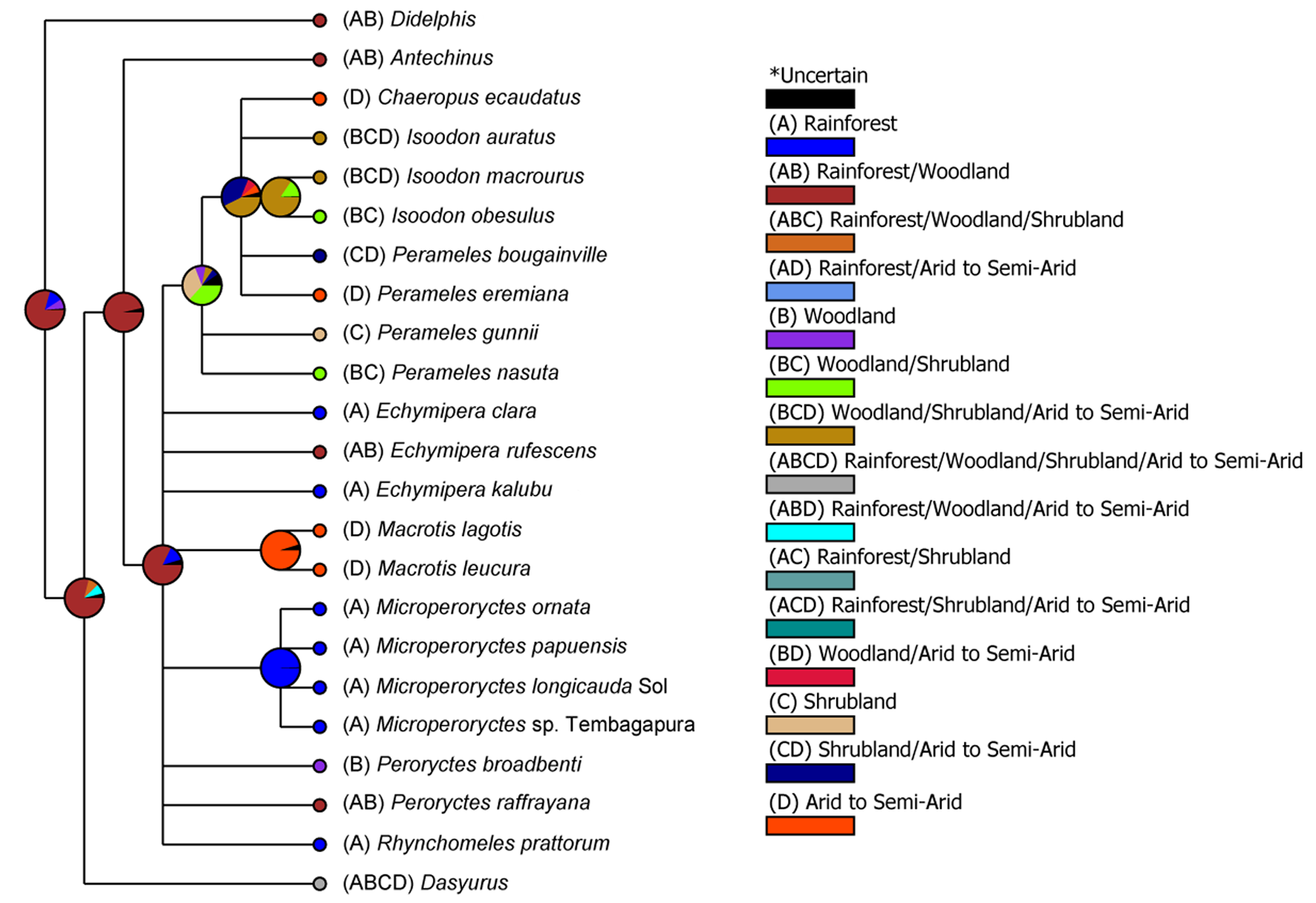


**Supplementary Figure S31. Reconstruction of peramelemorphian ancestral areas output by *RASP*^80^ using a strict consensus morphology-based tree file from *PAUP** 4.0b10^73^.** Bayesian Binary MCMC (BBM) optimization (area distributions and posterior probabilities are listed in Supplementary Table S9).


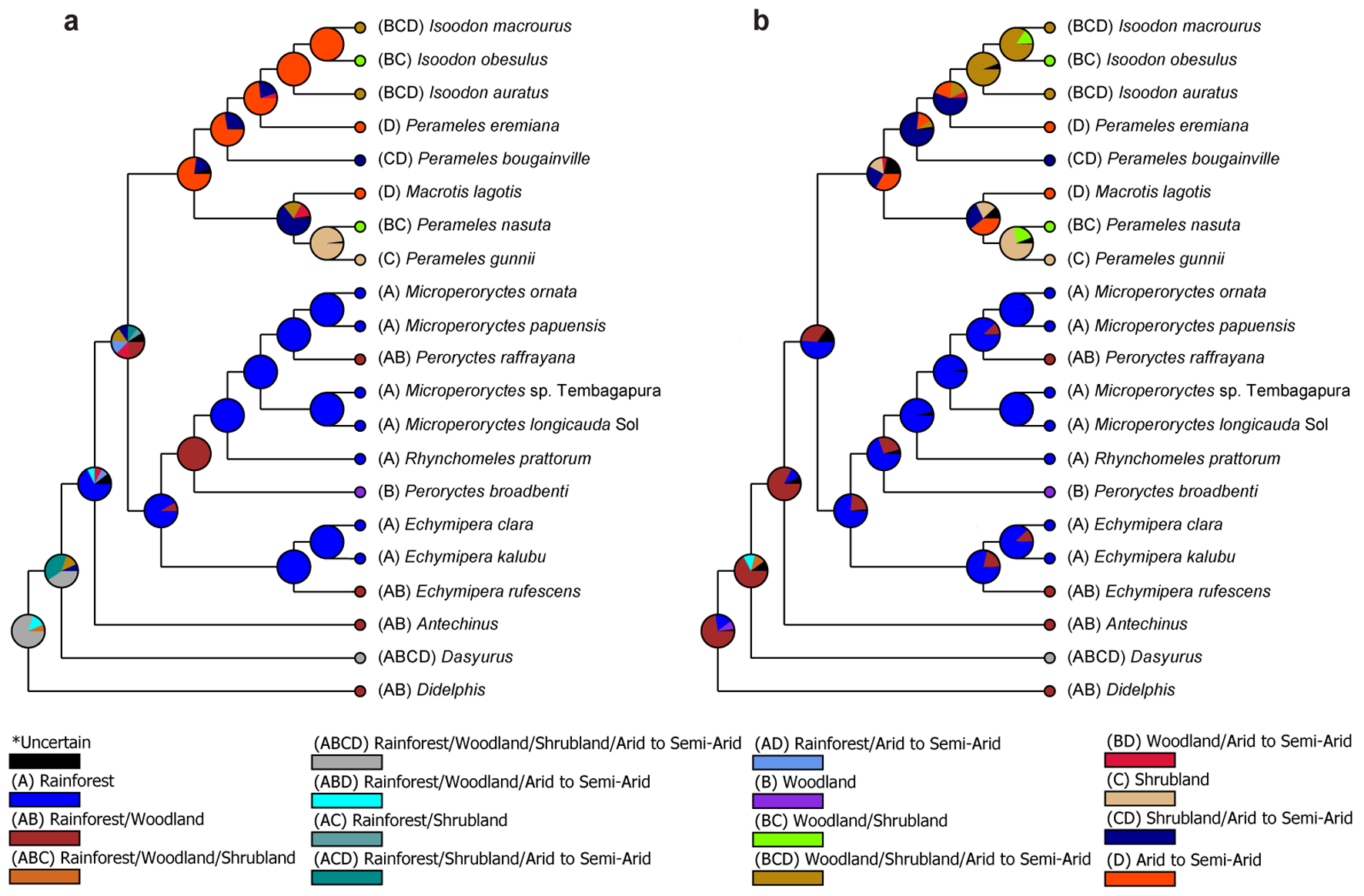


**Supplementary Figure S32. Alternative reconstructions of peramelemorphian ancestral areas output by *RASP*^80^ using morphology-based tree files from *Mr Bayes*^76^.** (**a**) S-DIVA optimization. (**b**) BBM optimization (area distributions and posterior probabilities are listed in Supplementary Table S10).


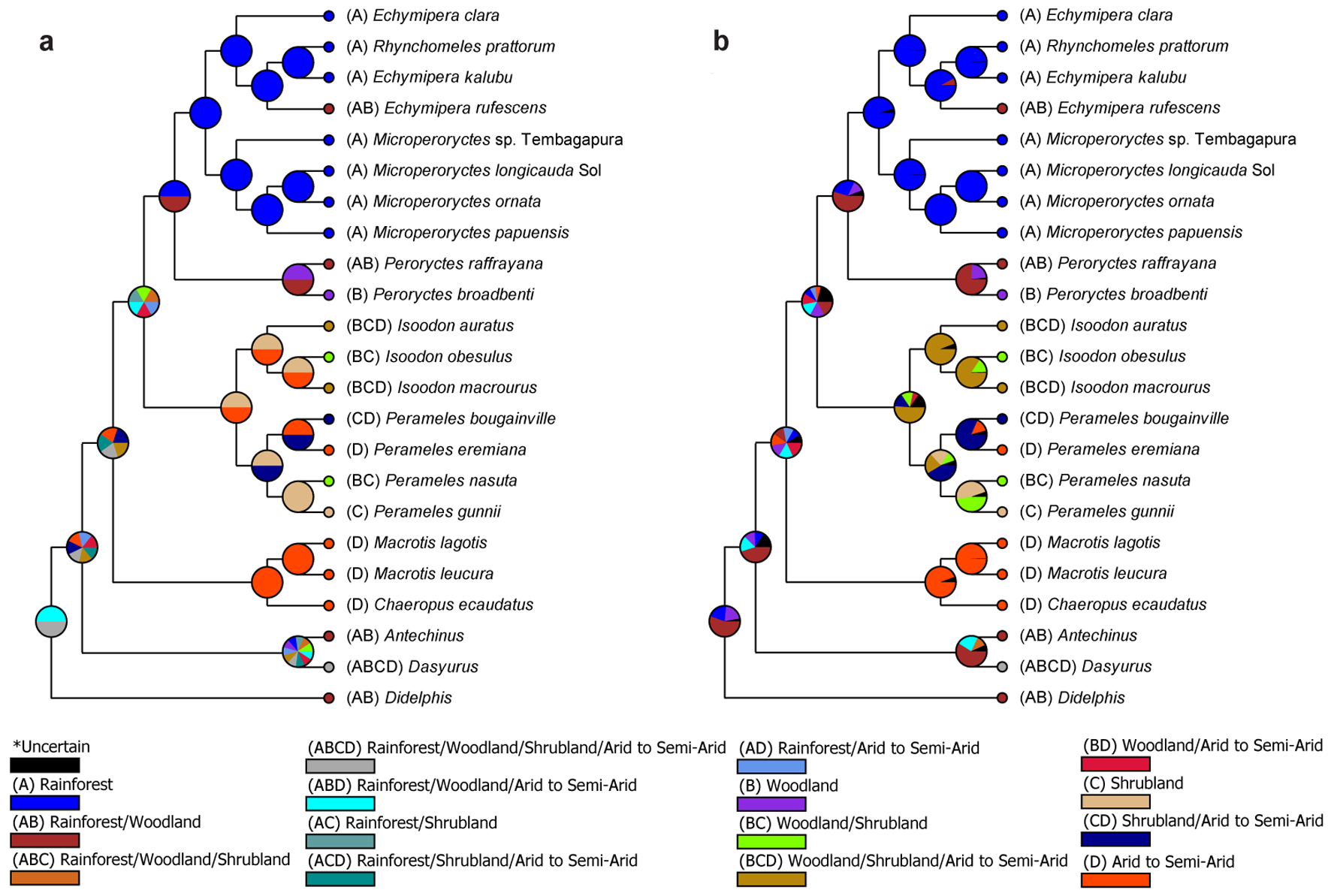


**Supplementary Figure S33. Alternative reconstructions of peramelemorphian ancestral areas output by *RASP80* using combined morphology + DNA sequence trees from *Mr Bayes*^76^.** (**a**) S-DIVA optimization. (**b**) BBM optimization (area distributions and posterior probabilities are listed in Supplementary Table S11).


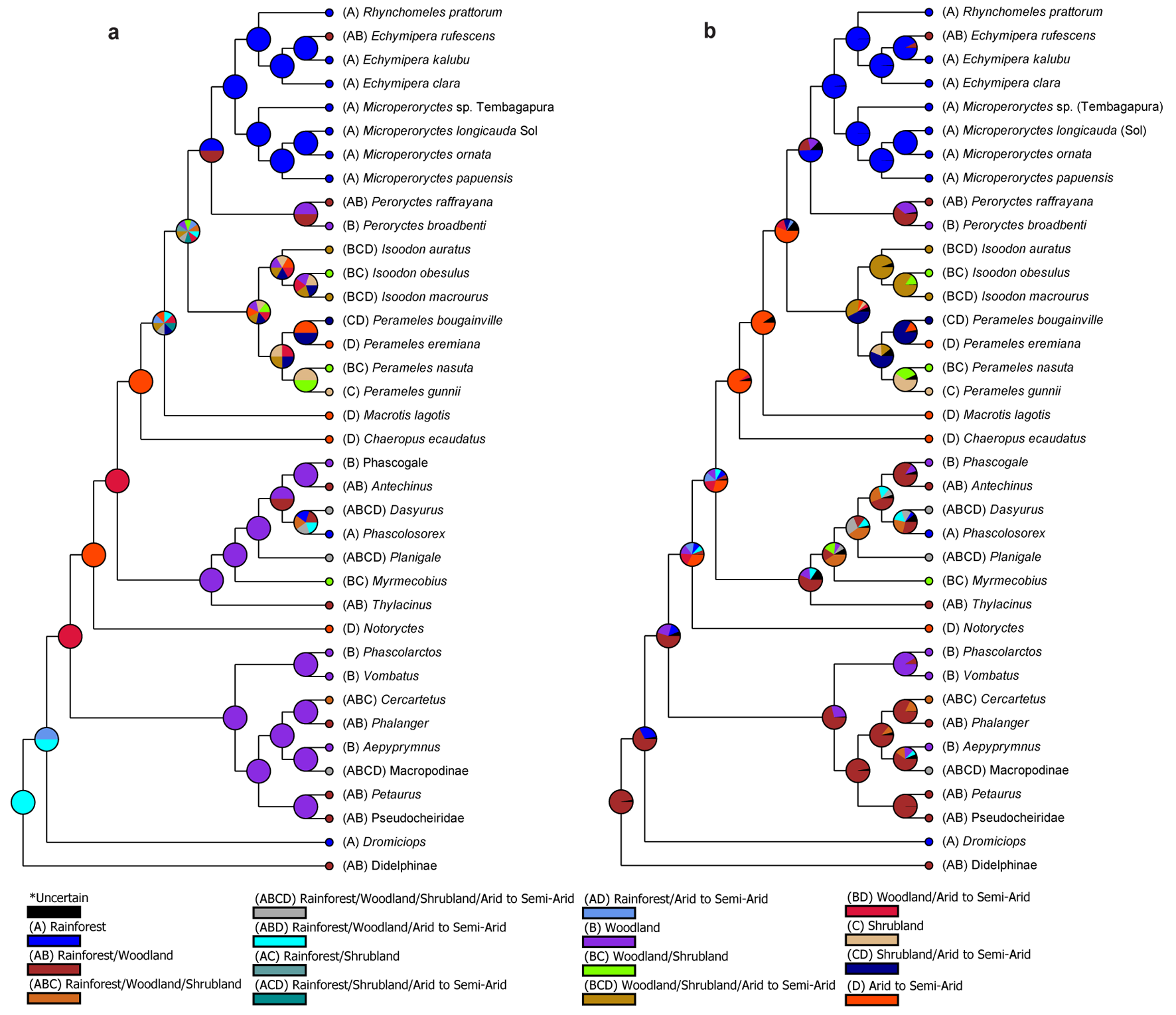


**Supplementary Figure S34. Alternative reconstructions of peramelemorphian ancestral areas output by *RASP*^80^ using DNA sequence tree files from *Mr Bayes*^76^.** (**a**) S-DIVA optimization. (**b**) BBM optimization (area distributions and posterior probabilities are listed in Supplementary Table S12).

**Supplementary Data**

**Annotated morphological character list.** Definitions for most of the craniodental states used in our de novo morphological data set were adapted from published phylogenies and character assessments^11,13–19,26,28,34,37,67^. Referenced character numbers from these works are denoted by the chronologically sequential prefixes GF^11^, MUR^28^, VJA^67^, VJB^26^, AHL^34^, TRA^18^, GUR^16^, TRB^19^, TBC^17^, TRD^13^, TRE^14^ and CHB^15^. In addition, we developed characters 64−93 drawing on information from Stirton^37^ and Szalay^39^. †State morphoclines treated as ordered.

**Craniodental characters adapted from Groves & Flannery^11^.**

1 (GF, Table 2, b12; GUR/TRB60; TRC/TRD/TRE/CHB50). C1 alveolus – (1) situated on the maxillary-premaxillary suture; (0) entirely enclosed within the maxilla. See Voss & Jansa^26^ (p. 166) and Aplin *et al*.^34^ (p. 21) for discussions of this character and its various expressions. Flores *et al*.^98^ reported ontogenetic deviations but these affected only rostral positioning of the maxillary-premaxillary suture relative to the last incisor.

2 (GF, Table 2, b6; AHL8; GUR/TRB67; TRC/TRD/TRE/CHB57). Prominent lateral flanges on the premaxillary contribution to the bony nasal opening – (1) present; (0) absent. Groves & Flannery^11^ (p. 6) alternatively described these structures as ‘lateral wings’. Aplin *et al*.^34^ (p. 22) and Travouillon *et al*.^19^ (supplementary data 1) also used the term ‘narial processes’.

3 (GF, Table 1, b2). Anterior root of the zygomatic arch – (1) at alveolar level; (0) above alveolar level.

4 (GF, Table 1, a1). Orbital region of the skull – (1) flattened dorsally; (0) convex dorsally.

5 (GF, Table 1, a2). Antorbital portion of the zygomatic arch – (1) with tapered anterior transition into the antorbital region; (0) strongly arched anterolaterally around the orbit.

6 (GF, Table 2, a12; TRC/TRD/TRE/CHB66). Diastema between i3 and c1 – (1) present; (0) absent. Travouillon *et al*.^17^ created additional characters to recognize diastemata between both the upper and lower canines, P1/p1 (TRC/TRD/TRE/CHB69, TRC/TRD/TRE/CHB75), and P1/p1 versus P2/p2 (TRC/TRD/TRE/CHB72, TRC/TRD/TRE/CHB79). However, diastemata formation within the upper jaw is ontogenetically coupled to proportional elongation of the snout in peramelemorphians^98^, and thus forms an interdependent continuum. In addition, Voss & Jansa^26^ found the expression of the P1/P2 diastemata to be intraspecifically variable in didelphids. We therefore restricted our description of this trait to the unambiguous separation (or not) of i3 versus c1, and likewise avoided subjective estimates of relative length (e.g. GF Table 2, a13 and b13)^11^.

7 (GF, Table 3, a14; GUR74; TRC/TRD/TRE/CHB63). Metacone on M1–3 – (1) positioned towards the lingual edge far out of alignment with the paracone; (0) positioned approximately posterior to the paracone. Gurovich *et al*.^16^ (Appendix A) described the distinctive morphology of *Macrotis* spp. in terms of the metacone having ‘shifted lingually’. However, Groves & Flannery^11^ (p. 8) envisaged the metacone as having undergone in situ ‘expansion’. Because these state acquisition mechanisms are equally speculative, we followed Travouillon *et al*.^17^ in using metacone placement relative to the paracone as a more stable point of reference.

**Craniodental characters adapted from Muirhead^28^.**

8 (MUR1+2; TRA/GUR/TRB/TRC/TRD/TRE/CHB1–3). Upper incisor number and shape – (2) I5 lost; (1) I5 ‘canine-like’ and separated from I4 by a diastema; (0) I5 directly adjacent to I4 and of similar morphology. Muirhead^28^ originally scored these state manifestations as two linked characters (MUR1+2) relating to I5 loss and shape. Travouillon *et al*.^18^ alternatively derived three discrete characters to describe these same features (TRA/GUR/TRB/TRC/TRD/TRE/CHB1–3). In contrast, we employed a multistate scoring to eliminate overweighting and possible interdependence. Polarity was uncertain therefore we treat all states as unordered.

9 (MUR3; TRA/GUR/TRB/TRC/TRD/TRE/CHB4+5+74+153). P3 occlusal shape – (2) P3 absent; (1) P3 crown base ‘triangular’ with expanded conical primary cusp [incorporating MUR3 state 3 only]; (0) P3 crown base narrow and ‘blade-like’ with linear cusp arrangement [incorporating MUR3 states 0–2]. We considered the multistate subdivisions described by Muirhead^28^, and later studies^13–19^ to be subjective. These were therefore redefined using a simple discriminative coding of P3 absence versus organisation of the central cusp. Travouillon *et al*.^18^ also introduced relative development of the lingual shelf to differentiate P3 morphologies within peramelemorphians (TRA/GUR/TRB/TRC/TRD/TRE/CHB4). However, we observed that shelf absence versus presence is interdependent with overall P3 shape (although this was modified in TRC/TRD/TRE/CHB4) and length (TRC/TRD/TRE/CHB74) in their state scores. Their descriptive terms ‘weakly’ or ‘well developed’ are also subjective. Our solution thus subsumed these derivative characters, and retained all states as unordered because of uncertain polarity. Note that form of the P3 was found to be unaffected by size-related sexual dimorphism^34^.

10 (MUR4; TRA/GUR/TRB/TRC/TRD/TRE/CHB6). Stylar cusp D on the M1 – (1) conical and without stylar crest; (0) continuous with stylar crest connecting to the metastylar tip. Travouillon *et al*.^17^ introduced a third state ‘StD is a conical cusp not connected to the stylar crest running to the metastylar tip’. However, we considered this developmentally equivalent to primary alteration of the stylar cusp as described and coded here. Travouillon *et al*.^17^ also included two additional characters with identical state designations for M2 (TRC/TRD/TRE/CHB98) and M3 (TRC/TRD/TRE/CHB109). Nevertheless, we observed contrasting scores and character redundancy relative to our taxon sample and thus excluded them from our analysis.

11 (MUR5; TRA/GUR/TRB/TRC/TRD/TRE/CHB7+124). Anterior cingulum on the M1 – (1) present; (0) absent. Travouillon *et al*.^17^ inflated the number of states for this character by quantifying incremental lingual extension of the anterior cingulum. We alternatively reduced our state options to two because extent of the cingulum was found to be intraspecifically variable within our sample. Moreover, determination of the cingulum terminus required qualitative estimation and would thus introduce arbitrary state coding.

12 (MUR6; TRA/GUR/TRB/TRC/TRD/TRE/CHB8+93+115). Anterior cingulum on M2–4 – (1) present and pronounced; (0) vestigial or absent. See previous comment.

13 (MUR7; TRA/GUR/TRB/TRC/TRD/TRE/CHB9). Orientation of the M1 preparacrista – (1) preparacrista posterobucally oriented parallel to postparacrista; (0) preparacrista anterobucally orientated. Travouillon *et al*.^17^ added additional ‘blade-like’ versus ‘not blade-like’ categories, which were further modified to incorporate degree of angling, estimated position relative to stylar cusp B, and ‘reconnection’ with the postparacrista^16^. These states all capture shape variation in the anterior margin of the stylar shelf. Therefore we employed a similar but simpler binary state model to accommodate the morphological range observed in our sample.

14 (MUR8; TRA/GUR/TRB10; TRC/TRD/TRE/CHB97+108+118). Extent of M2–4 preparacrista – (1) preparacrista connects to parastylar tip; (0) preparacrista connects to stylar cusp B. We retained this independent character because it describes the extent of the preparacrista as opposed to its extrapolated angle; it also specifically focused upon the posterior molars in which the trigonid cusps have a distinctive arrangement (see character 16 below). Note, however, that the scores presented by Muirhead^28^, Travouillon *et al*.^18^ and Travouillon *et al*.^19^, are incongruous with our observations. Demarcation of subsidiary states within these descriptions hinges upon the contact of the preparacrista with stylar cusp B versus the stylar margin. This certainly occurs on the M2 of some *Microperoryctes* and *Peroryctes* specimens, but is otherwise absent in all other taxa including *Macrotis* (scored ‘1’ instead of ‘0’ because the preparacrista extends towards the stylar edge away from stylar cusp B). Independent reduction of the stylar cusp B contact on M2 and M3 cannot be conclusively demonstrated, thus separation of discrete characters for these molars^17^ is not followed herein.

15 (MUR9; TRA/GUR/TRB11; TRC/TRD/TRE/CHB88+99). Posterior cingulum on M1–2 – (1) present; (0) absent. Definition of an ‘incomplete cingulum’^16,18,19,28^ is ambiguous. Likewise, we considered relative extent of the cingulum on M1 versus M2 to reflect dependent polymorphisms rather than independent states. Travouillon *et al*.^17^ removed the term ‘incomplete cingulum’ from their description but introduced a ‘present and wide’ category for M1–3, which we found impossible to discriminate; distribution of states ‘1’ and ‘2’ in Travouillon *et al*.^17^ are also almost identical.

16 (MUR10; TRA/GUR/TRB12; TRC/TRD/TRE/CHB10). Posterior cingulum on the M3 – (1) present; (0) absent. See previous comment. The condition on M3 is treated as independent of that on the highly modified M4.

17 (MUR11; TRA/GUR13; TRB13+74; TRC/TRD/TRE/CHB90+101+111). Metaconule (metaconular hypocone) – (1) developed into a prominent, clearly defined cusp; (0) vestigial or absent. States ‘0’ and ‘1’ of Muirhead^28^ incorporated a ‘small’ size category, with ‘2’ being ‘almost as large’. Travouillon *et al*.^17^, Gurovich *et al*.^16^ and Travouillon *et al*.^19^ further employed ‘miniscule’, ‘small’, ‘almost the same size’, and ‘equal to, or slightly larger’ to identify their states 0–3. We could not differentiate these conditions objectively, and therefore rationalised our description into a binary coding. Travouillon *et al*.^17^ alternatively divided the condition of the metaconule on M1–3 into three characters (TRC/TRD/TRE/CHB90+101+111) with identical states describing the relative distance (either ‘directly positioned’, or separated by a ‘small’ or ‘larger’ shelf) from the metacone. We rejected this approach because it did not capture comparative development of the metaconule, nor could it assume developmentally independent regression of the cusp on each successive molar^99^.

18 (MUR12; TRA/GUR/TRB14; TRC/TRD/TRE/CHB92+104+114). Stylar cusp E – (1) lost as distinct cusp; (0) well developed on at least one molar. Previous works^16,18,19,28^ have differentiated the presence of stylar cusp E on each molar as a discrete state, which Travouillon *et al*.^17^ subsequently elevated to separate characters. Loss of stylar cusp E cannot be unambiguously decoupled between individual teeth^99^. Furthermore, variability within the stylar shelf, which is free of occlusal constraints, would seem to favour atavisms (e.g. spurs, cuspules) and differential wear. We therefore scored expression of stylar cusp E unanimously along the entire molar row.

19 (MUR13; TRA/GUR/TRB15+16; TRC/TRD/TRE/CHB11+12). Centrocrista on the upper molars – (1) incomplete, thus ‘breeching’ the ectoloph; (0) complete, thus does not ‘breech’ the ectoloph on at least one tooth. Travouillon *et al*.^18^, Gurovich *et al*.^16^ and Travouillon *et al*.^19^ introduced various state subdivisions in an attempt to differentiate the polymorphic condition of an intermittently ‘breeched’ ectoloph (e.g. *Peroryctes broadbenti*^34^: p. 21, figure 10A). These included ‘more’ buccal versus lingual positioning of the postparacrista-premetacrista contact, and its relative expression on M1–2 versus M3 (sometimes distinguished as separate characters^13–17,19^). Because loss of the postparacrista-premetacrista contact is clearly correlated between teeth^26,67,100^, and relative buccal or lingual positioning of the contact is subjective, we have chosen to limit our definitions to the simple presence or absence of the ‘breeched’ ectoloph^28^, with recognition of polymorphisms rather than selective weighting via multiple dependent characters and/or developmentally linked^99^ incremental morphoclines^63^. In addition, we considered relative connection of stylar cusps B and D on M1–3 via a ‘series of crests’, discriminated into a character series by Travouillon *et al*. (TRC/TRD/TRE/CHB87+96+107)^17^, to be influenced by the condition of the centrocrista and therefore interdependent.

20 (MUR14; TRA16; GUR/TRB17; TRC/TRD/TRE/CHB13). Lobation of the i3 – (1) bilobed; (0) not bilobed. We eliminated the ambiguous ‘small’ versus ‘large’ state categories designated for the posterior i3 cusp by Travouillon *et al*.^17^.

21 (MUR15; TRA17; GUR/TRB18; TRC/TRD/TRE/CHB14+80+82+154). Morphology of the p3 – (2) absent; (1) crown base broad with conical central cusp; (0) crown base narrow with ‘blade-like’ central cusp. Travouillon *et al*.^17^ created a second interlinked character (TRC/TRD81) describing the development of the ‘anterior cusp’ on the p3. Modification of the p3 towards the derived conical form invariably results in reduction of the anterior cusp in peramelemorphians. Character unordered because of indeterminate polarity.

22 (MUR16; TRA18; GUR/TRB19). Premolar roots – (1) covered by buccal expansion of the enamel layer; (0) buccal enamel restricted to crown (root exposed). Definition of the ‘raised’ premolar condition described by Muirhead^28^ and Travouillon *et al*.^18^ were incongruous because exposure of the root above the alveolar rim occurred in our outgroup taxa. However, buccal expansion of the enamel layer over the root is clearly identifiable, and presumably reflects an increase in the functional life of the tooth via prolonged wear into the base of the crown.

23 (MUR17; TRA19; GUR/TRB20; TRC/TRD/TRE/CHB126+127+133+138+143). Trigonid cusp disposition – (2) paraconid lost on m1–3; (1) paraconid and metaconid adjacent on at least m3–4 (narrow trigonid); (0) cusps widely separated on m3–4 (broad trigonid). The differentiation of cusp positioning on the anterior versus posterior molars described by Muirhead^28^ and Travouillon *et al*.^18^ were subjective. These cusps are demonstrably narrower on the m1 relative to all subsequent molars in our sampled taxa. Nevertheless, cusp positioning on m2–4 was found to be variable. We therefore restricted this character focus to the rear section of the tooth row. In addition, we specifically avoided using the interdependent character sets of Travouillon *et al*.^17^, because these specified incremental length estimates for different states and reiterated paraconid positioning. Character was treated as unordered.

24 (MUR 18; TRA20; GUR/TRB21; TRC/TRD/TRE/CHB132+137+142+151). Hypoconulid elevation on m1–3 – (1) hypoconulid reduced and situated at or beneath the level of the talonid basin; (0) hypoconulid prominent and elevated high above the talonid basin. We follow Travouillon *et al*.^17^ in assessing hypoconulid development independent of the posthypocristid. We also found lingual displacement of the hypoconulid relative to the entoconid to be minimal when compared through our selected outgroups. Our character definition was therefore redefined to score only elevation (vis-á-vis size^17^) of the hypoconulid relative to the talonid basin. Note that reduction of the talonid on the m4 usually results in loss of an obvious hypoconulid.

25 (MUR19; TRA21+22; GUR/TRB22+23; TRC/TRD/TRE/CHB15+16+128+144+145+146+147+

150). Entoconid shape on m1–3 – (1) conical, forming a distinct cusp; (0) ‘blade-like’ and may extend with the preentocristid/postentocristid. Muirhead’s^28^ ‘blade-like’ versus ‘low’ shape categories are difficult to distinguish. We therefore altered our state definitions to accommodate only the formation (or not) of a conical cusp (the entoconid is indistinct on m4 because of talonid reduction). Travouillon *et al*.^18^ and Travouillon *et al*.^17^ differentiated the presence of the preentocristid and postentocristid, together with their orientations, as separate characters for each molar. We alternatively regard these traits as interdependent.

26 (MUR20; TRA23; GUR/TRB24; TRC/TRD/TRE/CHB17). Median buccal cuspule in the hypoflexid region between the trigonid and talonid (posterior molars) – (1) absent; (0) present.

27 (MUR21; TRA24; GUR/TRB25; TRC/TRD/TRE/CHB18). Molar roots – (1) covered by buccal expansion of the enamel layer (= indistinct and smoothly graded smoothly into crown); (0) buccal enamel restricted to crown (= distinct and separated from crown by marked constriction).

28 (MUR22; TRA25; GUR/TRB26; TRC/TRD/TRE/CHB129+134+139+148). Cristid obliqua morphology on m3 – (1) terminating lingually against the metaconid; (0) terminating buccally against the posterior part of the trigonid at or close to the protoconid. The cristid obliqua of peramelemorphians either terminates centrally against the posterior margin of the trigonid basin, or lingually against the metaconid. It also varies along the molar row, being most evident in the posterior molars (primarily the m3), which are often less modified by wear permitting unambiguous delimitation. The ‘curving’ or ‘straight’ definitions added by Muirhead^28^, and later incorporated by Travouillon *et al*.^18^, are not readily distinguishable. Moreover, we found differentiation of a lingual position relative to the ‘midpoint’, as well as the accompanying variation between adjacent molars^17^, to be arbitrary and not employable here.

29 (MUR23; TRA26; GUR/TRB27; TRC/TRD/TRE/CHB130+135+140+149). Posthypocristid orientation on m1–3 – (1) perpendicular to molar row; (0) oblique to molar row. We did not treat polymorphisms as discrete states^18^, nor did we favour separate character coding of the posthypocristid orientation on isolated molars^17^. In fact, the latter seems to be consistent irrespective of its connection to either the hypoconulid or entoconid. These cusps are closely adjacent on the m4 (character 30), thus the direction and termination of the posthypocristid were extremely difficult to ascertain.

30 (MUR24; TRA27; GUR/TRB28; TRC/TRD/TRE/CHB19). Hypoconulid position relative to the entoconid on m1–3; (1) directly posterior to entoconid; (0) posterobuccal to entoconid. State distribution for hypoconulid positioning closely approximates both relative elevation of the hypoconulid (character 24) and orientation of the posthypocristid (character 29). However, slight differences were observed and the character thus remained parsimony informative.

31 (MUR25; TRA28; GUR/TRB29; TRC/TRD/TRE/CHB20). Talonid size on the m4 – (1) entoconid and hypoconid small with poorly defined crests; (0) entoconid and hypoconid prominent with clearly defined crests. We eliminated the ‘smaller than state 0’ subdivision introduced by Travouillon *et al*.^18^ because of subjectivity. Note also that this character is biased by wear.

32 (MUR26; TRA29; GUR/TRB30; TRC/TRD/TRE/CHB21). Premaxilla shape – (1) longer than tall with premaxilla-nasal contact longer than maxilla-nasal contact; (0) taller than long with maxilla-nasal contact longer than premaxilla-nasal contact.

33 (MUR27; TRA30; GUR/TRB31; TRC/TRD/TRE/CHB22). Width of the nasals – (1) consistent along length; (0) broadening markedly beyond the premaxilla-nasal suture.

34 (MUR28; TRA31; GUR/TRB32; TRC/TRD/TRE/CHB23). Posterior extension of the nasals – (1) do not extend posteriorly; (0) extend posteriorly past the anterior orbital rim. Muirhead’s^28^ terms, ‘just’ anterior to nasals, and ‘well’ anterior of nasals are influenced by the internal tapering of the internasal suture, which may extend close to the orbital margin in taxa with state ‘2’. Consequently, we redefined this character to incorporate only two states, and also acknowledged both condition of the nasals in a dorsal and lateral perspective (contra^18^), and its developmental variability^98^.

35 (MUR30; TRA33; GUR/TRB34; TRC/TRD/TRE/CHB25). Jugal-maxilla contact – (1) invades zygomatic arch with jugal wings enclosing posterior maxillary flange (bifid); (0) invades maxilla and extends onto facial region of skull (not bifid).

36 (MUR31; TRA34; GUR/TRB35; TRC/TRD/TRE/CHB26). Lacrimal crest along the orbital rim – (1) present and distinct; (0) absent. Note that we eliminated the subjective intermediate state ‘1’ of Travouillon *et al*.^18^, which described a ‘partially developed’ crest.

37 (MUR32; TRA35; GUR/TRB36; TRC/TRD/TRE/CHB27). Antorbital fossa – (1) present; (0) absent. Relative depths of the antorbital fossae introduced by Muirhead^28^, and followed by Travouillon *et al*.^18^, are subjective. Travouillon *et al*.^14^ also found that relative dimensions of the antorbital fossa varied with ontogeny. We therefore limited this character to an unequivocal binary discrimination.

38 (MUR33; TRA36; GUR/TRB37; TRC/TRD/TRE/CHB28). Orbitiosphenoid – (1) markedly reduced or absent; (0) large and well developed. Relative size estimations of the orbitosphenoid as ‘small’ versus ‘very small’^18,28^ are equivocal. Character was therefore redefined with clearer binary division.

39 (MUR34; TRA38; GUR/TRB39; TRC/TRD/TRE/CHB29). Lateral wall of the neurocranium – (1) with squamosal-frontal contact; (0) alisphenoid-parietal contact.

40 (MUR35; TRA39; GUR/TRB40; TRC/TRD/TRE/CHB30). Disposition of the sphenorbital fissure and foramen rotundum – (1) sphenorbital fissure grossly larger than the foramen rotundum, with the latter opening directly into the skull; (0) sphenorbital fissure equal in size to the foramen rotundum, with the latter enclosed and ‘tunnel-like’. The varying descriptions of foramen rotundum shape and sphenorbital fissure disposition successively envisaged by Muirhead^28^, Travouillon *et al*.^18^ and Travouillon *et al*.^17^ were difficult to differentiate. Our state descriptions therefore compared only the proportions and relative enclosure of each foramen.

41 (MUR36+37; TRA39; GUR/TRB40; TRC/TRD/TRE/CHB155+156). Palatal vacuity number – (1) three, one in the incisial region (= incisive foramen), one in the premolar region (= accessory palatal fenestra), one in the molar region (= maxillopalatine fenestra); (0) two, one in the incisial region, one in the molar region. Muirhead^28^ combined the various states for septate palatal vacuities and palatal vacuity number into a twin character with ambiguous coding. To simplify this, we separated and rescored these features as two discrete characters^18^ based on presence/absence criteria. Travouillon *et al*.^17^ further divided these same states into distinct characters (TRC/TRD/TRE/CHB155+156), but this imparted artificial weighting to a correlated character system^63^

42 (MUR36+37; TRA40; GUR/TRB41; TRC/TRD/TRE/CHB32). Septate palatal vacuities – (1) absent; (0) present.

43 (MUR38; TRA41; GUR/TRB42; TRC/TRD/TRE/CHB33). Postglenoid foramen – (1) bounded medially (at least in part) by the periotic; (0) bounded entirely by either the squamosal, or squamosal and alisphenoid.

44 (MUR39; TRA42+43; GUR/TRB43+44; TRC/TRD/TRE/CHB33+34). Foramen ovale – (1) double perforation comprising both the primary and secondary foramen ovale; (0) single foramen ovale present. In an attempt to clarify the frequently confused terminology applied to the trigeminal nerve openings of the skull in peramelemorphians, Travouillon *et al*.^18^ coded morphology of the primary and secondary foramen ovale as two separate characters. However, their respective scores were virtually identical. To eliminate this redundancy, together with other ambiguities including the periotic separation of the foramen ovale (state ‘1’)^18^ which could not be detected in our specimens and is not preserved in *Yarala burchfieldi* (p. 514, figure 4.4)^21^, we restricted our state definitions to the easily identifiable presence/absence of a single or double perforation.

45 (MUR40; TRA44; GUR/TRB45; TRC/TRD/TRE/CHB36). Ectotympanic – (1) very prominent and thickened into a raised rim; (0) not prominent or thickened. The states descriptions ‘somewhat thickened’ and ‘further thickened’, as prescribed by Muirhead^28^ and Travouillon *et al*.^18^ are subjective. We therefore refined our character definition to capture only unequivocal expansion of the ectotympanic wing.

†46 (MUR41-43; TRA45+46; GUR/TRB46+47; TRC/TRD/TRE/CHB37+38). Alisphenoid tympanic process – (2) tympanic process completely encloses the tympanic cavity; (1) tympanic process encloses anterior section of the tympanic cavity; (0) tympanic process does not enclose the tympanic cavity. We found the characters/states of Muirhead^28^ and Travouillon *et al*.^18^ difficult to conceptualise because relative position of the foramen ovale is consistently medial rather than either lateral or medial (e.g. *Isoodon*); anterior tapering of the alisphenoid tympanic wing is also not always present. Moreover Travouillon *et al*.^18^ added aspects of alisphenoid tympanic process shape (TRA46; TRB47; TRC/TRD/TRE/CHB38) to again describe relative closure of the auditory bulla. We alternatively combined these subdivisions into a clearly identifiable sequence – the alisphenoid tympanic wing either fully, partially, not enclosing the tympanic cavity.

47 (MUR44-45; TRA45; GUR/TRB46). Hypotympanic sinus – (1) inflated to form a distinct cavity; (0) not inflated. The states defined by Muirhead^28^ for this character could not be unanimously identified in our specimens. Travouillon *et al*.^18^ further incorporated relative enlargement of the hypotympanic sinus within their TRA45. However, we found relative development of the alisphenoid tympanic process and hypotympanic sinus to be independent, and thus treated then as discrete characters.

48 (MUR46; TRA48; GUR/TRB49). Roof of the tympanic cavity – (1) formed by the alisphenoid and petrosal; (0) formed by the alisphenoid, petrosal and squamosal.

49 (MUR47-48; TRA49; GUR/TRB50; TRC/TRD/TRE/CHB40). Epitympanic recess – (1) deeply excavated and bowl-shaped; (0) shallow and indistinct. The characters of Muirhead^28^ and Travouillon *et al*.^18^ used subjective increments (e.g. ‘wider and deeper than’) to designate states. We alternatively preferred a binary coding to capture the range of observed morphologies.

50 (MUR49-50; TRA50; GUR/TRB51; TRC/TRD/TRE/CHB41). Squamosal epitympanic sinus – (1) well defined and deep; (0) poorly defined and shallow or absent. We found the paired characters of Muirhead^28^ and multiple state subdivisions of Travouillon *et al*.^18^ difficult to differentiate. Our definition therefore accommodates only relative excavation of the epitympanic sinus.

51 (MUR51; TRA51; GUR/TRB52; TRC/TRD/TRE/CHB42). Supraoccipital shape – (1) height greater than or equal to width; (0) width greatly exceeding height.

52 (MUR52). Paracondylar process – (1) prominent; (0) indistinct. Muirhead [2] named this structure the ‘paraoccipital process’ but we follow the terminology of Voss & Jansa^26,67^ and Wible^101^, who described the paracondylar process as extending ventrally from the exoccipital. Muirhead^28^ also scored estimated length of the paracondylar process, which we eliminated because of ambiguity.

**Craniodental characters adapted from Voss & Jansa^26,67^.**

53 (VJA35; VJB57; GUR/TRB55; TRC/TRD/TRE/CHB45). Sagittal crest (osteologically mature individuals only) – (1) absent; (0) present. Voss & Jansa^67^ initially developed this character using a presence/absence coding that was later modified by Voss and Jansa^26^ to include an additional intermediate state for didelphids: ‘sagittal crest small, not extending to frontals’ (terminology *sensu* Travouillon *et al*.^17^). Voss & Jansa (p. 33)^26^ further reported that an alternative condition, ‘sagittal crest large and extending to frontals’, exists in some dasyuromorphians and peramelemorphians (e.g. *Macrotis* spp.). Conversely, Travouillon *et al*.^17^ and Travouillon *et al*.^13^ scored sagittal crest extent in peramelemorphians as polymorphic. This concurs with our observations, which likewise detected considerable ontogenetic variation^98^.

54 (VJA49; VJB83; AHL11; GUR/TRB69; TRC/TRD/TRE/CHB59). Supraoccipital contribution to the foramen magnum – (1) exoccipitals contact each other medially, excluding the supraoccipital from the foramen magnum; (0) contributes to the superior margin of the foramen magnum. Aplin *et al*.^34^ reported exclusion of the supraoccipital from the foramen magnum as an autapomorphy of *Peroryctes broadbenti*. On the other hand, Travouillon *et al*.^19^ scored this feature in *Echymipera clara*, *Isoodon obesulus*, and *I. macrourus* (note that Travouillon *et al*.^17^, Travouillon *et al*.^13^ and Travouillon *et al*.^14^ subsequently eliminated the condition in their scores for *I. obesulus*, and *I. macrourus* = ‘0’). The dorsomedial external extremities of the exoccipitals are indeed proximal in these taxa. However, we found that they did not necessarily exclude the supraoccipital from the foramen magnum in osteologically immature specimens^98^. As an example, medial contact between the exoccipitals in *I. macrourus* ontogenetically progresses through continuous growth of the bony laminae even after endochondral ossification has been completed. The *incisura occipitalis* is ultimately closed off in a process similar to that reported in some didelphids^26^.

55 (VJA50; VJB85; GUR/TRB58; TRC/TRD/TRE/CHB48). Number of mental foramina – (2) three; (1) two; (0) one. We conservatively treated this character as unordered contrary to Voss & Jansa (p. 166)^26^ who assumed a morphocline based upon plausibility. Note also that Voss & Jansa^26^ specifically reported the presence of three (state 2) mental foramina in *Perameles gunnii*, although, Travouillon *et al*.^19^ scored only two (state 1); we likewise recorded this latter condition in our samples (scored ‘1/2’).

56 (VJB89; GUR/TRB59; TRC/TRD/TRE/CHB49). Shape of the I1 – (1) mesiodistally expanded and flat-crowned; (0) styliform or ‘chisel-like’.

57 (VJ116; GUR/TRB64; TRC/TRD/TRE/CHB54). Hypoconulid notch – (1) absent; (0) present in anterior cingulum of m2–4.

58 (VJ125; GUR/TRB66; TRC/TRD/TRE/CHB56). Posterior cingulid on m2–3 – (1) present; (0) absent.

**Cranial characters adapted from Aplin *et al*.^34^.**

59 (AHL6; TRB68; TRC/TRD/TRE/CHB58). Lacrimal foramen – (1) positioned on the lacrimal-maxillary suture; (0) wholly enclosed by the lacrimal. Note that paired lacrimal foramina can occur in didelphids and can be polymorphic in some populations of *Didelphis virginiana* (p. 30)^26^.

**Dental characters adapted from Travouillon *et al*.^18^.**

60 (GUR/TRB72; TRC/TRD/TRE/CHB61). C1 accessory cusps – (1) present; (0) absent. We acknowledge that accessory cusps might be modified and/or created by wear (see Voss & Jansa^26^, p. 48 for discussion).

**Dental characters adapted from Travouillon *et al*.^13,17^.**

61 (TRC/TRD/TRE/CHB84+94+105). Stylar cusp A – (1) cusp indistinct; (0) forms pronounced anterobuccal [parastylar] corner on at least one molar. Travouillon *et al*.^17^ separated the condition on each molar into discrete characters. We alternatively combined them because of interdependence. We also eliminated subjective extrapolations of cusp size by recording only marked reduction/loss versus development of a distinct cusp unanimously along molar row^99^.

62 (TRC/TRD/TRE/CHB121). Metacone on M4 – (1) absent; (0) recognisable as a distinct cusp or crest on metastylar corner of the tooth. Modification of the M4 metastylar region is consistent throughout both peramelemorphians and our selected outgroup taxa. However, *Macrotis* spp. exhibits extreme reduction resulting in loss of the metacone^17^. Because this is reflected in the modification of associated M4 structures (e.g. stylar cusp D, protocone: TRC/TRD/TRE/CHB122, TRC/TRD/TRE/CHB123 respectively), we limited the scope of our character definition to expression of the metacone only.

63 (TRC/TRD/TRE/CHB131+136+141). Posthypocristid buccal terminus on m1–3 – (1) terminates at entoconid; (0) terminates at hypoconulid. Travouillon *et al*.^17^ replicated this character for each individual molar, a procedure that was not advocated here. Note also that reduction of the talonid on m4 usually results in loss of an obvious hypoconulid (character 24).

**Novel craniodental characters.**

64. Talonid elevation in lateral view – (1) elevated to an equal height with the trigonid; (0) talonid positioned well below the level of the trigonid. This was derived from the unusual molar morphology found in the holotype (SAM P13645) of *Ischnodon australis*^37^. Its expression was recognizable irrespective of tooth wear.

65. Snout length – (1) ≥ 2x the snout width at the antorbital region; (0) < 2x the snout width at the antorbital region. Note that this correlated with the ‘snout very narrow’ state used by Groves and Flannery^11^ to define Peroryctidae (see GF Table 2, b1). We also employed a width parameter to eliminate interdependence with diastemata development (character 6) and extension of the nasals (character 34).

**Novel postcranial characters.**

66. Dorsal spines on the lumbar vertebrae – (1) craniocaudally narrow and ‘spine-like’ (length much less than 0.5 times the height); (0) craniocaudally elongate and ‘blade-like’ (length at least 0.5 times the height).

67.Ventral profiles of the craniad lumbar centra – (1) bear a distinct median keel; (0) smooth and rounded.

68. Acromion process of the scapula – (1) low and rounded; (0) prominent and extensively anterodorsally produced.

69. Supraspinous fossa of the scapula – (1) longitudinally shortened relative to the infraspinous fossa; (0) longitudinally elongate and of equal length to the infraspinous fossa.

70. Coracoid process of the scapula – (1) reduced and lacking a constricted neck; (0) large with a clearly constricted neck.

71. Supinator crest on the humerus – (1) absent or extremely reduced; (0) developed as distinct flange.

72. Deltoid crest on the humerus – (1) uniform in height along its length and with the distal extremity forming a shallow incline; (0) not uniform in height and with the distal extremity forming a steep incline that may bear a rugose boss at its apex.

73. Entepicondyle of the humerus – (1) reduced and flush with the trochlea; (0) separated from the trochlea by a distinct groove.

74. Coronal depression on the humerus – (1) perforated; (0) not perforated.

75. Shaft on the radius – (1) transversely compressed proximally; (0) proximally rounded*.*

76. Olecranon process of the ulna – (1) proximally projected with length 0.5 > height; (0) proximally short with length equal to height*.*

†77. Manual digits – (2) I, IV and V vestigial or absent (functionally didactyl); (1) I, V greatly reduced (functionally tridactyl); (0) no digits greatly reduced (pentadactyl).

78. Distal apex of the ilium – (1) transversely compressed and dorsoventrally expanded/’blade-like’; (0) transversely thickened and dorsoventrally rounded.

79. Condition of the femoral head – (1) domed with a semicircular cross-section, and perpendicular orientation relative to the long axis of the femoral shaft; (0) spherical with a circular cross-section, and oblique orientation relative to the long axis of the femoral shaft.

80. Lesser trochanter of the femur – (1) reduced to a small ridge or knob; (0) prominent and projecting as a broad flange.

81. Posterior surface of the femoral shaft – (1) bearing a prominent midline boss for muscle attachment; (0) the midline boss is only very weakly developed or absent.

82. Distal femoral sulcus – (1) deeply incised; (0) shallow.

†83. Distal portion of the fibula shaft – (2) fused to tibia; (1) ‘splint-like’, becoming concave at the distal extremity; (0) robust and ovoid throughout its cross-section.

84. Tibia shaft – (1) sinuous in abaxial view; (0) straight in abaxial view.

86. Tibial crest – (1) distinct with a deep abaxial tibial fossa; (0) weakly developed with a very shallow abaxial tibial fossa.

86. Lateral shelf for the astragalar facet on the calcaneum – (1) distally expanded (rectangular in outline); (0) distally short (triangular in outline).

87. Lateral trochlear ridge on the astragalus – (1) projected, giving a squared profile to the trochlea; (0) medially rounded, giving a rhomboidal profile to the trochlea.

88. Navicular facet on the astragalus – (1) short and distally directed; (0) elongate and ‘condyle-like’.

89. Pedal digit I – (1) absent; (0) present.

90. Pedal digits II and III – (1) syndactylous; (0) not syndactylous.

91. Metatarsal IV – (1) long and slender with its maximum length 0.3 > metatarsal V; (0) short and robust with its maximum length approximately equal to metatarsal V.

92. Proximal portion of metatarsal IV – (1) transversely expanded to accommodate plantar shift of metatarsals I-III and V; (0) transversely narrow with metatarsals III and V situated laterally.

93. Pedal digit V – (1) ‘splint-like’ (similar in form to II/III); (0) robust (similar in form to IV).

**Notes on excluded characters.**

X1 (MUR29; TRA32; GUR/TRB33; TRC/TRD/TRE/CHB23). Infraorbital canal length – (1) short infraorbital canal (shorter or equal to half the molar row); (0) long infraorbital canal (longer than half the molar row). This proved impossible to measure except via estimating length from the position of the maxillary foramen. Position of the infraorbital foramen relative to the P3 or M1 also varies in relative size of the opening, which can extend over the M1 root to the P3 making scoring uncertain. This condition is evident in almost all the extant bandicoots that we examined rendering the character untenable.

X2 (GUR/TRB47; TRC/TRD/TRE/CHB39). Morphology of the rostral tympanic process of the petrosal – (4) further dorsal extension of the ventral margin of the rostral tympanic process relative to state 3, resulting in lateral wall to the hypotympanic sinus within the process; (3) ventral margin of the rostral tympanic process of the petrosal extends laterally and slightly dorsally, resulting in the formation of a distinct hypotympanic sinus within the rostral tympanic process itself; (2) rostral tympanic process of the petrosal further enlarged, forming elongate crest-like process that extends the length of the promotorium and forms posteromedial wall of the hypotympanic sinus; medial margin of petrosal overlaps basioccipital; (1) rostral tympanic process of the petrosal forms a distinct projecting process that partially walls the posteromedial margin of the hypotympanic sinus; (0) rostral tympanic process of the petrosal absent or very small. Qualitative incremental enlargement of the rostral component of the tympanic process was difficult to score accurately and therefore excluded to eliminate ambiguity.

X3 (VJ52; GUR/TRB53; TRC/TRD/TRE/CHB43). Postorbital processes – (1) present; (0) absent or indistinct. Travouillon *et al*.^18^ incorporated this character specifically to distinguish their fossil outgroup taxa *Mutpuricinus archbaldi* and *Barinya wangala*. Its original definition and terminology^26,67,101^ recognized a distinct postorbital process in some didelphids. We excluded it because of redundancy for our taxon selection, and also ontogenetic influence (p. 31)^26^.

X4 (VJ54; GUR/TRB54; TRC/TRD/TRE/CHB44). Left and right parietal suture – (1) partially or completely co-ossified suture; (0) median suture present. Excluded because of ontogenetic bias.

X5 (VJ59; GUR/TRB56; TRC/TRD/TRE/CHB46). Interparietal – (1) present; (0) absent. Travouillon *et al*.^18^ incorporated this character from Voss & Jansa^26^ to distinguish their specific fossil outgroup taxa *Mutpuricinus archbaldi* and *Barinya wangala*. The interparietal could not be easily delineated in the adult didelphids (*Didelphis virginiana*) and other outgroups used in our study, and further relied upon assumed absence in our ingroup peramelemorphians.

X6 (VJ82; GUR/TRB57; TRC/TRD/TRE/CHB47). Lambdoid sesamoids – (1) present; (0) absent. Voss & Jansa^26^ introduced this character to differentiate peramelemorphians, which seem to have uniquely possessed sesamoids within their lambdoid crests. However, these ossifications are identifiable only in immature individuals, in which the parietal-supraoccipital suture is incompletely fused^98^. We therefore chose to exclude this character rather than assume presence in all adult specimens.

X7 (VJ96; GUR/TRB61; TRC/TRD/TRE/CHB51). Relative height of P2 and P3 – (2) P3 taller than P2; (1) P2 and P3 subequal in height; (0) P2 taller than P3. Excluded because states were interdependent with character 2 of our analysis. Accurate differentiation of state increments relative to sexual dimorphism was also problematic (see discussion for X18).

X8 (VJ97; GUR/TRB62; TRC/TRD/TRE/CHB52). Posterior crest of P3 – (1) lacking posterior cutting edge; (0) well developed posterior cutting edge. Excluded because of interdependence with character 2 in this analysis.

X9 (VJ109; TRC/TRD/TRE/CHB65). Lingual cusp presence on lower incisors – (1) absent; (0) present. Voss & Jansa^26^ recognized the presence of lingual incisor cusps in many didelphids and dasyurids. Nevertheless, it was found to be either indistinct or absent in our outgroup taxa and was thus excluded because of inapplicability.

X10 (VJ114; GUR/TRB63; TRC/TRD/TRE/CHB53). Relative height of p2 and p3 – (2) p3 taller than p2; (1) p2 and p3 subequal in height; (0) p2 taller than p3. Excluded because state increments could not be discriminated relative to sexual dimorphism (see discussion for X18 below), tooth wear, and interdependence with character 14 of this analysis.

X11 (VJ120; GUR/TRB65; TRC/TRD/TRE/CHB55). Relative position of hypoconid to protoconid on m3 – (2) lingual to protoconid; (1) subequal to protoconid; (0) labially salient to protoconid. The character description introduced by Travouillon *et al*.^18^ included a subjective morphocline with the ‘sub-equal’ condition of state ‘1’ incorporating components of ‘0’ and ‘2’. In addition, we found variability relative to wear and anomalous scoring in Voss & Jansa^26^ relative to Travouillon *et al*.^18^ – compare state ‘0’ versus ‘1’ for *Echymipera kalubu*; ‘0’ versus ‘2’ for *Perameles gunni*.

X12 (AHL3; GUR/TRB70). Inferior petrosal sinus opening – (1) enclosed by petrosal and basioccipital so not open ventrally; (0) open slit in ventral view between petrosal and basioccipital. Travouillon *et al*.^18^ derived this character from the diagnosis compiled for *Peroryctes* spp. by Aplin *et al*.^34^. However, their modified state definitions and distributions did not concur with our observations, which revealed obverse scores for state ‘1’ in *Echymipera kalubu* and *E. clara*. The original definition of this character (p. 22)^34^ also reported relative ‘enclosure’ of the inferior petrosal sinus via progressive reduction of the convergent angle between the petrosal and basioccipital. This is difficult to code accurately, thus we elected exclusion to avoid ambiguity.

X13 (GUR/TRB71; TRC/TRD/TRE/CHB60). Shape of I2–4 – (1) rhomboidal; (0) mesiodistally expanded and flat-crowned. We found these state definitions difficult to visualize and discriminate. Furthermore, the derived condition is autapomorphic for *Chaeropus ecaudatus* and parsimony uninformative in current data sets^13–19^.

X14 (GUR/TRB73; TRC/TRD/TRE/CHB62). Lower molar crown height – (1) lower molar height is significantly higher on buccal side than lingual side; (0) lower molars are as high on the buccal side as they are on the lingual side. Identification alternative states for this character are often obscured by wear. In accordance, we found relative cusp height to be highly variable between consecutive teeth in the molar row. Note that Travouillon *et al*.^18^ recorded *Ischnodon australis* as displaying state ‘1’; however, all of the cusps on the m1 and m2 of the holotype and only known specimen (SAM P13645) are heavily worn, and of approximately equal height (the m2 hypoconid is also broken: p. 250, fig. 2)^37^.

X15 (TRC/TRD/TRE/CHB31). Presence of accessory fenestrae – (1) absent; (0) present. The meaning of this anatomical description is unclear, but presumably correlates with the palatal openings. Excluded because of descriptive ambiguities and inconsistent scoring relative to presumed correlation with the palatal openings.

X16 (TRC/TRD/TRE/CHB64). Size of I3 – (1) I3 is larger than I2 and I4; (0) I2–4 are the same size. Although there is often minute gradational size increase in the I2–4 of peramelemorphians (e.g. Aplin *et al*.^34^ p. 16, fig 6A,B), this is not sufficiently marked or consistently distinguishable to enable accurate state coding.

X17 (TRC/TRD/TRE/CHB67). Shape of lower canine – (1) accessory cusps are present on either side of the major cusp of c1; (0) single-rooted unicusped lower canine. We were unable to reconcile published scores^13,17^ with our sample of specimens. Voss & Jansa^26^ reported double rooted ‘premolariform’ canines in some didelphids. The c1 of peramelemorphians is otherwise unanimously single-rooted. Accessory ‘cusp-like’ structures can also develop as a function of wear making coding misleading.

X18 (TRC/TRD/TRE/CHB68). Size of the upper or lower canine – (1) small, about as high as premolars; (0) large, raised above premolars. Aplin *et al*.^34^ (2010) reported that canine size, as well as relative dimensions of the premolars (especially the P3/p3) and molars, are sexually dimorphic in peramelemorphians with males showing significant proportional increase^14^. Because this biases character scoring, and is particularly misleading when sex of the sampled individual is unknown (as in fossils), we excluded all qualitative proportional characters that described size-based comparisons along the tooth row.

X19 (TRC/TRD70). Length of P1 – (2) P1 is longer than P2; (1) P1 is as long as P2; (0) P1 is shorter than P2. See comment for X18.

X20 (TRC/TRD/TRE/CHB71). Anterior cusp of P1 and/or P2 – (1) tall distinct cusp; (0) small remnant or no cusp. The anterior cusp on both P1/p1 and P2/p2 is often heavily worn, making distinction between states ambiguous. In accordance, we detected inconsistencies in the scores produced by Travouillon *et al*.^17^ and Travouillon *et al*.^13^. To avoid assuming the condition for those taxa in which unworn juvenile dentitions were not available, we excluded all characters that coded relative size of the premolar accessory cusps (TRC/TRD77, TRC/TRD78, TRC/TRD81).

X21 (TRC/TRD/TRE/CHB73). Morphology of the central cusp of P1 and P2 – (1) central cusp is inflated widening the tooth anteriorly; (0) central cusp not inflated. State differentiation was ambiguous and therefore rejected.

X22 (TRC/TRD/TRE/CHB76). Length of p1 – (1) p1 is as long as p2 or p3; (0) p1 is shorter than either p2 or p3. See comment for X18.

X23 (TRC/TRD/TRE/CHB77). Anterior cusp of p1 – (1) tall distinct cusp; (0) none or small remnant. See comment for X20.

X24 (TRC/TRD/TRE/CHB78). Anterior cusp of p2 – (1) tall distinct cusp; (0) none or small remnant. See comment for X20.

X25 (TRC/TRD/TRE/CHB81). Reclining of p3 – (1) the anterior root of p3 is more exposed than the posterior root, reclining p3 toward m1; (0) the anterior and posterior roots of p3 are level. Exposure of the premolar roots occurs to varying degrees in peramelemorphians, although, Muirhead & Filan^20^ specifically noted its occurrence in *Yarala burchfieldi* and some species of *Echymipera*. We also detected it in other extant taxa (e.g. *Rhynchomeles prattorum* and *Perameles nasuta*: p. 7, fig 3b; p. 9, fig 4d)^28^, but Travouillon *et al*.^17^ and Travouillon *et al*.^13^ only scored its presence in *Y. burchfieldi*, *Galadi* spp. and *Bulungu palara*. In addition, we found that the p3 does not recline *per se*. Rather, increasing exposure of the root reveals its oblique orientation.

X26 (TRC/TRD/TRE/CHB85). Morphology of StB/StC on M1 – (3) StB and StC are fused and oval in shape connected by a stylar crest but difficult to clearly identify each cusp; (2) StB and StC are fused and oval in shape connected by a stylar crest but clearly identifiable as separate cusp; (1) StB and StC are distinct cusps; (0) no StC or B. We found these state descriptions difficult to detect, especially with elimination of stylar cusp C by wear (see also TRC/TRD122). Subjective ‘oval’ versus ‘conical’ shape discriminations (TRC/TRD95, TRC/TRD106), and relative ‘large/tall’ and ‘small’ size estimations (TRC/TRD102, TRC/TRD112, TRC/TRD116) applied to consecutive molars by Travouillon *et al*.^17^, could likewise not be unambiguously visualized.

X27 (TRC/TRD/TRE/CHB89+100+110+120). Termination of postprotocrista [/posthypocrista] on M1 [M2; M3; M4] – (4) postprotocrista joins with the posterior cingulum and ends at the lingual flank of the metastylar tip; (3) postprotocrista joins with the posterior cingulum and ends posteriorly to the midpoint of the postmetacrista; (2) postprotocrista ends on posterolingual flank of metacone [postprotocrista ends posterior to the most posterior end of the postparacrista for M4]; (1) postprotocrista ends on lingual flank of metacone [postprotocrista ends level with the most posterior end of the postparacrista for M4]; (0) postprotocrista ends on anterior flank of metacone [postprotocrista ends anterior to the most posterior end of the postparacrista for M4]. This character was affected by the relative development of the metaconule, whose expansion into a prominent cusp scores dependently with incremental shift of the postparacrista termination against and beyond the metacone^13,17^. Extent of the postparacrista on the M4 is similarly influenced by modification of the metastylar region, which we assessed differently here.

X28 (TRC/TRD/TRE/CHB91+103+113). StD1 on M1 [M2; M3] – (1) absent; (0) present. Turnbull *et al*.^12^ first identified an accessory cuspule (D1) associated with stylar cusp D in cf. *Peroryctes tedfordi*. Travouillon *et al*.^18^ and Travouillon *et al*.^13^ subsequently scored this trait in peroryctines, as well as *Bulungu palara*. However, we noticed an overlap of scores with the remnant centrocrista ‘spurs’ documented in *Peroryctes* spp. (p. 21, figure 10A)^34^. Because these structures cannot be conclusively differentiated, especially in the apparently lost upper molars cf. *P. tedfordi*, we excluded this character as a potentially homologous set.

X29 (TRC/TRD117). Connection of StA and StB on M4 – (1) crest connecting the two cusps; (0) no connection. We could confirm, and thus excluded, the scores of Travouillon *et al*.^17^ and Travouillon *et al*.^13^, who recognized state ‘1’ in *Echymipera clara* and *Peroryctes raffrayana*.

X30 (TRC/TRD/TRE/CHB119). Postparacrista of M4 – (1) postparacrista curves as a small centrocrista and ends just after the centrocrista; (0) postparacrista straight. Excluded because ‘curving’ versus ‘straight’ state definitions were equivocal.

X31 (TRC/TRD/TRE/CHB125). Hypoflexid on m1 – (1) present; (0) absent. State scores for this character were incompatible with our sampled specimens, all of which displayed some form of flexid between the protoconid and hypoconid. Travouillon *et al*.^17^ seem to have described the relative depth of the flexid; however this requires qualitative estimation, which we avoided here.

X32 (TRC/TRD/TRE/CHB152). Buccal shelf on m4 – (1) buccal shelf buccally reduced (hypoconid more buccal than shelf); (0) buccal shelf end at buccal side of hypoconid. Interpretation of states can be influenced by optical perspective and were therefore excluded as ambiguous.

**Morphological data matrix.**

Didelphis

000000000000000000000000000000000000000000000000000001100000000000000010000000100000000000000

Antechinus

010110021010000000000000100011011001100100001211110010100100100000100011000101000000000000000

Dasyurus

000000022000000000002000000000000000000000001000000000100100100000000000000000000000000000000

Chaeropus_ecaudatus

000101011000111110111011100111011111111111101211111100111001000001111111111121111120111111111

Echymipera_clara

(01)1100102100011001111001111001101111011100001011000110111100000000????????????????????????????

Echymipera_kalubu

011011021011111011110011100011011110111000010110001100111000000011110100001111011111101101010

Echymipera_rufescens

01101102101011001011001111001101111011100001011000110011100100001111110011111111110110111100?

Isoodon_auratus

01000101101111111011001111001101111111111110121111110011100000000????????????????????????????

Isoodon_macrourus

010001011011111110110111111111011111111100101211110101111000000101100111011111111111111101010

Isoodon_obesulus

011001011011111110110111111111011111111101101211111101111000000001111101011111111111111101010

Macrotis_lagotis

010101100101110001111021111011011111101001111211111100211010110011111100001111000121101111110

Macrotis_leucura

0101011011011111011110(12)1111011011111101001111211111110211010010011111100001111000121101111110

Microperoryctes_ornata

010011010000110010110001010011111111101010010110000110111001100011100100011111011111101101010

Microperoryctes_papuensis

011011010000110010110(01)0101001111111010101001011000011011100000001????????????????????????????

Microperoryctes_longicauda_Sol

01101101100011001011000111001111111010101001011000011011100000001????????????????????????????

Microperoryctes_sp_Tembagapura

01101101100011001011100111001111111010101001011000011011100000001????????????????????????????

Perameles_bougainville

011001011010110010110111110011011111111111111211111110(12)11000000001111111111111111111111111000

Perameles_eremiana

01110101101011101011001111001111111111111(01)10121111011011100000000????????????????????????????

Perameles_gunnii

010001011010110011110011110111011111111101111111111110(12)11000001001100110011111111111111101010

Perameles_nasuta

01100101101011101111011111011101111111110111121111111021100000(01)001111110011111111111111101010

Peroryctes_broadbenti

101001011011110(01)10(01)1000111001111111011110001000000110111101000001????????????????????????????

Peroryctes_raffrayana

10101101000(01)1(01)0010110001000001011110111000010110000110111010000011000101111111011111111101010

Rhynchomeles_prattorum

01101102101011011011010111001101111010100001011000011011101000001????????????????????????????

Bulungu_palara

??101?0?00111100010?00011100110??11?1000?010?1100001101?10??0000?????????????????????????????

Bulungu_campbelli

???????????0?1?0001???111100111?????????????????????????10??0?00?????????????????????????????

Crash_bandicoot

?????1???011111111(01)???????0?????????????????????????????????0????????????????????????????????

Galadi_amplus

0?00??0?10111110011?00011100110?11101??010??????????0?1?100000000????????????????????????????

Galadi_speciosus

00000?001001111000(01)?0001110011?1111010000010?0000001001?100000000????????????11001???????????

Ischnodon_australis

?????????????????????011110011??????????????????????????10????11?????????????????????????????

Lemdubuoryctes_aruensis

??1???0?101(01)1(01)00010?00011100010?????1?????????0?00????0?10??0000?????????????????????0???????

Liyamayi_dayi

??????0?????110?1?1????11????1??????????????????????????????0?00?????????????????????????????

Kutjamarcoot_brevirostrum

??????0?10111100011???111?00110???????????????????????1?10??0?00?????????????????????????????

Madju_variae

0?101?0?00111100101100011100110?111010101011?111000?0?(12)?100000000????????????????????????????

Perameles_bowensis

??????0?101?110?111???11110011??????????????????????????10??0?(01)0?????????????????????????????

Perameles_sobbei

??????0????0?100111?0011110111????????????????????????1?1???0?(01)0?????????????????????????????

Peroryctes_tedfordi

??????0?001?110?001??0110100?1?????????????????0????????10??0?0??????????????????????????????

Yarala_burchfieldi

0?00000?00111100010100011000111?10101000?01???10000100??101000000????????????????????????????

Yarala_kida

??????0??01111000(01)0???010?0011??????????????????????????10??0000?????????????????????????????

**List of skeletal specimens and reference works.** Institutional Abbreviations: AM, Australian Museum, Sydney, Australia; ANU, Australian National University Zoological Collection, Canberra, Australia; LTU, La Trobe University Zoological Collection, Melbourne, Australia; NMV, Museum Victoria, Melbourne, Australia; NTM, Northern Territory Museum and Art Gallery, Darwin, Northern Territory, Australia; QM, Queensland Museum, Brisbane, Australia; SAM, South Australian Museum, Adelaide, Australia; UCMP, University of California Museum of Paleontology, Berkeley, U.S.A.; WAM, Western Australian Museum, Perth, Australia.

**Outgroup taxa.**

*Antechinus minimus*

LTU unregistered, no data.

*Dasyurus maculatus*

NMV C1010, Snowy River Gorge, Victoria, Australia.

NMV C6099, Wynyard, Tasmania, Australia.

NMV C6116, Wynyard, Tasmania, Australia (postcranial skeleton).

NMV C6117, Wynyard, Tasmania, Australia.

NMV C6120, Wynyard, Tasmania, Australia (postcranial skeleton).

NMV C34755, Amboyne crossing area, Victoria, Australia (skull and skeleton).

*Didelphis virginiana*

NMV C2201, Santa Clara, California, USA (skull and skeleton).

NMV C11129, East Baton Rouge Park, Louisiana, USA (skull and skeleton).

NMV C15434, East Baton Rouge Park, Louisiana, USA (skull and skeleton).

**Extant and recently extinct ingroup taxa.**

*Chaeropus ecaudatus*

AM P422, Murray River, South Australia.

NMV C468, central Australia, Northern Territory, Australia.

NMV C470, central Australia, Northern Territory, Australia.

NMV C2900, Junction of Murray and Darling Rivers, South Australia (skull and skeleton).

*Echymipera clara*.

AM M17232, Yapsiei Station, West Sepik Province, Papua New Guinea.

*Echymipera kalubu*.

AM M13766, mountain behind Kyemana village, Yapsiei area, West Sepik Province, Papua New Guinea.

AM 13736 PNG, Tibi village, Skgonga River, Yapsiei area, West Sepik Province, Papua New Guinea.

AM M24590, Hollandia, Papua New Guinea (includes partial postcranium).

AM M24591, Hollandia, Papua New Guinea (includes partial postcranium).

NMV C33108, no data.

*Echymipera rufescens*

AM M24521, Jimi Valley, Western Highlands Province, Papua New Guinea.

AM 32580, Oksapmin, West Sepik Province, Papua New Guinea.

*Isoodon auratus*

AM M2437, no data.

*Isoodon macrourus*

AM M33980, Gloucester, New South Wales, Australia.

AM M3777, Comboyne, northeastern New South Wales, Australia.

NMV C7492, Gosford, New South Wales, Australia (skull and skeleton).

NMV C7773, 20.8km West of Cooktown, Queensland, Australia (skull and skeleton).

NMV C26185, Victoria, Australia (skull and skeleton).

*Isoodon obesulus*

AM M22582, Woy Woy, New South Wales, Australia.

AM M11135, Terry Hills entrance of Kurin-gai National Park, Sydney, Australia.

NMV C7257, Silvan, Victoria, Australia (skull and skeleton).

NMV C7774, Ecklin South, Victoria, Australia (skull and skeleton).

NMV C10304, Pomonal South, Victoria, Australia (skull and skeleton).

NMV C25091, Healesville Sanctuary, Victoria, Australia (skull and skeleton).

*Macrotis lagotis*

AM M37523, no data (includes partial postcranium).

AM M22184, captive bred, Taronga Zoo, Sydney, Australia (includes partial postcranium).

AM S420, Bourke, New South Wales, Australia.

*Macrotis leucura*

AM M21673, Charlotte Water, central Australia.

NMV C7943, Lake Eyre District, South Australia (skull, axial skeleton and skin).

*Microperoryctes longicauda* ‘Sol’

AM M13729 Sol River, Telofomin region, West Sepik Province, Papua New Guinea.

*Microperoryctes* ‘*ornata*’

AM M37996, Lake Tawa, Papua New Guinea (includes partial postcranium).

Reference texts: Travouillon *et al*.^17^ reported that all labelled specimens of *M. ornata* currently held by the AM were actually misidentified individuals of *M. longicauda*. We therefore limited our scores for *M. longicauda* to the voucher specimen (AM M13729, *Microperoryctes longicauda* ‘Sol’), which was our tissue source for DNA. *Microperoryctes* ‘*ornata*’ is herein based upon the most complete available skeleton (AM M37996), which noticeably differed from AM M13729 in key scores.

*Microperoryctes papuensis*

WAM M21338, Fane, Papua New Guinea.

WAM M21337, Fane, Papua New Guinea.

WAM M21340, Fane, Papua New Guinea.

WAM M21339, Fane, Papua New Guinea.

*Microperoryctes* sp. ‘Tembagapura’

AM M30723, Tembagapura area, Forest behind BQ Barracks, Irian Jaya.

*Perameles bougainville*

AM M1122, Western Australia.

AM M2440, no data.

ANU M315, no data (skull and skeleton).

*Perameles eremiana*

SAM M3946, between Oolarinna and Everard Range, South Australia.

SAM M3975, no data.

Reference texts: Warburton & Travouillon^3^ listed both these specimens as potentially missidentified examples of *P. bougainville*. However, our morphological topologies invariably returned these terminals as either paraphyletic or unresolved with other taxa (see Supplementary Figs S13–S15), implying phylogenetic distinction. In acknowledgement, we provisionally retain their original taxonomic designation pending more detailed future analyses.

*Perameles gunnii*

AM M16196, near New Norfolk, Tasmania, Australia.

AM A6842, Railton, northwestern Tasmania, Australia.

AM M37522, no data (includes postcranial skeleton).

NMV C32880, Hamilton, Victoria, Australia (skull and skeleton).

NMV C32883, Hamilton, Victoria, Australia (skull and skeleton).

NMV C32884, Hamilton, Victoria, Australia (postcranial skeleton).

NMV C32885, Hamilton, Victoria, Australia (skull and skeleton).

*Perameles nasuta*

AM S1073, Elizabeth Bay, Sydney, Australia (includes partial postcranium).

AM S1961, Epping, Sydney, Australia.

AM S1072, Elizabeth Bay, Sydney, Australia (includes partial postcranium).

NMV C26670, Codoben District, Victoria, Australia (skull and skeleton).

NMV C7350, Bete Bolong, Victoria, Australia (skull and skeleton).

NMV C28406, Strathbogie, 4.5km NNE at Creek Junction Rd and Spring Creek Rd junction, Victoria, Australia (skull and skeleton).

*Peroryctes broadbenti*

AM M3238 (Type), Port Moresby, Papua New Guinea.

*Peroryctes raffrayana*

AM M18446, Agofa village, Southern Highlands Province, Papua New Guinea.

AM M30817, Kwiyawgi area, Kelancurri, Irian Jaya (includes partial postcranium).

*Rhynchomeles prattorum*

AM M29415, Seram.

**Fossil ingroup taxa.**

*Bulungu campbelli* (‘*Lenniratherium milleri*’)

SAM P13853 (Holotype, cast), Ngapakaldi Quarry, Lake Ngapakaldi, Tirari Desert, South Australia. Horizon/age: Ngapakaldi Local Fauna, Zone C, Etadunna Formation, upper Oligocene.

Reference texts: Travouillon *et al*.^17^. Muirhead^28^ renamed this species under a different generic epithet without justification. Its formal description was otherwise only available in thesis format^102^, with a simultaneous release of both the genus and species names in an internationally distributed printed volume of extended dissertation abstracts^103^. Although this satisfied the criteria for published works as required under the ICZN for establishment of a valid taxon (see ICZN, Chapter 3, Articles 8 and 9: www.iczn.org), the lack of a viable diagnosis renders its usage informal. Attribution to *Bulungu* was also made without specific discussion of priority^17^. We therefore included the taxon as a test of its nomenclatural distinction.

*Bulungu palara*

QM F23437 (Holotype), Upper Site, Riversleigh World Heritage Area, Queensland, Australia. Horizon/age: Zone B, Riversleigh Cenozoic limestone sequence, lower Miocene.

QM F23436 (Paratype), Upper Site, Riversleigh World Heritage Area, Queensland, Australia. Horizon/age: Zone B, Riversleigh Cenozoic limestone sequence, lower Miocene.

QM F52993, Camel Sputum Site, Riversleigh World Heritage Area, Queensland, Australia. Horizon/age: Zone B, Riversleigh Cenozoic limestone sequence, lower Miocene.

QM F53025, Rat Vomit Site, Riversleigh World Heritage Area, Queensland, Australia. Horizon/age: Zone B, Riversleigh Cenozoic limestone sequence, lower Miocene.

QM F53586, Upper Site, Riversleigh World Heritage Area, Queensland, Australia. Horizon/age: Zone B, Riversleigh Cenozoic limestone sequence, lower Miocene.

Reference texts: Gurovich *et al*.^16^; Travouillon *et al*.^17^. Gurovich *et al*.^16^ reported additional specimens of *B. palara* both the Quantum Leap and Lee Sye’s Outlook (LSO) sites, which have been correlated with the late Oligocene Riversleigh Faunal Zone A. However, Arena *et al*.^104^ recently included Quantum Leap Site within their revised early Miocene Zone B (B2/B3) interval. Given the unresolved age of LSO, we provisionally limit the stratigraphical range of *B. palara* to the lower Miocene pending further confirmation.

*Crash bandicoot*

QM F56245 (Holotype), Allan’s Ledge 1990 (AL90) Site, Riversleigh World Heritage Area, Queensland, Australia. Horizon/age: Zone C, Riversleigh Cenozoic limestone sequence, middle Miocene.

Reference text: Travouillon *et al*.^13^.

*Galadi speciosus*

QM F23393 (Holotype), Upper Site, Riversleigh World Heritage Area, Queensland, Australia. Horizon/age: Zone B, Riversleigh Cenozoic limestone sequence, lower Miocene.

QM F24681 (Paratype), Quantum Leap Site, Riversleigh World Heritage Area, Queensland, Australia. Horizon/age: Zone A, Riversleigh Cenozoic limestone sequence, upper Oligocene.

QM F31363, Quantum Leap Site, Riversleigh World Heritage Area, Queensland, Australia. Horizon/age: Zone A, Riversleigh Cenozoic limestone sequence, upper Oligocene.

QM F36352, Microsite, Riversleigh World Heritage Area, Queensland, Australia. Horizon/age: Zone B, Riversleigh Cenozoic limestone sequence, lower Miocene.

Reference texts: Travouillon *et al*.^18,19^.

*Ischnodon australis*

SAM P13645 (Holotype), Woodard Quarry, Lake Palankarinna, Tirari Desert, South Australia. Horizon/age: Palankarinna Local Fauna, Mampuwordu Sands, tentatively upper Miocene–lower Pliocene.

Reference text: Stirton^37^.

*Kutjamarcoot brevirostrum*

UCMP100275 (Holotype), Leaf Locality, Lake Ngapakaldi, Tirari Desert, South Australia. Horizon/age: Kutjamarpu Local Fauna, Wipajiri Formation, lower Miocene.

UCMP100281 (Paratype), Leaf Locality, Lake Ngapakaldi, Tirari Desert, South Australia. Horizon/age: Kutjamarpu Local Fauna, Wipajiri Formation, lower Miocene.

UCMP100282 (Paratype), Leaf Locality, Lake Ngapakaldi, Tirari Desert, South Australia. Horizon/age: Kutjamarpu Local Fauna, Wipajiri Formation, lower Miocene.

UCMP102442 (Paratype), Leaf Locality, Lake Ngapakaldi, Tirari Desert, South Australia. Horizon/age: Kutjamarpu Local Fauna, Wipajiri Formation, lower Miocene.

SAM P17917 (Paratype), Leaf Locality, Lake Ngapakaldi, Tirari Desert, South Australia. Horizon/age: Kutjamarpu Local Fauna, Wipajiri Formation, lower Miocene.

SAM P50824 (Paratype), Leaf Locality, Lake Ngapakaldi, Tirari Desert, South Australia. Horizon/age: Kutjamarpu Local Fauna, Wipajiri Formation, lower Miocene.

SAM P50826 (Paratype), Leaf Locality, Lake Ngapakaldi, Tirari Desert, South Australia. Horizon/age: Kutjamarpu Local Fauna, Wipajiri Formation, lower Miocene.

SAM P50827 (Paratype), Leaf Locality, Lake Ngapakaldi, Tirari Desert, South Australia. Horizon/age: Kutjamarpu Local Fauna, Wipajiri Formation, lower Miocene.

SAM P50832 (Paratype), Leaf Locality, Lake Ngapakaldi, Tirari Desert, South Australia. Horizon/age: Kutjamarpu Local Fauna, Wipajiri Formation, lower Miocene.

Reference text: Chamberlain *et al*.^15^.

*Lemdubuoryctes aruensis* gen. et sp. nov.

WAM 14.9.6 (Holotype), Liang Lemdubu cave, Pulau Kobroor, Aru Islands, Eastern Indonesia. Horizon/age: ‘Layer 4’, Spit 24, ‘Lemdubu Local Fauna’, late Pleistocene–Holocene.

WAM 14.9.1 (Paratype), Liang Lemdubu cave, Pulau Kobroor, Aru Islands, Eastern Indonesia. Horizon/age: ‘Layer 4’, Spit 21, ‘Lemdubu Local Fauna’, late Pleistocene–Holocene.

WAM 14.9.3 (Paratype), Liang Lemdubu cave, Pulau Kobroor, Aru Islands, Eastern Indonesia. Horizon/age: ‘Layer 4’, Spit 21, ‘Lemdubu Local Fauna’, late Pleistocene–Holocene.

WAM 14.9.7 (Paratype), Liang Lemdubu cave, Pulau Kobroor, Aru Islands, Eastern Indonesia. Horizon/age: ‘Layer 3’, Spit 6, ‘Lemdubu Local Fauna’, late Pleistocene–Holocene.

WAM 14.9.9 (Paratype), Liang Lemdubu cave, Pulau Kobroor, Aru Islands, Eastern Indonesia. Horizon/age: ‘Layer 4’, Spit 21, ‘Lemdubu Local Fauna’, late Pleistocene–Holocene.

WAM 14.9.11 (Paratype), Liang Lemdubu cave, Pulau Kobroor, Aru Islands, Eastern Indonesia. Horizon/age: ‘Layer 4’, Spit 18, ‘Lemdubu Local Fauna’, late Pleistocene–Holocene.

WAM 14.9.15 (Paratype), Liang Lemdubu cave, Pulau Kobroor, Aru Islands, Eastern Indonesia. Horizon/age: ‘Layer 4’, Spits 19 and 18 respectively, ‘Lemdubu Local Fauna’, late Pleistocene–Holocene (isolated petrosals).

WAM 14.9.16 (Paratype), Liang Lemdubu cave, Pulau Kobroor, Aru Islands, Eastern Indonesia. Horizon/age: ‘Layer 5’, Spit 25 and ‘Layer 4’, Spits 23, 22 and 20 sequentially, ‘Lemdubu Local Fauna’, late Pleistocene–Holocene (isolated calcanea).

WAM 14.9.2, Liang Lemdubu cave, Pulau Kobroor, Aru Islands, Eastern Indonesia. Horizon/age: ‘Layer 4’, Spit 21, ‘Lemdubu Local Fauna’, late Pleistocene–Holocene

WAM 14.9.4, Liang Lemdubu cave, Pulau Kobroor, Aru Islands, Eastern Indonesia. Horizon/age: ‘Layer 4’, Spit 21, ‘Lemdubu Local Fauna’, late Pleistocene–Holocene.

WAM 14.9.5, Liang Lemdubu cave, Pulau Kobroor, Aru Islands, Eastern Indonesia. Horizon/age: ‘Layer 4’, Spit 21, ‘Lemdubu Local Fauna’, late Pleistocene–Holocene.

WAM 14.9.8, Liang Lemdubu cave, Pulau Kobroor, Aru Islands, Eastern Indonesia. Horizon/age: ‘Layer 3’, Spit 6, ‘Lemdubu Local Fauna’, late Pleistocene–Holocene.

WAM 14.9.10, Liang Lemdubu cave, Pulau Kobroor, Aru Islands, Eastern Indonesia. Horizon/age: ‘Layer 4’, Spit 18, ‘Lemdubu Local Fauna’, late Pleistocene–Holocene.

WAM 14.9.12, Liang Lemdubu cave, Pulau Kobroor, Aru Islands, Eastern Indonesia. Horizon/age: ‘Layer 4’, Spit 14, ‘Lemdubu Local Fauna’, late Pleistocene–Holocene.

WAM 14.9.13–14.9.14, Liang Lemdubu cave, Pulau Kobroor, Aru Islands, Eastern Indonesia. Horizon/age: ‘Layer 4’, Spits 19 and 18 respectively, ‘Lemdubu Local Fauna’, late Pleistocene–Holocene (isolated petrosals).

WAM 14.9.17–14.9.20, Liang Lemdubu cave, Pulau Kobroor, Aru Islands, Eastern Indonesia. Horizon/age: ‘Layer 5’, Spit 25 and ‘Layer 4’, Spits 23, 22 and 20 sequentially, ‘Lemdubu Local Fauna’, late Pleistocene–Holocene (isolated calcanea).

Reference texts: Warburton & Travouillon^3^; Aplin & Pasveer^30^.

*Liyamayi dayi*

QM F56246 (Holotype), Rick’s Sausage Site, Riversleigh World Heritage Area, Queensland, Australia. Horizon/age: Zone C, Riversleigh Cenozoic limestone sequence, middle Miocene.

QM F56247 (Paratype), Rick’s Sausage Site, Riversleigh World Heritage Area, Queensland, Australia. Horizon/age: Zone C, Riversleigh Cenozoic limestone sequence, middle Miocene.

QM F56250 (Paratype), Rick’s Sausage Site, Riversleigh World Heritage Area, Queensland, Australia. Horizon/age: Zone C, Riversleigh Cenozoic limestone sequence, middle Miocene.

Reference text: Travouillon *et al*.^13^.

*Madju variae*

QM F57978 (Holotype), Wayne’s Wok Site, Riversleigh World Heritage Area, Queensland, Australia. Horizon/age: Zone B, Riversleigh Cenozoic limestone sequence, lower Miocene (includes undescribed postcranial elements^11^).

QM F36336 (Paratype), Allan’s Ledge 1990 (AL90) Site, Riversleigh World Heritage Area, Queensland, Australia. Horizon/age: Zone C, Riversleigh Cenozoic limestone sequence, middle Miocene.

QM F57321 (Paratype), Allan’s Ledge 1990 (AL90) Site, Riversleigh World Heritage Area, Queensland, Australia. Horizon/age: Zone C, Riversleigh Cenozoic limestone sequence, middle Miocene.

QM F57322 (Paratype), Upper Site, Riversleigh World Heritage Area, Queensland, Australia. Horizon/age: Zone B, Riversleigh Cenozoic limestone sequence, lower Miocene.

QM F57323 (Paratype), Wayne’s Wok Site, Riversleigh World Heritage Area, Queensland, Australia. Horizon/age: Zone B, Riversleigh Cenozoic limestone sequence, lower Miocene.

QM F57324 (Paratype), Allan’s Ledge 1990 (AL90) Site, Riversleigh World Heritage Area, Queensland, Australia. Horizon/age: Zone C, Riversleigh Cenozoic limestone sequence, middle Miocene (includes undescribed postcranial elements^11^).

QM F57437 (Paratype), Allan’s Ledge 1990 (AL90) Site, Riversleigh World Heritage Area, Queensland, Australia. Horizon/age: Zone C, Riversleigh Cenozoic limestone sequence, middle Miocene.

QM F23405, Inabeyance Site, Riversleigh World Heritage Area, Queensland, Australia. Horizon/age: Zone B, Riversleigh Cenozoic limestone sequence, lower Miocene.

QM F23406, Camel Sputum Site, Riversleigh World Heritage Area, Queensland, Australia. Horizon/age: Zone B, Riversleigh Cenozoic limestone sequence, lowere Miocene.

QM F23428 (Paratype), Neville’s Garden Site, Riversleigh World Heritage Area, Queensland, Australia. Horizon/age: Zone B, Riversleigh Cenozoic limestone sequence, lower Miocene.

QM F23429 (Paratype), Camel Sputum Site, Riversleigh World Heritage Area, Queensland, Australia. Horizon/age: Zone B, Riversleigh Cenozoic limestone sequence, lower Miocene.

QM F23430 (Paratype), Camel Sputum Site, Riversleigh World Heritage Area, Queensland, Australia. Horizon/age: Zone B, Riversleigh Cenozoic limestone sequence, lower Miocene.

QM F23431 (Paratype), Camel Sputum Site, Riversleigh World Heritage Area, Queensland, Australia. Horizon/age: Zone B, Riversleigh Cenozoic limestone sequence, lower Miocene.

QM F24227 (Paratype), Camel Sputum Site, Riversleigh World Heritage Area, Queensland, Australia. Horizon/age: Zone B, Riversleigh Cenozoic limestone sequence, lowere Miocene.

Reference text: Travouillon *et al*.^14^.

*Perameles bowensis*

AM F98809 (Holotype), unnamed road cutting on the Merriwa−Cassilis Road, 12km West of Merriwa, New South Wales, Australia. Horizon/age: Bow Local Fauna, lower Pliocene.

AM F98811 (Paratype), unnamed road cutting on the Merriwa−Cassilis Road, 12km West of Merriwa, New South Wales, Australia. Horizon/age: Bow Local Fauna, lower Pliocene.

AM F98812 (Paratype), unnamed road cutting on the Merriwa−Cassilis Road, 12km West of Merriwa, New South Wales, Australia. Horizon/age: Bow Local Fauna, lower Pliocene.

AM F98813 (Paratype), unnamed road cutting on the Merriwa−Cassilis Road, 12km West of Merriwa, New South Wales, Australia. Horizon/age: Bow Local Fauna, lower Pliocene.

AM F69804, Wellington Caves, Wellington, New South Wales, Australia. Horizon/age: Big Sink Local Fauna, Big Sink Beds, lower Pliocene.

AM F69887, Wellington Caves, Wellington, New South Wales, Australia. Horizon/age: Big Sink Local Fauna, Big Sink Beds, lower Pliocene.

AM F69896, Wellington Caves, Wellington, New South Wales, Australia. Horizon/age: Big Sink Local Fauna, Big Sink Beds, lower Pliocene.

AM F69897, Wellington Caves, Wellington, New South Wales, Australia. Horizon/age: Big Sink Local Fauna, Big Sink Beds, lower Pliocene.

AM F69899, Wellington Caves, Wellington, New South Wales, Australia. Horizon/age: Big Sink Local Fauna, Big Sink Beds, lower Pliocene.

Reference texts: Muirhead^28^; Muirhead *et al*.^42^. Note that we did not score from the paratype M2, AM F98810, because Turnbull *et al*.^12^ (p. 523) suggested that it might be derived from a different taxon.

*Perameles sobbei*

QM F43878 (Holotype), QM L796, Kings Creek, southern Darling Downs, Queensland, Australia. Horizon/age: upper Pleistocene.

QM F43879 (Paratype), QM L796, Kings Creek, southern Darling Downs, Queensland, Australia. Horizon/age: upper Pleistocene.

QM F43880 (Paratype), QM L796, Kings Creek, southern Darling Downs, Queensland, Australia. Horizon/age: upper Pleistocene.

QM F21680, QM L796, Kings Creek, southern Darling Downs, Queensland, Australia. Horizon/age: upper Pleistocene.

QM F44569, QM L796, Kings Creek, southern Darling Downs, Queensland, Australia. Horizon/age: upper Pleistocene.

Reference texts: Price^38,105^.

cf. *Peroryctes tedfordi*

NMV P157329 (holotype), Grange Burn, 7km West of Hamilton, Victoria, Australia. Horizon/age: Hamilton Local Fauna, unnamed rock unit, lower Pliocene.

NMV P54144, Grange Burn, 7km West of Hamilton, Victoria, Australia. Horizon/age: Hamilton Local Fauna, unnamed rock unit, lower Pliocene.

NMV P157131, Grange Burn, 7km West of Hamilton, Victoria, Australia. Horizon/age: Hamilton Local Fauna, unnamed rock unit, lower Pliocene.

NMV P157133, Grange Burn, 7km West of Hamilton, Victoria, Australia. Horizon/age: Hamilton Local Fauna, unnamed rock unit, lower Pliocene.

NMV P157136, Grange Burn, 7km West of Hamilton, Victoria, Australia. Horizon/age: Hamilton Local Fauna, unnamed rock unit, lower Pliocene.

NMV P157139, Grange Burn, 7km West of Hamilton, Victoria, Australia. Horizon/age: Hamilton Local Fauna, unnamed rock unit, lower Pliocene.

NMV P180042, Grange Burn, 7km West of Hamilton, Victoria, Australia. Horizon/age: Hamilton Local Fauna, unnamed rock unit, lower Pliocene.

NMV P210911, Grange Burn, 7km West of Hamilton, Victoria, Australia. Horizon/age: Hamilton Local Fauna, unnamed rock unit, lower Pliocene.

NMV P210912, Grange Burn, 7km West of Hamilton, Victoria, Australia. Horizon/age: Hamilton Local Fauna, unnamed rock unit, lower Pliocene.

NMV P210975, Grange Burn, 7km West of Hamilton, Victoria, Australia. Horizon/age: Hamilton Local Fauna, unnamed rock unit, lower Pliocene.

Reference text: Turnbull *et al*.^12^.

*Yarala burchfieldi*

QM F16860 (Holotype), Upper Site, Riversleigh World Heritage Area, Queensland, Australia. Horizon/age: Zone B, Riversleigh Cenozoic limestone sequence, lower Miocene.

QM F16861, Upper Site, Riversleigh World Heritage Area, Queensland, Australia. Horizon/age: Zone B, Riversleigh Cenozoic limestone sequence, lower Miocene.

QM F16862, Upper Site, Riversleigh World Heritage Area, Queensland, Australia. Horizon/age: Zone B, Riversleigh Cenozoic limestone sequence, lower Miocene.

QM F16863, Upper Site, Riversleigh World Heritage Area, Queensland, Australia. Horizon/age: Zone B, Riversleigh Cenozoic limestone sequence, lower Miocene.

QM F16864, Camel Sputum Site, Riversleigh World Heritage Area, Queensland, Australia. Horizon/age: Camel Sputum Local Fauna, Zone B(B2), Riversleigh Cenozoic limestone sequence, lower Miocene.

QM F23385, Upper Site, Riversleigh World Heritage Area, Queensland, Australia. Horizon/age: Zone B(B2), Riversleigh Cenozoic limestone sequence, lower Miocene.

Reference texts: Muirhead^21,28^; Muirhead & Filan^20^.

*Yarala kida*

NTM P892-25 (Holotype), NTM Site, Kangaroo Well, Northern Territory, Australia. Horizon/age: Kangaroo Well Local Fauna, Ulta Limestone, upper Oligocene.

NTM P892-26 (Paratype), NTM Site, Kangaroo Well, Northern Territory, Australia. Horizon/age: Kangaroo Well Local Fauna, Ulta Limestone, upper Oligocene.

NTM P892-3, NTM Site, Kangaroo Well, Northern Territory, Australia. Horizon/age: Kangaroo Well Local Fauna, Ulta Limestone, upper Oligocene.

NTM P892-4, NTM Site, Kangaroo Well, Northern Territory, Australia. Horizon/age: Kangaroo Well Local Fauna, Ulta Limestone, upper Oligocene.

NTM P892-9, NTM Site, Kangaroo Well, Northern Territory, Australia. Horizon/age: Kangaroo Well Local Fauna, Ulta Limestone, upper Oligocene.

NTM P892-55, NTM Site, Kangaroo Well, Northern Territory, Australia. Horizon/age: Kangaroo Well Local Fauna, Ulta Limestone, upper Oligocene.

Reference text: Schwartz^22^.

**Parsimony apomorphy lists for ingroup fossils and named crown clades.** Character and state numbers were derived from the combined morphology + DNA topology of crown taxa with fossils added sequentially in generic/chronostratigraphical order. All transformations were unambiguous irrespective of ACCTRAN or DELTRAN optimisation. Monophyly of Peramelidae, together with its fossil exemplars *Crash bandicoot*^13^ and *Perameles bowensis*^42^, was not supported by our character data. *Alternative placement of extant clade based on morphology only.

Peramelemorphia (Crown Perameloidea)

6 (0==>1), 13 (0==>1), 14 (0==>1), 19 (0==>1), 20 (0==>1), 23 (0==>1), 24 (0==>1), 34 (0==>1), 35 (0==>1), 39 (0==>1), 42 (0==>1), 43 (0==>1), 51 (0==>1), 52 (0==>1), 56 (0==>1), 57 (0==>1), 66 (0==>1), 68 (0==>1), 69 (0==>1), 70 (0==>1), 75 (0==>1), 77 (0==>1), 82 (0==>1), 83 (0==>2), 85 (0==>1) 87 (0==>1), 88 (0==>1), 90 (0==>1), 92 (0==>1)

Peramelemorphia (Including fossils)

12 (0==>1), 13 (0==>1), 14 (0==>1), 20 (0==>1), 24 (0==>1), 35 (0==>1), 43 (0==>1), 52 (0==>1)

*Yarala*

11 (0==>1)

*Yarala burchfieldi* + Perameloidea

25 (0==>1)

Perameloidea (Crown only)

6 (0==>1), 19 (0==>1), 23 (0==>1), 36 (0==>1), 39 (0==>1), 42 (0==>1), 48 (0==>1), 49 (0==>1), 50 (0==>1), 51 (0==>1)

*Yarala + Bulungu palara*

11 (0==>1), 18 (0==>1)

*Bulungu palara + Galadi +Lemdubuoryctes aruensis +* Perameloidea

11 (0==>1), 18 (0==>1)

*Galadi +* Perameloidea

9 (0==>1), 15 (0==>1), 19 (0==>1)

*Galadi speciosus +* Perameloidea

11 (1==>0), 18 (1==>0)

*Bulungu campbelli +Madju variae +* Perameloidea

36 (0==>1), 39 (0==>1), 42 (0==>1), 46 (0==>2), 48 (0==>1), 49 (0==>1), 50 (0==>1), 51 (0==>1)

*Bulungu campbelli +* *M. variae + Kutjamarcoot brevirostrum + Crash bandicoot +* Perameloidea

17 (0==>1), 18 (1==>0)

*Bulungu campbelli +* *K. brevirostrum + C. bandicoot +* Perameloidea

23 (0==>1), 36 (0==>1), 42 (0==>1), 46 (1==>2) 49 (0==>1), 50 (0==>1), 51 (0==>1)

*Galadi amplus + Bulungu campbelli +* *M. variae + K. brevirostrum + C. bandicoot + Liyamayi dayi +* *Ischnodon australis +* Perameloidea

19 (0==>1)

*Bulungu campbelli +* *M. variae + K. brevirostrum + C. bandicoot + L. dayi + I. australis +* Perameloidea

17 (0==>1)

Chaeropodidae

16 (0==>1), 28 (0==>1), 40 (0==>1), 41 (0==>1), 60 (0==>1), 73 (0==>1), 77 (1==>2)

*Chaeropodidae + Peramelinae

8 (2==>1), 28 (0==>1), 79 (0==>1), 86 (0==>1)

Thylacomyidae (Crown only)

7 (0==>2), 10 (0==>1), 18 (0==>1), 27 (0==>1), 55 (1==>2), 59 (0==>1), 62 (0==>1)

*Thylacomyidae (*Macrotis leucura* included) + Chaeropodidae + Peramelinae

40 (0==>1), 71 (0==>1), 86 (0==>1)

Thylacomyidae *+* *K. brevirostrum*

17 (1==>0), 18 (0==>1)

Peramelinae

40 (0==>1)

*Perameles eremiana* + *B. campbelli*

31 (0==>1)

*Perameles gunni* + *Perameles nasuta + Perameles sobbei*

18 (0==>1), 28 (0==>1)

Peroryctinae

1 (0==>1), 2 (1==>0), 23 (1==>0), 59 (0==>1)

Peroryctinae + cf. *Peroryctes tedfordi*

1 (0==>1), 2 (1==>0), 59 (0==>1)

*Peroryctes raffrayana* + cf. *Peroryctes tedfordi*

9 (0==>1), 25 (0==>1)

Echymiperinae

8 (1==>2)

Peroryctinae + Echymiperinae

36 (1==>0), 42 (1==>0), 43 (1==>0), 45 (1==>0), 45 (1==>0), 46 (2==>1), 48 (1==>0), 49 (1==>0), 50 (1==>0), 69 (1==>0)

*Madju variae* + Peroryctinae + Echymiperinae

5 (0==>1), 15 (1==>0), 36 (0==>0), 42 (1==>0), 46 (2==>1), 49 (1==>0), 50 (1==>0), 51 (1==>0)

**List of taxon area distribution codes and reference works.** Generalized vegetation units^50^: A = rainforest; B = woodland (referring primarily to sclerophyll forests); C = shrubland (including ‘open’ and xeromorphic vegetation); and D = arid/semi-arid vegetation and desert.

**Outgroup taxa.**

*Aepyprymnus*

Distribution: dry wooded areas and open *Eucalyptus* forests in coastal Queensland and New South Wales.

Area coding: (B).

Reference text: Groves^106^; Van Dyck & Strahan^51^.

*Antechinus*

Distribution: rainforests and humid coastal woodlands from the Northern Territory and far northern Queensland through to southeastern Victoria and Tasmania, and South Australia to southwestern Western Australia.

Area coding: (AB).

Reference text: Van Dyck & Strahan^51^; Groves^107^.

*Cercartetus*

Distribution: alpine rainforests, humid woodlands and coastal scrublands in West Papua (Irian Jaya) and Papua New Guinea to far northeastern Queensland, northwestern Victoria and southeastern South Australia (including Kangaroo Island), Tasmania and southwestern Western Australia.

Area coding: (ABC).

Reference text: Flannery^35^; Van Dyck & Strahan^51^; Groves^106^.

*Dasyurus*

Distribution: diverse habitats from rainforest to woodlands, and xeric scrub to desert grasslands. Ranges from West Papua (Irian Jaya) and Papua New Guinea, across northern Western Australia, the Northern Territory and Queensland through Central Australia and eastern coasts to southeastern Victoria and Tasmania.

Area coding: (ABCD).

Reference text: Flannery^35^; Van Dyck & Strahan^51^; Groves^107^.

*Didelphis*/Didelphinae

Distribution: wooded habitats throughout eastern and central North America from southern Canada to México and Central America south to Argentina.

Area coding: (AB).

Reference text: Voss & Jansa^26^; Gardner^108^.

*Dromiciops*

Distribution: humid mountain forests in Chile and Argentina.

Area coding: (A).

Reference text: Voss & Jansa^26^; Gardner^109^.

Macropodinae

Distribution: diverse habitats from rainforest to woodlands, and xeric scrub to desert grasslands. Ranges from West Papua (Irian Jaya) and Papua New Guinea, across northern Western Australia, the Northern Territory and Queensland through Central Australia and eastern coasts to southeastern Victoria and Tasmania.

Area coding: (ABCD).

Reference text: Flannery^35^; Van Dyck & Strahan^51^; Groves^107^.

*Myrmecobius*

Distribution: *Eucalyptus* woodland in southwestern Western Australia. Historically ranged through semiarid woodland and grassland across southern South Australia and western New South Wales.

Area coding: (BC).

Reference text: Van Dyck & Strahan^51^; Groves^107^.

*Notoryctes*

Distribution: sandy deserts in Central and northwestern Western Australia.

Area coding: (D).

Reference text: Van Dyck & Strahan^51^; Groves^110^.

*Petaurus*

Distribution: Moluccas and Aru archipelagos of Indonesia, across West Papua (Irian Jaya) and Papua New Guinea to Bismark Archipelago. Australian range from northeastern Western Australia, northern Northern Territory and Queensland through to southeastern South Australia. Introduced into Tasmania.

Area coding: (AB).

Reference text: Groves^106^; Van Dyck & Strahan^51^; Flannery^33,111^.

*Phalanger*

Distribution: rainforest and woodland from Timor, Moluccas and Aru archipelagos of Indonesia, across West Papua (Irian Jaya) and Papua New Guinea to Bismark Archipelago, Solomon Islands, Sulu Buru and Halmahera.

Area coding: (AB).

Reference text: Groves^106^; Flannery^35,111^.

*Phascogale*

Distribution: fragmented woodland habitats from southwestern Western Australia across southern Australia to southeastern New South Wales to northern Queensland the Northern Territory and northwestern Western Australia.

Area coding: (B).

Reference text: Van Dyck & Strahan^51^; Groves^107^.

*Phascolarctos*

Distribution: *Eucalyptus* woodland in southeastern Queensland, eastern New South Wales and southeastern South Australia and Victoria.

Area coding: (B).

Reference text: Groves^106^; Van Dyck & Strahan^51^.

*Phascolosorex*

Distribution: mountain rainforest in the Vogelkop Peninsula, northwestern West Papua (Irian Jaya), Indonesia.

Area coding: (A).

Reference text: Groves^107^; Flannery^35,111^.

*Planigale*

Distribution: diverse habitats from rainforest to woodlands, and xeric scrub to desert grasslands. Ranges from Central Province of Papua New Guinea into northern Australia from northeastern Western Australia, the Northern Territory and Queensland to south-central Queensland and northern New South Wales.

Area coding: (ABCD).

Reference text: Van Dyck & Strahan^51^; Groves^107^.

Pseudocheiridae

Distribution: rainforest and woodlands from West Papua (Irian Jaya) and Papua New Guinea, across northern Western Australia, the Northern Territory and Queensland, down through eastern coastal New South Wales to southern Victoria, Tasmania, Bass Strait islands, southern South Australia and southwestern Western Australia.

Area coding: (AB).

Reference text: Flannery^35^; Groves^105^; Van Dyck & Strahan^51^.

*Thylacinus*

Distribution: temperate rainforest and *Eucalyptus* woodland margins in Tasmania.

Area coding: (AB).

Reference text: Van Dyck & Strahan^51^; Groves^107^.

*Vombatus*

Distribution: primarily woodland in eastern New South Wales, through southern Victoria to southeastern South Australia, Tasmania and Bass Strait islands.

Area coding: (B).

Reference text: Groves^106^; Van Dyck & Strahan^51^.

**Ingroup taxa.**

*Chaeropus ecaudatus*

Distribution: arid grassland and open vegetation areas in Central Australia.

Area coding: (D).

Reference text: Warburton & Travouillon^3^; Groves^8^; Van Dyck & Strahan^51^.

*Echymipera clara*.

Distribution: rainforests in northern central New Guinea and Yapen Island, West Papua (Irian Jaya), Indonesia.

Area coding: (A).

Reference text: Warburton & Travouillon^3^; Groves^8^; Flannery^35^.

*Echymipera kalubu*.

Distribution: rainforest throughout New Guinea and surrounding islands in Bismark Archipelago as well as Yapan, Biak-Supiori, Waigeo, Misool and Salawati islands in Eastern Indonesia.

Area coding: (A).

Reference text: Warburton & Travouillon^3^; Groves^8^; Flannery^35,111^.

*Echymipera rufescens*

Distribution: rainforest and dry woodlands in far northeastern Queensland, throughout New Guinea and the D’Entrecasteaux islands of Papua New Guinea, and Kai and Aru archipelagos, Yapen and Misool islands in Eastern Indonesia.

Area coding: (AB).

Reference text: Warburton & Travouillon^3^; Groves^8^; Flannery^35,111^; Van Dyck & Strahan^51^.

*Isoodon auratus*

Distribution: dry grasslands to humid forest and scrub in northwestern Western Australia and Barrow Island. Historical range included northern Western Australia and the Northern Territory.

Area coding: (BCD).

Reference text: Warburton & Travouillon^3^; Groves^8^; Van Dyck & Strahan^51^.

*Isoodon macrourus*

Distribution: dry grasslands to humid open forest and scrub in south and eastern Papaua New Guinea, northern Queensland, the Northern Territory, northeastern Western Australia, and south into eastern Queensland and northeastern New South Wales.

Area coding: (BCD).

Reference text: Warburton & Travouillon^3^; Groves^8^; Van Dyck & Strahan^51^.

*Isoodon obesulus*

Distribution: moist forest and dense shrublands along southern coastal margin from southeastern New South Wales and Victoria, Tasmania, and coastal South Australia to southwestern Western Australia.

Area coding: (BC).

Reference text: Warburton & Travouillon^3^; Groves^8^; Van Dyck & Strahan^51^.

*Macrotis lagotis*

Distribution: arid grassland and open vegetation areas in Central Australia with historical distribution into arid regions of northwestern Western Australia, southestern Queensland and northwestern New South Wales.

Area coding: (D).

Reference text: Warburton & Travouillon^3^; Groves^8^; Van Dyck & Strahan^51^.

*Macrotis leucura*

Distribution: arid areas of Central Australia.

Area coding: (D).

Reference text: Groves^8^; Van Dyck & Strahan^51^.

*Microperoryctes longicauda* ‘Sol’

Distribution: rainforest in West Sepik Province, northwestern Papua New Guinea.

Area coding: (A).

Reference text: Warburton & Travouillon^3^; Groves^8^; Flannery^35^.

*Microperoryctes* ‘*ornata*’

Distribution: rainforest in Kaijende Highlands, Enge Province, central-northern Papua New Guinea.

Area coding: (A).

Reference text: Groves^8^; Flannery^35^.

*Microperoryctes papuensis*

Distribution: rainforest in Milne Bay Province, far eastern Papua New Guinea.

Area coding: (A).

Reference text: Warburton & Travouillon^3^; Groves^8^; Flannery^35^.

*Microperoryctes* sp. ‘Tembagapura’

Distribution: rainforest in Tembagapura area, West Papua (Irian Jaya), Indonesia.

Area coding: (A).

Reference text: Groves^8^; Flannery^35^.

*Perameles bougainville*

Distribution: historically occurring in dry scrubland and grassland in western New South Wales and northwestern Victoria through southern South Australia and southern Western Australia. Today only surviving on Bernier and Dorre islands off Western Australia.

Area coding: (CD).

Reference text: Warburton & Travouillon^3^; Groves^8^; Van Dyck & Strahan^51^.

*Perameles eremeana*

Distribution: Central Australian grassland in northern South Australia, the Northern Territory and Western Australia.

Area coding: (D).

Reference text: Warburton & Travouillon^3^; Groves^8^; Van Dyck & Strahan^51^.

*Perameles gunnii*

Distribution: moist shrublands in Tasmania and historically in southern Victoria.

Area coding: (C).

Reference text: Warburton & Travouillon^3^; Groves^8^; Van Dyck & Strahan^51^.

*Perameles nasuta*

Distribution: coastal humid woodlands, shrublands and grasslands in from northeastern Queensland and New South Wales to southeastern Victoria.

Area coding: (BC).

Reference text: Warburton & Travouillon^3^; Groves^8^; Van Dyck & Strahan^51^.

*Peroryctes broadbenti*

Distribution: lowland forests and woodlands in Central Province, southeastern Papua New Guinea.

Area coding: (B).

Reference text: Warburton & Travouillon^3^; Groves^8^; Aplin *et al*.^34^; Flannery^35^.

*Peroryctes raffrayana*

Distribution: mountain rainforest in the Vogelkop Peninsula, northwestern West Papua (Irian Jaya), Indonesia.

Area coding: (A).

Reference text: Warburton & Travouillon^3^; Groves^8^; Aplin *et al*.^34^; Flannery^35^.

*Rhynchomeles prattorum*

Distribution: mountain forests on Seram Island, Moluccas Archipelago, Indonesia.

Area coding: (A).

Reference text: Warburton & Travouillon^3^; Groves^8^; Van Dyck & Strahan^51^; Flannery^111^.

**References**

1. Archer, M., *et al*. A new family of bizarre durophagous carnivorous marsupials from Miocene deposits in the Riversleigh World Heritage Area, northwestern Queensland. *Sci. Rep.* **6**, 26911 (2016).
2. Binfield, P., *et al*. A new Miocene carnivorous marsupial, *Barinya kutjamarpensis* (Dasyuromorphia), from central Australia. *Alcheringa* http://dx.doi.org/10.1080/03115518.2016.1180029 (2016).
3. Godthelp, H., Wroe, S. & Archer, A. A new marsupial from the early Eocene Tingamarra Local Fauna of Murgon, southeastern Queensland: a prototypical Australian marsupial? *J. Mammal. Evol.* **6**, 289−313 (1999).
4. Beck, R. M. D., Godthelp, H., Weisbecker, V., Archer, M. & Hand, S. J. Australia’s oldest marsupial fossils and their biogeographical implications. *PLoS ONE* **3**, e1858 (2008).
5. Horovitz, I., Martin, T., Bloch, J., Ladaveze, S., Kurz, C. & Sanchez-Villagra, M. R. Cranial anatomy of the earliest marsupials and the origin of opossums. *PLoS ONE* **4**: e8278 (2009).
6. Williamson, T. E. & Taylor, L. H. New species of *Peradectes* and *Swaindelphys* (Mammalia: Metatheria) from the Early Paleocene (Torrejonian) Nacimiento Formation, San Juan Basin, New Mexico, USA. *Palaeontologia Electronica* **14**, 1–16 (2011).
7. Case, J. A., Goin, F. J. & Woodburne, M. O. “South American” marsupials from the Late Cretaceous of North America and the origin of marsupial cohorts. *J. Mammal. Evol.* **11**, 223-255 (2004).
8. Wroe, S. The geologically oldest dasyurid (Marsupialia), from the middle Miocene of Riversleigh, northwestern Queensland. *Palaeontol*. **42**, 501–527 (1999).
9. Woodhead, J., *et al*. Developing a radiometrically-dated chronologic sequence for Neogene biotic change in Australia, from the Riversleigh World Heritage Area of Queensland. *Gond. Res*. **29**, 153–167 (2016).
10. Meredith, R. W., Westerman, M. & Springer, M. S. A phylogeny of Diprotodontia (Marsupialia) based on sequences for five nuclear genes. *Mol. Phylogenet. Evol*. **51**, 554–571 (2009).
11. Meredith, R. W., Mendoza, M. A., Roberts, K. K., Westerman, M. & Springer, M. S. (2010). A phylogeny and timescale for the evolution of Pseudocheiridae (Marsupialia: Diprotodontia) in Australia and New Guinea. *J. Mammal. Evol.* **17**, 75–99 (2010).
12. Case, J. A., Meredith, R. W. & Person, J. A pre-Neogene phalangerid possum from South Australia. *Mus. North. Ariz. Bull.* **65**, 659–675 (2009).
13. Kear, B. P., Cook, B. N., Archer, M. & Flannery, T. F. Implications of a new species of the Oligo-Miocene kangaroo (Marsupialia: Macropodoidea) *Nambaroo*, from the Riversleigh World Heritage Area, Queensland. *J. Paleontol*. **81**, 1147–1167 (2007).
14. Kear, B. P. & Pledge, N. S. A new fossil kangaroo from the Oligocene-Miocene Etadunna Formation of Ngama Quarry, Lake Palankarinna, South Australia. *Aust. J. Zool.* **55**, 331–339. (2008).
15. Black, K. H., Travouillon, K. J., Den Boer, W., Kear, B. P., Cooke, B. N. & Archer, M. A new species of the basal “kangaroo” *Balbaroo* and a re-evaluation of stem macropodiform interrelationships. *PloS ONE* **9**, e112705 (2014).
16. Meredith, R. W., Westerman, M. & Springer, M. S. A phylogeny and timescale for the living genera of kangaroos and kin (Macropodiformes: Marsupialia) based on nuclear DNA sequences. *Aust. J. Zool.* **56**, 395–410 (2009).
17. Maddison, D. R. & Maddison, W. P. *MacClade* ver.4.08a. http://macclade.org (2005).
18. Flores, D. A., Abdala, F. & Giannini, N. P. Post-weaning cranial ontogeny in two bandicoots (Mammalia, Peramelomorphia, Peramelidae) and comparison with carnivorous marsupials. *Zool.* **116**, 372−384 (2013).
19. Kangas, A. T., Evans, A. R., Thesleff, I., & Jernvall, J. Nonindependence of mammalian dental characters. *Nature* **432**, 211–214 (2004).
20. Wroe, S., Ebach, M., Ahyong, S., de Muizon, C. & Muirhead, J. Cladistic analysis of dasyuromorphian (Marsupialia) phylogeny using cranial and dental characters. *J. Mammal.* **81**,1008−1024 (2000).
21. Wible, J. R. On the cranial osteology of the short-tailed opossum *Monodelphis brevicaudata* (Didelphidae, Marsupialia). *Ann. Carnegie Mus.* **72**, 137–202 (2003).
22. Campbell, C. R. *Tertiary Dasyuridae and Peramelidae* (*Marsupialia*) *from the Tirari Desert, South Australia*. PhD thesis (University of California, 1976).
23. Campbell, C. R. *Tertiary Dasyuridae and Peramelidae* (*Marsupialia*) *from the Tirari Desert, South Australia*. *Dissert*. *Abstr*. *Int*. *Bull*. *Sci*. *Eng*. 37, 4375 (1976).
24. Arena, D. A. *et al*. Mammalian lineages and the biostratigraphy and biochronology of Cenozoic faunas from the Riversleigh World Heritage Area, Australia. *Lethaia* **49**, 43–60 (2016).
25. Price, G. J. Fossil bandicoots (Marsupialia, Peramelidae) and environmental change during the Pleistocene on the Darling Downs, southeastern Queensland, Australia. *J*. *Syst*. *Palaeont*. **2**, 347−356 (2004).
26. Groves, C. P. in *Mammal Species of the World: A Taxonomic and Geographic Reference*. 3^rd^ Edition 43−70 (Johns Hopkins University Press, 2005).
27. Groves, C. P. in *Mammal Species of the World: A Taxonomic and Geographic Reference*. 3^rd^ Edition 23−37 (Johns Hopkins University Press, 2005).
28. Gardner, A. L. in *Mammal Species of the World: A Taxonomic and Geographic Reference*. 3^rd^ Edition 3−18 (Johns Hopkins University Press, 2005).
29. Gardner, A. L. in *Mammal Species of the World: A Taxonomic and Geographic Reference*. 3^rd^ Edition 21 (Johns Hopkins University Press, 2005).
30. Groves, C. P. in *Mammal Species of the World: A Taxonomic and Geographic Reference*. 3^rd^ Edition 22 (Johns Hopkins University Press, 2005).
31. Flannery, T. F. *Mammals of the South-West Pacific & Moluccan Islands* (Cornell University Press, 1995).
